# Zebrafish behavioral assessment as a first-tier whole-organism NAM for developmental neurotoxicity: A multi-laboratory evaluation

**DOI:** 10.1016/j.neuro.2026.103504

**Published:** 2026-07-02

**Authors:** Lisa Truong, Jui-Hua Hsieh, Lee Ellis, Oihane Jaka, Arantza Muriana, Beatriz Molina-Martínez, Ana del Pozo, Valentina Schiavone, Vincenzo Di Donato, Claudia Miguel Sanz, Robyn L. Tanguay, Ellen Hessel, Kristen Ryan

**Affiliations:** a Department of Environmental and Molecular Toxicology, Oregon State University, Corvallis, OR, USA; b Division of Translational Toxicology, National Institute of Environmental Health Sciences, Research Triangle Park, NC, USA; c National Research Council of Canada, Halifax, NS, Canada; d BBD BioPhenix SLU - Biobide, San Sebastian, Gipuzkoa, Spain; e ZeClinics SL, Barcelona, Spain; f Centre for Health Protection, National Institute for Public Health and the Environment, Utrecht, Netherlands

**Keywords:** Zebrafish larvae, Light-Dark Transition Test (LDTT), Developmental Neurotoxicology (DNT), New Approach Methodologies (NAM), Tiered testing strategy, Regulatory toxicology

## Abstract

The development of New Approach Methodologies (NAMs) for developmental neurotoxicity (DNT) assessment requires demonstrating reproducibility across testing facilities and clearly defining their role in tiered testing strategies. This multi-laboratory study evaluated the zebrafish Light-Dark Transition Test (LDTT) across four independent laboratories testing 33 compounds (28 suspected DNT toxicants and 5 putative DNT-negatives), including organophosphate and pyrethroid insecticides (deltamethrin, parathion), flame retardants (TDCIPP, TMPP, BPDP), and neonicotinoids (nicotine, acetamiprid) using agreed-upon protocol elements. A novel three-tiered quality control framework removed 10.9% of plates passing basic viability criteria, providing a systematic approach applicable to multi-well plate-based behavioral assays. Developmental toxicity (DT) endpoints showed high inter-laboratory concordance (84–100%), while DNT activity calls were more variable (68–84%); however, potency estimates for active compounds showed similar reproducibility (mean 3.2-fold for DNT, 3.5-fold for DT). Zebrafish LDTT detected 93% of DNT-positive compounds and 100% of DNT-negative compounds as a single whole-organism assay, demonstrating complementary performance to the 13-assay DNT *in vitro* battery (DNT-IVB; 86% sensitivity, 60% specificity). Integration with DNT IATA case studies demonstrated that multi-laboratory zebrafish data strengthen weight-of-evidence evaluations, with independent validation establishing high confidence in the flame retardants TMPP and BPDP. Together, these findings support positioning zebrafish larval behavioral assays as an efficient first-tier whole-organism NAM that bridges mechanistic *in vitro* screening and mammalian guideline studies, providing organism-level points of departure and prioritizing compounds for further DNT-IVB and *in vivo* testing within tiered regulatory DNT assessment frameworks.

## 1. Introduction

The assessment of developmental neurotoxicity (DNT) has traditionally relied on rodent models, which are informative but are associated with significant ethical, economic, and practical challenges. The principles of the 3Rs—Replacement, Reduction, and Refinement—have driven the development of New Approach Methodologies (NAMs) to minimize animal use while maintaining scientific rigor in toxicological assessment (Lauwereyns et al., 2024; Sewell et al., 2024). A tiered testing strategy that positions zebrafish behavioral assessment as a first-tier whole-organism screen to prioritize compounds for subsequent mechanistic interrogation via the DNT *in vitro* battery offers an efficient framework for DNT assessment by maximizing organism-level biological context early in the testing sequence while reserving more resource-intensive assays for compounds of greatest concern.

The development of *in vitro* NAMs for DNT assessment represents a significant advancement in mechanistic toxicology. The OECD (Organisation for Economic Co-operation and Development) initiative, beginning in the early 2000s, promoted alternative testing strategies leading to the development of a mechanistically anchored *in vitro* battery capable of efficiently identifying DNT hazard (Sachana et al., 2021). This international coordinated research effort has spanned over 15 years and culminated in the 17 assay DNT *in vitro* battery (DNT-IVB) as described in the OECD initial recommendation document - No. 337 (OECD, 2023a, 2023b). The suite of assays evaluating critical aspects of neurodevelopment, including neurite outgrowth, synaptogenesis, neuronal network formation, and glial function (Bal-Price et al., 2012; Cornet et al., 2017; Fritsche et al., 2018; Behl et al., 2019; OECD, 2023a, 2023b; Blum et al., 2023; Juberg et al., 2023). The DNT-IVB provides targeted assessment of key neurodevelopmental processes, which include neurite outgrowth, synaptogenesis, neuronal network formation, and glial function, across a suite of complementary assays, offering mechanistic insight into which neurodevelopmental pathways are disrupted. However, the battery varies in throughput and commercial availability across its component assays, with some requiring stem cell differentiation or primary cell preparation that limits scalability. Furthermore, *in vitro* approaches cannot fully recapitulate the complex, integrated responses of a developing nervous system within a whole organism, including considerations of absorption, distribution, metabolism, excretion, organ-organ interactions, and system-level integration of neural function, including neurobehavior (Collins et al., 2024; Hughes and Hessel, 2024).

The use of zebrafish (*Danio rerio*) in biomedical research has increased substantially since their first identification as a model organism in the early 1970s (Teame et al., 2019). Over the last 15 years, there has been a threefold increase in research publications utilizing the zebrafish model, with hundreds of laboratories worldwide now employing this system due to its genetic similarity to humans, suitability for high-throughput studies (Truong et al., 2014; Rennekamp and Peterson, 2015; Sakai et al., 2018; Lubin et al., 2021), and potential for reducing reliance on mammalian models (Parichy, 2015; Alzualde et al., 2018; Hamm et al., 2024). This expansion has resulted in broad application of the model for assessing developmental toxicity and DNT, where zebrafish embryos and early larvae provide a whole organism NAM that can complement *in vitro assays* by capturing integrated processes such as absorption, distribution, metabolism, excretion, organ–organ interactions, and neurobehavioral outcomes. Importantly, when used before the onset of free feeding (up to approximately 5 days post fertilization, ~120 hpf), zebrafish are generally not classified as “protected animals” under European legislation (EU Directive 2010/63/EU), and can serve as an alternative to conventional animal experiments while still providing whole organism information on developmental toxicity and neurobehavior, and are increasingly recognized as a NAM that can bridge *in vitro* assays and mammalian *in vivo* testing (European Parliament, 2010; Strähle et al., 2012; MacRae and Peterson, 2015; Cassar et al., 2020).

Zebrafish larvae provide a crucial complementary tier in the NAM framework for DNT assessment. *In vivo* neurobehavioral tests in zebrafish larvae are valuable pre-screening tools for assessing the integrated impact of compounds on the developing nervous system, as they are sensitive to multiple neurophysiological alterations and provide system-level responses that individual *in vitro* assays cannot capture (Collins et al., 2024; Hughes and Hessel, 2024). These assays occur within an intact organism with functional organ systems, yet retain advantages such as a relatively rapid turnaround time, moderate throughput, and lower costs compared to mammalian studies. By positioning zebrafish testing before *in vitro* screening and traditional rodent studies, a tiered strategy can achieve significant reductions in mammalian animal use while maintaining the biological complexity necessary for comprehensive DNT assessment. This approach allows the *in vivo* whole-system zebrafish to rapidly provide organism-level evaluation across large chemical libraries and to prioritize compounds for *in vitro* evaluation for further mechanistic insight and in mammalian models. We hypothesize that the broader molecular and functional applicability domain of the zebrafish model may enable detection of chemicals that fall outside the sensitivity of current *in vitro* assays, though systematic empirical demonstration of this remains an important area for future investigation.

The behavioral Light Dark Transition Test (LDTT) has emerged as a widely used approach for assessing the effects of compounds on zebrafish larval behavior. The LDTT measures locomotor responses to sudden transitions in illumination within standardized light-dark cycles, proving particularly effective for evaluating developmental neurotoxicity following waterborne exposure during critical developmental windows (MacPhail et al., 2009; Irons et al., 2010; Zhang et al., 2017; Morash et al., 2022). The sensitivity of this assay stems from its reliance on integrated neural circuits, including visual processing, motor control, and anxiety-like behavioral modulation, making it responsive to perturbations across multiple neurodevelopmental pathways (Spath et al., 2026). In 2020, an international consortium (authors of this study) comprising government, industry, and academic individuals interested in advancing the zebrafish model collaborated to conduct an interlaboratory study assessing parameters, outcomes, and challenges associated with developing harmonized protocols and data analysis approaches for DNT endpoints in this assay. The collaborators established consensus on standardized elements, including fish strain, temperature, plate format, exposure volume and duration, concentration ranges, and the use of automated behavioral tracking systems.

The present study builds upon this harmonization foundation by demonstrating the practical application of zebrafish LDTT across multiple laboratories using a shared chemical set. Four laboratories collaborated to test 33 compounds (28 suspected developmental neurotoxicants and five putative negative controls) in a blinded study design using agreed-upon standard elements. These compounds had previously been evaluated in the DNT-IVB (Carstens et al., 2025), enabling direct comparison of the information generated by each approach. Additionally, several compounds in this set have been assessed in traditional DNT *in vivo* guideline studies and/or have been used in the development of IATA case studies, including deltamethrin, nicotine, acetamiprid, and several flame retardants, providing an opportunity to evaluate how zebrafish LDTT and DNT-IVB perform. The objectives of this study were to: (1) assess interlaboratory reproducibility of zebrafish LDTT endpoints across multiple testing facilities, (2) evaluate concordance between zebrafish behavioral responses and DNT-IVB outcomes, (3) compare results to available IATA case study data where applicable, and (4) characterize how zebrafish can be positioned within tiered DNT testing strategies. The results refine understanding of this robust zebrafish behavioral model while establishing practical strategies for managing interlaboratory variability and integrating zebrafish data with *in vitro* mechanistic information.

## 2. Methods

### 2.1. Compound selection

Compound selection was guided by recommendations from the OECD DNT Expert group based on prior evaluation of these compounds across multiple *in vitro* assays within the DNT-IVB battery. A set of 33 compounds was selected to support reproducibility testing across laboratories and to enable comparison with the *in vitro* DNT battery (Table 1). The set included known or suspected developmental neurotoxicants (n = 28), and putative DNT-negative compounds (n = 5), to ensure coverage of a broad range of toxicological profiles. The selected developmental neurotoxicants reflected a range of modes of action known to affect the developing brain during critical windows of vulnerability, while DNT-negative compounds were included for contrast.

#### 2.1.1. Rationale

The developmental neurotoxicants (n = 28) represented diverse chemical classes and neurotoxic mechanisms, providing broad coverage of developmental neurotoxicity pathways. These included:

- Organophosphate insecticides (n = 6, e.g., parathion) and carbamate insecticides (n = 2, e.g., aldicarb), which act through acetylcholinesterase inhibition;
- Neonicotinoid insecticides/nicotine (n = 3, e.g., acetamiprid), functioning as nicotinic acetylcholine receptor agonists;
- Pyrethroid insecticides (n = 4, e.g., deltamethrin), which block voltage-gated sodium channels;
- Organochlorine insecticides (n = 3, e.g., dieldrin), associated with disruption of GABAergic and dopaminergic neurotransmission and induction of oxidative stress;
- Flame retardants (n = 9, e.g., 2-ethylhexyl diphenyl phosphate), representing structurally diverse compounds with multiple potential neurotoxic mechanisms
- Herbicides (n = 1, chloramben), an auxin-type growth regulator.

Twelve of the 28 developmental neurotoxicants were identified as having potential solubility challenges due to high logKow values (*>*5), which were anticipated to impact bioavailability in aqueous zebrafish exposures: tris(methylphenyl) phosphate, tert-butylphenyl diphenyl phosphate, dieldrin, kepone, 2-ethylhexyl diphenyl phosphate, heptachlor, deltamethrin, tri-o-cresyl phosphate, permethrin, cypermethrin, 3,3′,5,5′-tetrabromobisphenol A, and isopropylated triphenyl phosphates.

The five putative DNT-negative compounds (glycerol, saccharin, diethylene glycol, ibuprofen, and sodium benzoate) were selected based on the work of Blum et al. (2023) (Blum et al., 2023), representing compounds with low predicted DNT liability based on structural features and lack of activity in the DNT-IVB.

#### 2.1.2. Compounds Studied in OECD IATA DNT Case Studies

Ten of the 33 compounds evaluated in this study have been included in the OECD Integrated Approaches to Testing and Assessment (IATA) case studies for DNT hazard characterization. These consist of seven organophosphate flame retardants (ENV/CBC/MONO(2022)26): tris(2-chloroethyl) phosphate (TCEP), tris(1,3-dichloro−2-propyl) phosphate (TDCIPP), tris(methylphenyl) phosphate (TMPP), tert-butylphenyl diphenyl phosphate (BPDP), 2-ethylhexyl diphenyl phosphate (EHDP), triphenyl phosphates isopropylated (IPP), and 3,3′,5,5′-tetrabromobisphenol A (TBBPA); one pyrethroid insecticide, deltamethrin (ENV/ CBC/MONO(2022)24); and two neonicotinoid insecticides, acetamiprid and nicotine (ENV/CBC/MONO(2022)27). The inclusion of these compounds enables a direct comparison of zebrafish LDTT performance with extensively evaluated DNT case studies, providing an additional data stream to strengthen DNT IATA evaluations and support regulatory applications.

### 2.2. Standardized parameters for zebrafish exposures

AB wild-type adult zebrafish (6–12 months) were housed at 26–28ºC and a pH of 6.5–8 with continuous water filtration on a 14 h light, and 10 h dark photoperiod. The density of housing was 6 fish per L with tanks ranging from 1.0 – 6 L tanks used. Adult zebrafish were fed once or twice daily (depending on the laboratory) with either Sparos Zebrafeed 300 μM (Skretting, USA), mix of Tetramin flakes, or Gemma fish food (Skretting, USA), and live brine shrimp (Artemia). The evening before compound exposures, adult zebrafish were placed in spawning tanks with each sex separated by a vertical divider. The following morning, the dividers were removed, and one hour later, synchronized eggs from different spawning tanks (with at least 3 female contributors) were pooled and placed in 140-mm Petri dishes, 200 embryos per plate, approximately, and covered with E3 medium. E3 medium for zebrafish embryo culture contained 5 mM NaCl, 0.17 mM KCl, 0.4 mM CaCl₂, and 0.16 mM MgSO₄. The embryos were maintained in E3 media + 10 mM HEPES at pH 7.2–7.6 (plus vehicle solution/test compound/positive control solution, depending on the test chamber) throughout the assay until the behavioral test at 5 dpf. Each laboratory prepared E3 media as a 1000X stock solution stored at 4ºC for up to one month. Working solution (1X) with pH adjusted to 7.2–7.6 was prepared fresh weekly.

Only fertilized embryos were used for the studies. Between 1–2 h post fertilization (hpf), embryos were assessed for fertilization rate by sorting and removing unfertilized eggs; only batches with ≥ 70% fertilization were included. After passing QC, fertilized embryos were maintained in E3 medium at 28°C until exposure initiation.

For these studies, eight parameters were standardized: zebrafish strain (AB), chorionated, temperature (28ºC), plate format (96-well square well plates), exposure volume (500 μL), exposure duration (6–120 hpf), 8 test concentrations (7 + vehicle control), and an automated behavioral tracking system (Viewpoint LifeSciences Zebrabox or Noldus DanioVision). The LDTT light protocol was consistent across all four laboratories. Detailed information of other parameters can be found in Supplemental Table 1.

### 2.3. Compound preparation

All 33 compounds in this study were procured from a single source and prepared as 20 mM stock solutions in 100% dimethyl sulfoxide (DMSO), then stored at 4°C. Compounds were blinded and were provided by the Division of Translational Toxicology contractor (MRI Global, Kansas City, MO, USA) to minimize variability in chemical purity across laboratories. The same stock solution lot and DMSO vehicle were distributed to all four testing laboratories, designated as Lab A, Lab B, Lab C, and Lab D. On the day of exposures, working solutions were made by diluting the stock solution in E3 medium and HEPES to achieve the desired test concentrations. 2X working solutions of the desired final concentrations were prepared at 6 hpf.

### 2.4. Compound exposure

For compound exposures, 96-well square well plates (Whatman) were prefilled with 250 μL of E3 medium + HEPES, and single chorionated embryos were placed in each well at 6 hpf. An equal volume (250 μL) of 2X compound working solution was added to achieve final test concentrations, mixed either with an automated pipette or via orbital shaker for 10 s, then sealed with breathable rayon film (VWR North American Cat# 60941–084/ European article # 391–1261). Plates were immediately placed in a 28 ± 1ºC incubator with a 14 h light:10 h dark cycle until 120 hpf. The final DMSO concentration in all wells was 0.4%, with a total volume of 500 μL.

Because compounds were provided to laboratories in a blinded manner, one designated laboratory conducted a preliminary concentration range-finding study to identify appropriate test concentration ranges for each of the 33 compounds. All laboratories were instructed to test at least one plate using this recommended concentration range, but were permitted to adjust or expand concentrations based on laboratory-specific observations (e.g., precipitation, mortality). Additionally, because available resources differed across laboratories, some laboratories tested multiple replicate plates per chemical.

At 120 hpf (±2 h), larvae were transferred to an automated tracking instruments: DanioVision (Noldus, Netherlands), or ZebraBox (Viewpoint Lifesciences, France) for LDTT assessment. All behavioral testing was initiated 4–8 h after the incubator lights turned on. The LDTT protocol consisted of an initial acclimation period (5 min light, 5 min dark) followed by standardized testing phases detailed in Table 2. The locomotor activity of the larvae was recorded for 50 min (tracking time) after an initial acclimation period of 10 min (5 min light (ON) and 5 min dark (OFF), where the ON light intensity was between 850 and 1000 lux. The testing light protocol during the tracking time was 10 min ON + 10 min OFF + 10 min ON + 20 min OFF with a light switch from 100% to 0% and 0% to 100% (no slope change). The primary DNT-specific endpoint measured was the total distance (millimeters) moved per minute, as determined by automated tracking systems listed above. After the LDTT assay, each larva was inspected for morphological abnormalities or the absence of an inflated swim bladder, and recorded to be excluded from the DNT data analysis.

### 2.5. Assay Endpoints

Two primary assay endpoints were assessed: developmental toxicity (DT) and developmental neurotoxicity (DNT). DT was evaluated at 120 hpf (immediately following LDTT assessment) using two binary indicators: (1) abnormal morphology including bent body axis; edema of the brain, pericardium, and yolk sac; short or lack of a jaw/snout and caudal fin deformities) or mortality (absence of a heartbeat), and (2) absence of an inflated swim bladder. Larvae exhibiting either of the DT indicators were excluded from DNT behavioral analysis but retained for DT hazard assessment.

DNT was assessed through locomotor activity measured during the four testing phases of the LDTT assay (L1, D1, L2, D2; Table 2). For each larva, total distance moved (mm) was recorded in one-minute intervals throughout each phase.

### 2.6. Data analysis

Data from all four laboratories were collected using a standardized format and compiled in an SQLite database to facilitate harmonized data management and analysis of zebrafish LDTT data. The analysis pipeline consisted of quality control filtering, analytical endpoint calculation, and benchmark concentration modeling.

#### 2.6.1. Analytical endpoint calculation

For each embryo, two types of data were recorded: (1) malformation status, based on two binary indicators: abnormal morphology/mortality and absence of swim bladder inflation, and (2) behavioral activity, measured as total distance moved per minute during each of the four LDTT testing phases. A single developmental toxicity (DT) endpoint (n = 1) was calculated for each exposure concentration as the percentage of embryos exhibiting either abnormal morphology/mortality or a lack of an inflated swim bladder.

Developmental neurotoxicity (DNT) assessment consisted of 11 behavioral endpoints:

- Movement Similarity endpoint (n = 1), which quantifies how closely each treated larva’s movement pattern aligns with that of untreated larvae across the complete 50-minute assay using Spearman rank correlation;
- Distance Change endpoints (n = 4), which quantify general perturbation in activity for each LDTT phase relative to the median movement of vehicle control larvae;
- Distance Shift endpoints (n = 6): which quantifies hyperactivity (4 endpoints, one per phase) and hypoactivity during dark phases (2 endpoints, D1 and D2 only) relative to the median movement of vehicle control larvae.

Behavioral endpoints were calculated only for larvae that were not malformed and had an inflated swim bladder. In Table 2, we mapped each calculated endpoint to its corresponding phase and definition. Power for these endpoints was evaluated in our companion study (Konrad KS et al., 2026). In general, 8–32 embryos per dose group are needed to achieve 80% power to detect a 20% effect size, depending on the endpoint, phase, and test. The number of embryos was estimated in the power calculation using responses from embryos treated with vehicle control only. However, the resulting sample size estimates are applicable to dosed studies(Jeffers et al., 2024).

#### 2.6.2. Quality control

**Fig. 1.**
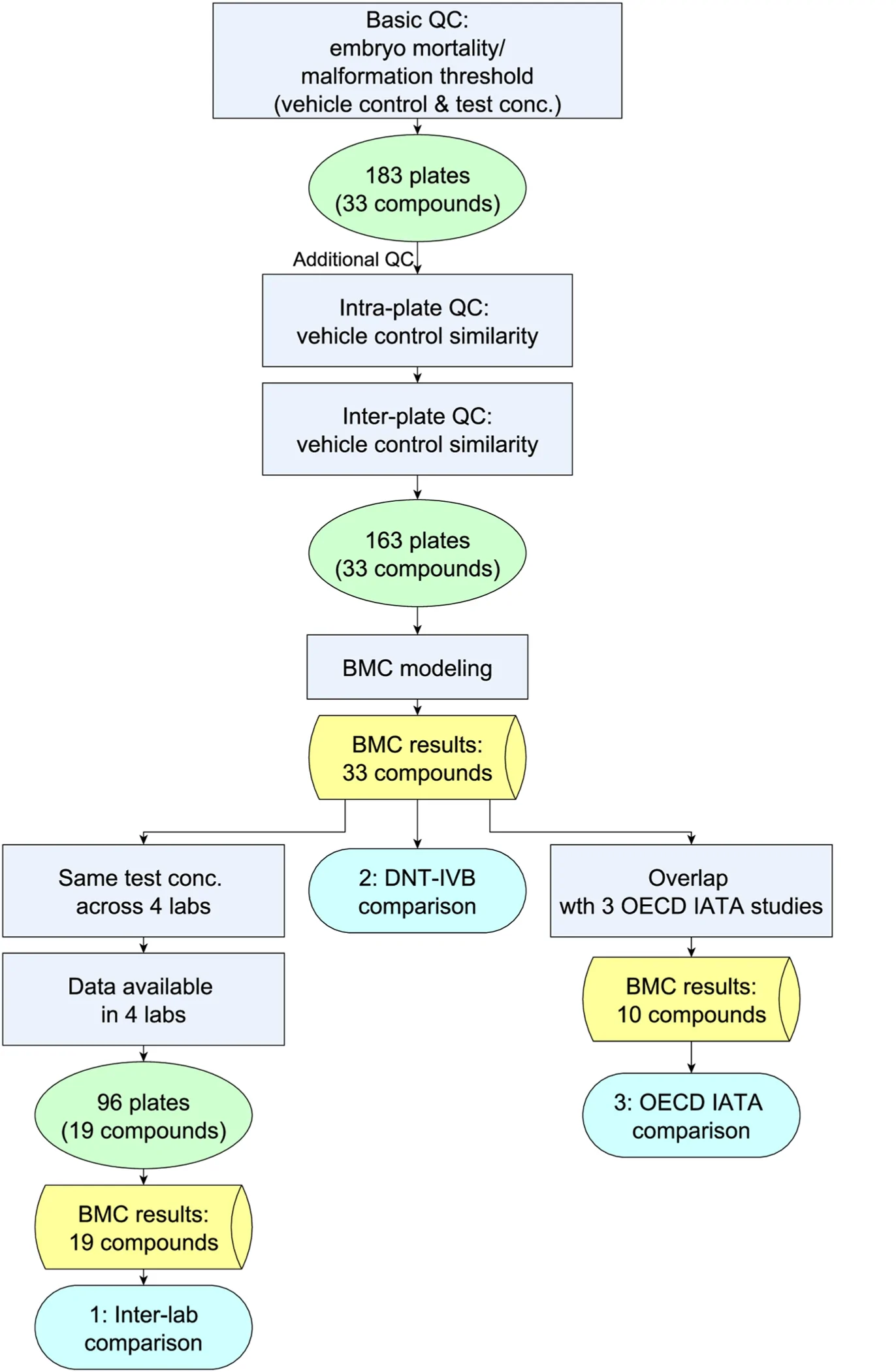
Overview of workflow for Quality Control (QC) and analysis applied to the multi-laboratory zebrafish LDTT dataset. The workflow starts with a basic QC based on embryo mortality/malformation threshold, initially including 183 plates representing 33 compounds. Secondary QC steps involve intra-plate and inter-plate vehicle control pattern similarity assessments, refining the dataset to 163 plates with 33 compounds. This refined dataset is subjected to BMC modeling. BMC outputs supported three downstream analyses: (1) inter-laboratory comparison of 19 compounds tested at harmonized concentrations across laboratories; (2) DNT-IVB comparison of 33 compounds, and (3) comparison of 10 compounds overlapping with OECD IATA DNT case-study datasets.

A three-step quality control process was implemented in each laboratory to ensure data reliability by excluding plates with suboptimal conditions that could compromise concentration-response detection (Fig. 1).

##### 2.6.2.1. Basic Quality Control.

Plates were excluded if excessive mortality or malformation (among either vehicle control or compound- treated embryos) resulted in insufficient viable larvae for robust analysis. Each 96-well plate tested a single compound at seven concentrations plus vehicle control (DMSO), with 12 replicate wells per concentration (n = 12 larvae per concentration). Plates were required to meet two criteria for inclusion: (1) at least 75% of vehicle control wells contained viable larvae eligible for analysis (≥9 of 12 wells), and (2) at least four of seven test concentrations retained ≥ 75% viable larvae (≥9 of 12 wells per concentration).

##### 2.6.2.2. Intra-plate vehicle control QC.

Plates with inconsistent movement patterns among vehicle control larvae were excluded. The Movement Similarity endpoint was used to measure pairwise similarity among untreated larvae on the same plate using Spearman’s rank correlation. The median of these similarity scores from untreated larvae quantified the overall movement-pattern similarity within the plate, with higher values indicating greater consistency (where 1 denotes perfect similarity and 0 denotes no similarity). A threshold of 0.4 was used to exclude plates with low consistency in movement patterns among vehicle control wells. This cutoff was established based on the response distribution observed within each laboratory.

##### 2.6.2.3. Inter-plate vehicle control QC.

Plates with vehicle control movement patterns that were statistical outliers relative to other plates were excluded. For each plate, a reference movement pattern was calculated using the median distance moved per minute by DMSO exposed larvae. To assess consistency of movement patterns, principal component analysis (PCA) was performed using the R package Facto-MineR. The first two principal components were employed to construct an ellipse representing the 95% confidence region using a bivariate normal distribution. Plates with reference movement patterns outside this ellipse were considered statistical outliers and excluded from further analysis.

#### 2.6.3. Benchmark concentration modeling

Benchmark concentration (BMC) modeling was performed to estimate the concentration at which each compound produced a benchmark response (BMR) for each endpoint. Modeling used the Curvep approach in the Rcurvep package (https://cran.r-project.org/package=Rcurvep) (Hsieh et al., 2018; Carstens et al., 2025). Curvep uses a nonparametric noise-filtering algorithm that identifies the minimum set of corrections needed to transform the data into a monotonic concentration-response curve, based on empirically defined parameters(Sedykh et al., 2011; Sedykh, 2016).

Movement Similarity responses were plate-normalized prior to modeling using the equation: Response = (V_compound_/V_vehicle control_) * 100 – 100, where V_compound_ denotes the raw response from the well administered with a compound and V_vehicle control_ denotes the median of endpoint values from vehicle control-only wells. Distance Change and Distance Shift responses were used directly without normalization.

For Movement Similarity endpoints, the BMR was determined using Curvep’s variance-based optimization approach, which identifies the lowest response threshold that sufficiently reduces variance in potency estimation. The search range was 5–95% with 100 bootstrapped samples using functions in the Rcurvep package. For the Distance Change and Distance Shift endpoints, BMR was set to the median value plus three median absolute deviations (MADs) of the responses from vehicle control across all plates.

Once BMR was established, 1000 concentration-response curves were generated for each compound by bootstrapping response data at individual concentrations (maintaining 1:1 concentration-response relationships). These curves were then processed using the predetermined BMR value. BMC for each simulated curve was calculated as the concentration corresponding to the BMR. For curves deemed inactive at the given BMR, the highest tested concentration was assigned as the BMC.

An activity confidence score was calculated as the fraction of curves identified as exhibiting a monotonic response after noise filtering. A confidence score greater than 0.5 (indicating that *>*50% of bootstrapped curves exhibited monotonic concentration-response relationships) was used as the threshold for a positive hit call. The 95% confidence interval for BMC was estimated using the quantile approach, with the 0.025 and 0.975 quantiles as the lower and upper bounds, respectively.

#### 2.6.4. BMC data summary and selection

Benchmark concentration modeling was conducted for 12 analytical endpoints, comprising one malformation DT endpoint and 11 behavioral DNT endpoints. For inter-laboratory comparisons, a standardized summary procedure was employed to select representative BMC values from each plate. A single behavioral BMC per plate was selected from endpoints with positive hit calls (confidence score *>*0.5, as defined in Section 2.6.3), prioritizing those with the highest active confidence score. Ties were resolved by selecting the endpoint with the lowest BMC value. When multiple plates per compound were tested within a laboratory, the plate with the highest number of active endpoints was selected. Subsequent ties were resolved by prioritizing plates with the highest active confidence score, then selecting the plate with the lowest BMC value. The malformation endpoint BMC from the chosen plate was included in all comparative analyses.

#### 2.6.5. DNT In Vitro battery data compilation

For comparative analysis with zebrafish LDTT, activity data for the majority of the DNT-IVB (missing 4 assays) were retrieved using the R package *cdxR* from the U.S. EPA’s Chemical Dashboard (https://comptox.epa.gov/dashboard, invitroDB v4.2, downloaded November 15, 2025). The DNT-IVB comprises 13 unique assays: CCTE_Shafer_MEA_dev (microelectrode array); CCTE_Mundy_HCI_hNP1_Casp3_7, CCTE_Mun dy_HCI_hNP1_CellTiter, CCTE_Mundy_HCI_hNP1_Pro (neural progenitor assays); CCTE_Mundy_HCI_iCellGluta_NOG (glutamatergic neuron assay); IUF_NPC1, IUF_NPC2–5 (neural progenitor cell assays); UKN2_HCS_IMR90 (neurite outgrowth in fibroblasts); UKN4_HC-S_LUHMES, UKN5_HCS_SBAD2 (LUHMES dopaminergic neuron assays); CCTE_Mundy_HCI_Cortical_NOG, CCTE_Mundy_HCI_Cortical_Synap&Neurite_Matur, CCTE_Mundy_HCI_hN2_NOG (cortical neuron assays). These assays collectively encompass 64 analysis endpoints (identified as aeid in invitroDB).

Data were batch-downloaded for all 64 aeids, retaining only the 33 compounds that were also screened in the zebrafish LDTT. It should be noted that assay coverage varied across the 33 compounds, as not all compounds were tested in all DNT-IVB assays. Within DNT-IVB, compounds with hit calls (hitc *>* 0.9) were considered active. For each compound, the most potent (lowest) ACC value across all active DNT-IVB endpoints was selected for comparison with the most potent BMC value across laboratories from zebrafish LDTT. ACC values were compared directly with zebrafish BMC values, as both metrics represent the estimated concentration at which a compound produces a predefined biologically relevant response threshold (Carstens et al., 2025).

### 2.7. Data availability

The supplemental data provide plate-level, endpoint-level, and potency-level datasets as well as movement-over-time plots for all the plates. Only data from plates that passed all quality-control (QC) steps are included. The data can be downloaded from the CEBS page (https://doi.org/10.22427/DATA-500–107-004–000-7).

## 3. Results

Results are presented in five sections following the analytical framework depicted in Fig. 1. First, we describe the implementation of quality control procedures and their impact on plate retention across laboratories (Section 3.1). We then evaluate inter-laboratory reproducibility at two levels: (1) agreement in compound activity classifications (DT and DNT hit calls) (Sections 3.2), and (2) potency comparisons using benchmark concentration (BMC) estimates (Section 3.3). Next, we compare zebrafish LDTT and DNT-IVB results to assess inter-method concordance, sensitivity to known DNT-positive compounds, and specificity for DNT-negative compounds (Section 3.4). Finally, we contextualize these findings within OECD IATA DNT case studies through detailed chemical-specific evaluations of compounds with existing regulatory assessments: deltamethrin, nicotine, acetamiprid, and seven flame retardants (Section 3.5). Together, these analyses provide a comprehensive evaluation of zebrafish LDTT performance on identifying compounds with DNT evidence, inter-laboratory reproducibility, and integration potential within tiered DNT assessment frameworks.

### 3.1. Quality control implementation

A total of 203 plates were tested across the four laboratories. Following basic quality control (Section 2.6.2.1), 183 plates (90%) met minimum viability criteria and advanced to secondary quality control assessment. After basic QC, the number of plates differed across laboratories (Lab-A: 37, Lab-B: 59, Lab-C: 48, Lab-D: 39, n = 183). Then we applied a two-step procedure to further enhance data reliability by identifying and removing plates with inconsistent movement patterns among vehicle control (DMSO) larvae, either within plates (intra-plate QC) or across plates (inter-plate QC). The plate information with QC flags is available in the Supplemental Table 2.

**Fig. 2.**
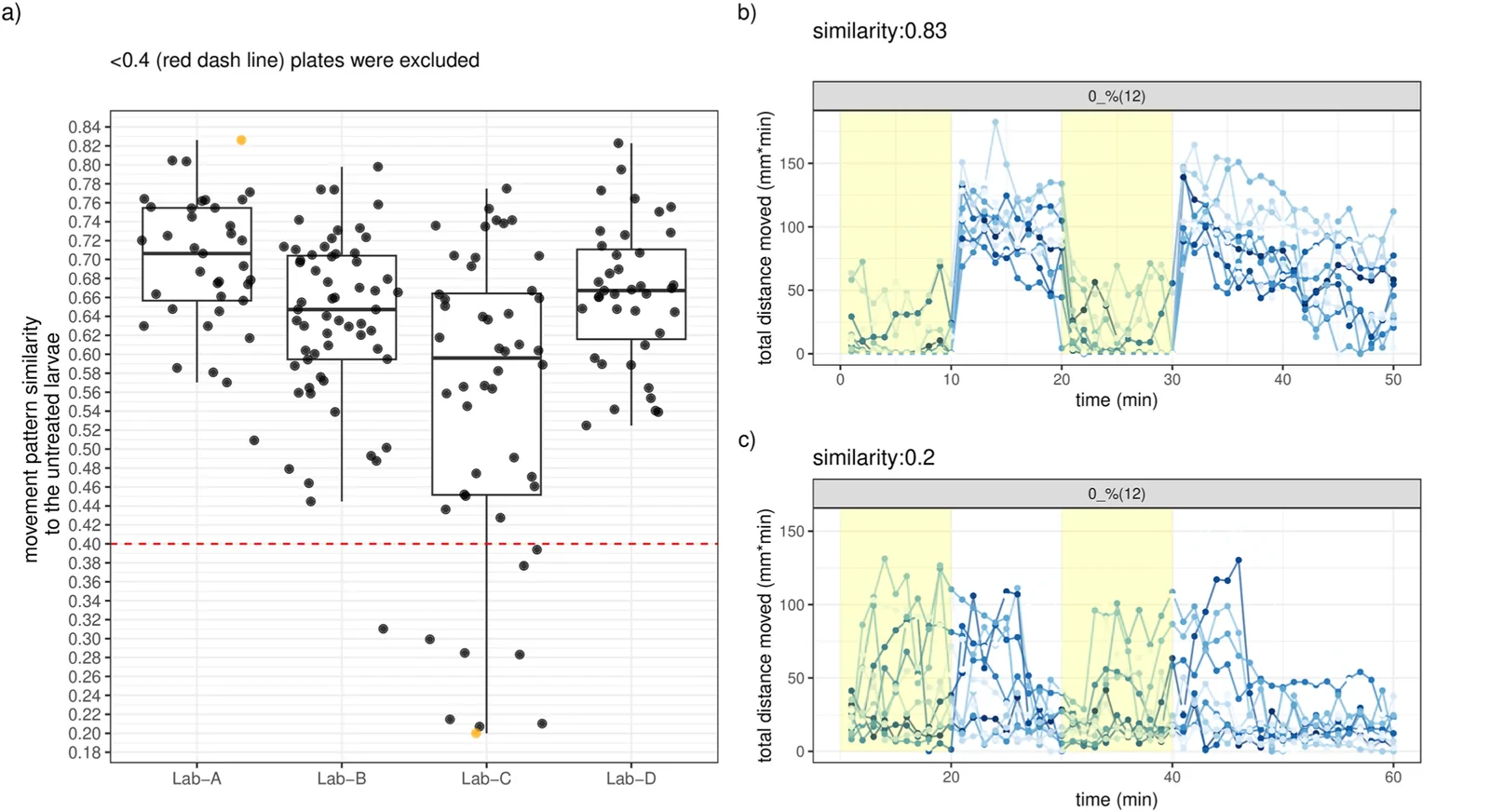
Intra-Plate Quality Control (QC) Analysis of Larval Movement Pattern Similarity. (a) Box plots showing the movement pattern similarity among untreated larvae within individual plates, evaluated across four laboratories (Lab-A to Lab-D). Each dot represents a plate’s intra-plate movement pattern similarity score. A red dashed line at the 0.4 mark indicates the similarity threshold—plates falling below this line are excluded from further analysis. Two orange-highlighted dots correspond to the example plates illustrated in panels (b) and (c). (b) Movement-over-time plot displaying the total distance moved (mm/min) of each untreated larvae within a plate, exhibits high intra-plate similarity (0.83), suggesting consistent movement patterns. (c) A similar plot demonstrating a plate with low intra- plate similarity (0.2), indicative of variable movement patterns. Yellow-shaded sections highlight periods of light phases.

The first QC step evaluated the similarity of movement patterns among vehicle control larvae within each plate using the Movement Similarity metric (Section 2.6.2.2). Plates with median similarity scores *<* 0.4 were excluded. Substantial variation in intra-plate movement pattern similarity was observed both within and between laboratories (Fig. 2a). Lab-A showed the highest and most consistent similarity values, with median similarity *>* 0.7. Lab-B and Lab-D exhibited intermediate consistency, with interquartile ranges generally between 0.6 and 0.7. In contrast, Lab-C displayed the greatest variability, including four low-similarity plates with values near 0.2, far below the 0.4 threshold. This QC step effectively distinguished plates with stable baseline movement patterns from those exhibiting irregular larval movement profiles, as illustrated by representative high- and low-similarity examples in Figs. 2b and 2c. In total, 10 plates were excluded based on this criterion, including nine plates from Lab-C and one plate from Lab-B.

**Fig. 3.**
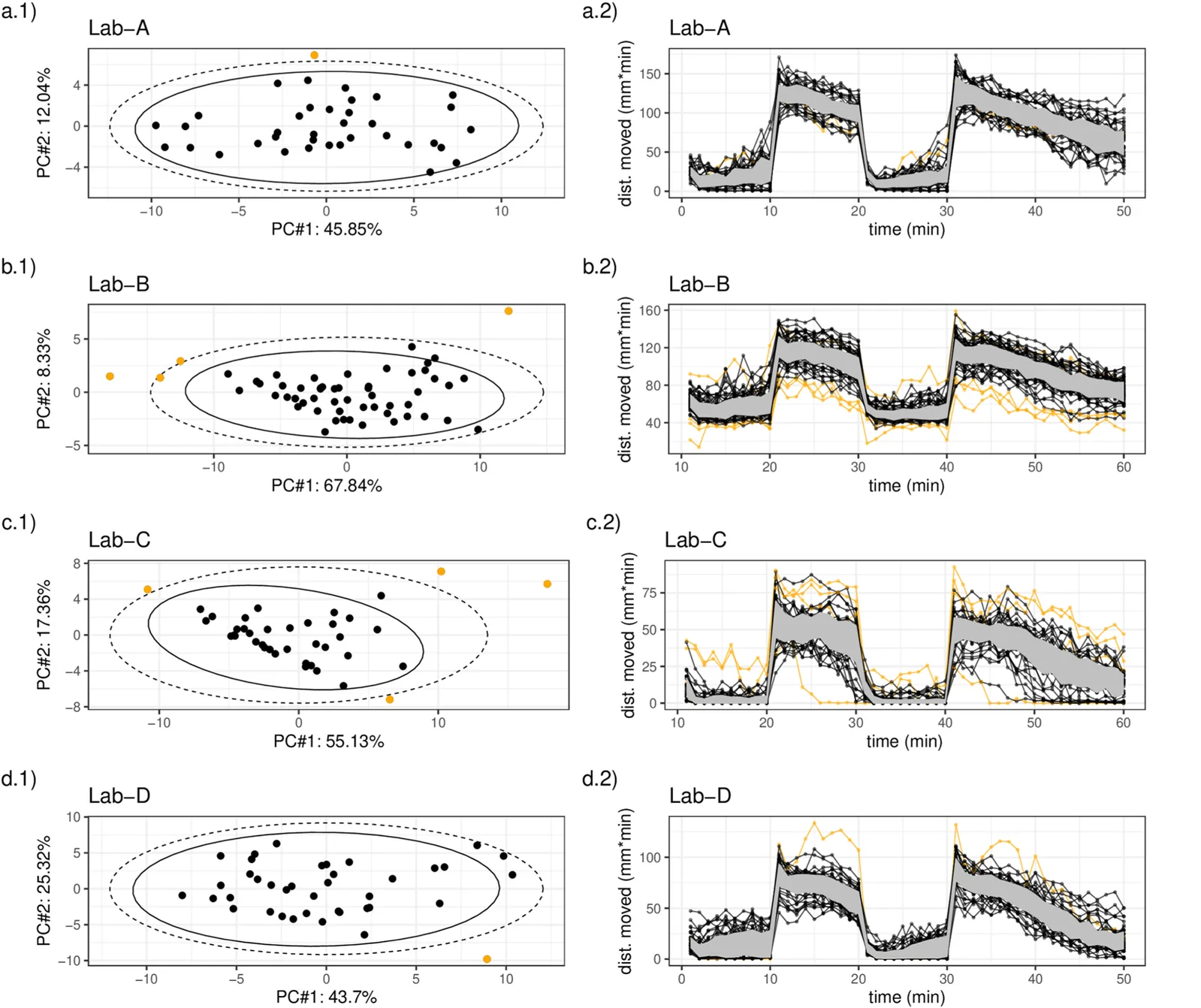
Inter-Plate Quality Control (QC) Analysis of Larval Movement Patterns Across Laboratories. Panels (a.1)-(d.1) display the principal component analysis (PCA) results for each laboratory (Lab-A to Lab-D), based on the reference movement patterns of plates. The x and y axes show the coordinates of the first two principal components (PCs), with percentages indicating the variance each component explains. Ellipses represent the 95% confidence intervals for plate distributions, with solid lines indicating the Studenťs t-distribution and dashed lines representing the normal distribution. Orange-highlighted dots mark plates identified as outliers in this analysis. Panels (a.2)-(d.2) depict movement-over-time plots showing the reference movement patterns of all plates within each laboratory. These plots illustrate the total distance moved per minute, with shaded areas representing the interquartile range (IQR) for movement at each time point. The orange line in these panels corresponds to the orange-highlighted dots in Panels (a.1)-(d.1).

The second QC step used principal component analysis (PCA) to identify statistical outliers based on vehicle control movement patterns within each laboratory (Section 2.6.2.3, Fig. 3, panels a.1-d.1). Across laboratories, the sum of PC1 and PC2 explained 57.9–76.2% of the total variance. Each laboratory exhibited a characteristic clustering pattern. Lab-A and Lab-D showed relatively tight clusters in PC space, with 95% confidence ellipses (t-distribution, solid line; normal distribution, dashed line) closely aligned, indicating consistent baseline movement patterns with minimal plate-to-plate deviation. In contrast, Lab-B and Lab-C demonstrated broader dispersion, with several plates falling outside the 95% normal-distribution ellipse. Corresponding movement traces (Fig. 3, panels a.2-d.2) confirmed that excluded plates (orange) displayed atypical baseline or phase-specific activity relative to each laboratory’s interquartile range. These deviations were most pronounced in Lab-B, Lab-C, and Lab-D, whereas Lab-A’s single outlier exhibited only modest divergence. An additional 10 plates were excluded based on this inter-plate QC criterion: 1 from Lab-A, 4 from Lab-B, 4 from Lab-C, and 1 from Lab-D.

Combined, these secondary QC steps removed 20 plates (10.9% of plates passing basic QC), reducing from 183 to 163 for BMC modeling (Fig. 1). Final plate distributions were: Lab-A (n = 36), Lab-B (n = 54), Lab-C (n = 35), and Lab-D (n = 38). QC exclusion rates varied by laboratory, with Lab-C showing the highest rate (27%, 13/48 plates) and Lab-A the lowest (3%, 1/37 plates). These 163 QC-passed plates were included for subsequent analyses.

### 3.2. Inter-Laboratory Comparison (Activity Calls)

A total of 163 plates passed all quality control steps and advanced to BMC modeling. Complete BMC results for all endpoints and compounds are provided in Supplemental Data. For inter-laboratory comparison, compounds were required to have at least one plate per laboratory testing the same concentration range. This criterion was met by 19 of 33 compounds. These 19 compounds included: 2 organophosphate insecticides (of 6 tested), 1 carbamate insecticide (of 2), 1 neonicotinoid insecticide (of 3), 2 pyrethroid insecticides (of 4), 2 organochlorine insecticides (of 3), 5 flame retardants (of 9), 1 herbicide (of 1), and all 5 putative DNT-negative compounds. Among these groups, only the herbicide and the DNT-negative compounds were fully represented relative to the original test set. This 19-compound subset (96 plates total) was used for all inter-laboratory comparisons of DT and DNT activity and potency.

**Fig. 4.**
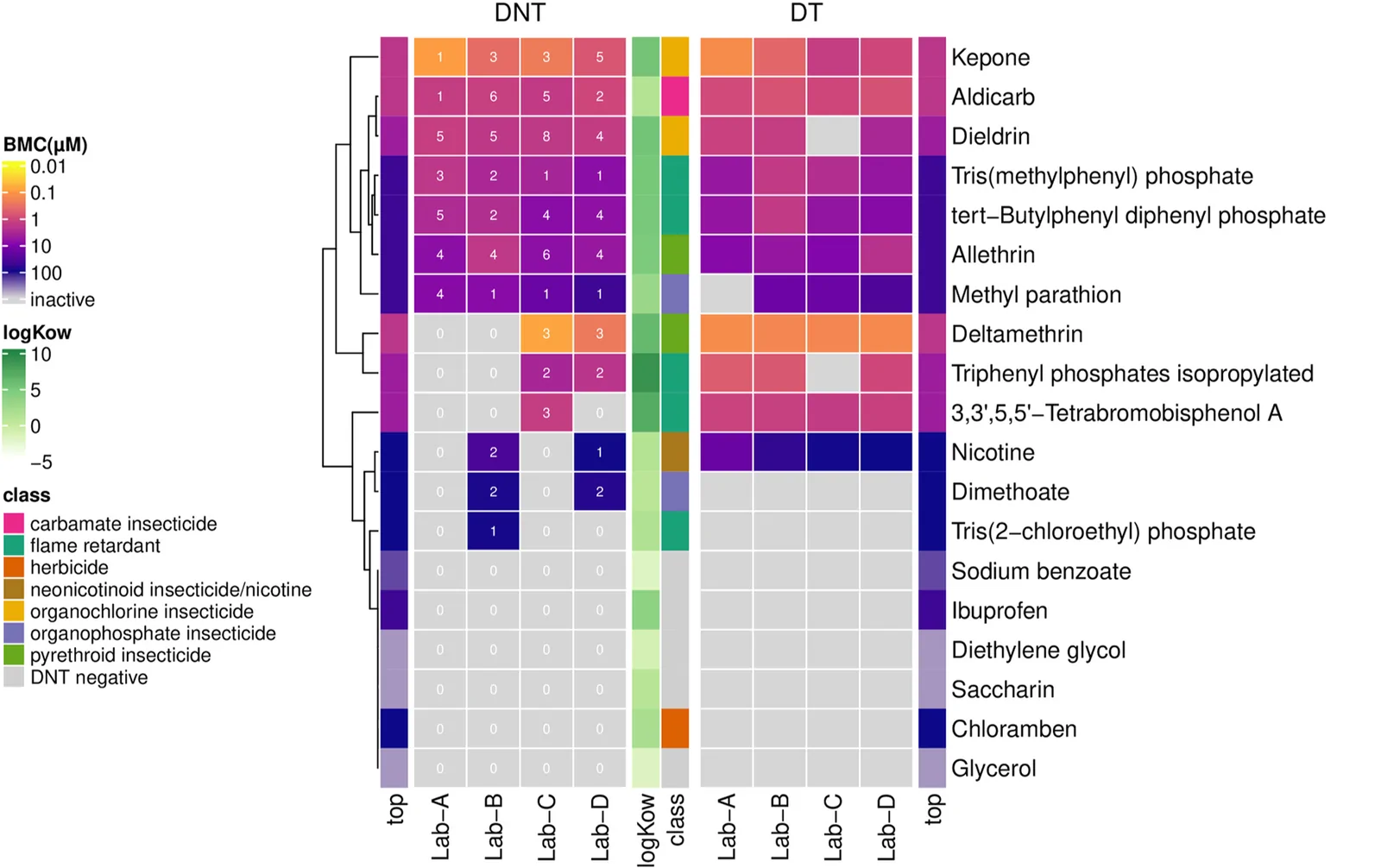
Cross-Laboratory Comparison of DNT and DT Activities of 19 Compounds. Heatmap representing the DNT and DT activities of 19 compounds (rows) across four laboratories (columns: Lab-A to Lab-D). The color gradient within each cell illustrates benchmark concentration (BMC) values in μM, ranging from 0.01 μM (lighter shades) to 100 μM (darker shades). Gray cells indicate inactive effects. Compounds have been hierarchically clustered based on their DNT activities to highlight patterns and similarities. An additional column, labeled as ‘top’ (duplicated on both sides for easy comparison), displays the highest concentration used in the assays for each compound. The use category of each compound and their associated logKow values are presented in separate columns. Within the DNT cells, the numbers denote the count of active endpoints identified.

Within this dataset, Lab-A and Lab-D each contributed one plate per compound. In contrast, Lab-B contributed multiple plates (2 plates) for 14 of 19 compounds, and Lab-C contributed multiple plates (2–5 plates) for 4 of the 5 DNT-negative compounds. BMC values for each compound–laboratory combination were summarized following the plate-selection procedure described in Section 2.6.4. DT and DNT BMC results were visualized using a heatmap annotated with the highest tested concentration, logKow, and chemical use class to facilitate interpretation of potency patterns and chemical properties (Fig. 4). The background data are available in the Supplemental Table 2.

Overall, DT activities were more concordant across laboratories than DNT activities. Pairwise concordance across the four laboratories ranged from 84.2% to 100% for DT, compared with 68% to 84% for DNT. Six compounds were consistently inactive for both DT and DNT in all four laboratories; these included all five putative DNT-negative compounds and the herbicide chloramben (Supplemental Table 3). The remaining 13 compounds—all classified as known or suspected developmental neurotoxicants—showed varying degrees of activity. Of these, 11 exhibited concordant DT activity (active in ≥3 of 4 laboratories), while DNT activity patterns were more variable.

To explore potential drivers of DNT variability, we examined logKow values and their relationship with the highest tested concentrations. Among six compounds tested at concentrations below 10 μM, three highly lipophilic compounds—deltamethrin (logKow 6.2), triphenyl phosphates isopropylated (IPP; logKow 9.07), and 3,3′,5,5′-tetrabromobisphenol A (TBBPA; logKow 7.2)—exhibited discordant DNT activity but concordant, active DT responses. All three compounds had logKow *>* 6, the top 3 highest values in the dataset. Among nine compounds tested at 10–100 μM, three—nicotine, dimethoate, and tris(2-chloroethyl) phosphate (TCEP)—also showed discordant DNT activity. These compounds exhibited either weak yet concordant DT responses (nicotine) or uniformly inactive DT responses (dimethoate and TCEP). All three had lower logKow values (0.78–1.44). In contrast, four compounds tested at concentrations *>* 100 μM showed concordant inactivity for both DNT and DT across all laboratories. All four were hydrophilic (logKow − 1.92–0.91).

We further evaluated whether the number of active DNT endpoints per compound contributed to call confidence or inter-laboratory agreement (white numbers in Fig. 4). No clear relationship was evident; many compounds (e.g., tris(methylphenyl) phosphate, TMPP) with fully concordant DNT calls exhibited activity in only one endpoint per laboratory.

For the remaining 14 compounds that were not included in the four-laboratory inter-laboratory comparison, 11 had at least one QC-passing plate tested at the same concentration range in three laboratories, allowing an additional three-laboratory comparison. Specifically, one compound was comparable across Labs A–B–C, four compounds across Labs A–B–D, and six compounds across Labs A–C–D. For these 11 compounds, all except acetamiprid and heptachlor had two plates tested by Lab-B; the remaining laboratory–compound combinations were represented by one plate. BMC values were summarized using the same plate-selection procedure applied to the four-laboratory comparison in Fig. 4. The resulting DT and DNT activity patterns are shown in Supplemental Figure 2 and the background data are included in Supplemental Table 5.

Because this analysis included fewer compounds and different laboratory combinations, the results were interpreted descriptively rather than as a formal concordance analysis. The three-laboratory comparison recovered additional activity information for compounds that were otherwise excluded from the four-laboratory dataset. For example, cypermethrin, evaluated across Labs A–C–D, showed discordant DNT activity while DT activity was more consistently detected. This pattern is notable because cypermethrin is also a highly lipophilic pyrethroid insecticide (logKow *>*6) and is structurally most similar to deltamethrin, which showed discordant DNT activity but concordant DT activity in the four-laboratory comparison. In contrast, the other two pyrethroid insecticides, permethrin and allethrin, showed more concordant DNT and DT responses. Trichlorfon, evaluated across Labs A–B–D, showed a strong DNT effect in Lab-A but was inactive in the other two laboratories, suggesting a laboratory-specific signal that warrants further investigation. Overall, this supplemental three-laboratory analysis increased coverage of comparison from 19 to 30 of the 33 compounds and provided additional context for compounds excluded from the primary four-laboratory comparison, while avoiding overinterpretation of trends from a smaller and compositionally different subset.

### 3.3. Inter-Laboratory Comparison (Potency)

**Fig. 5.**
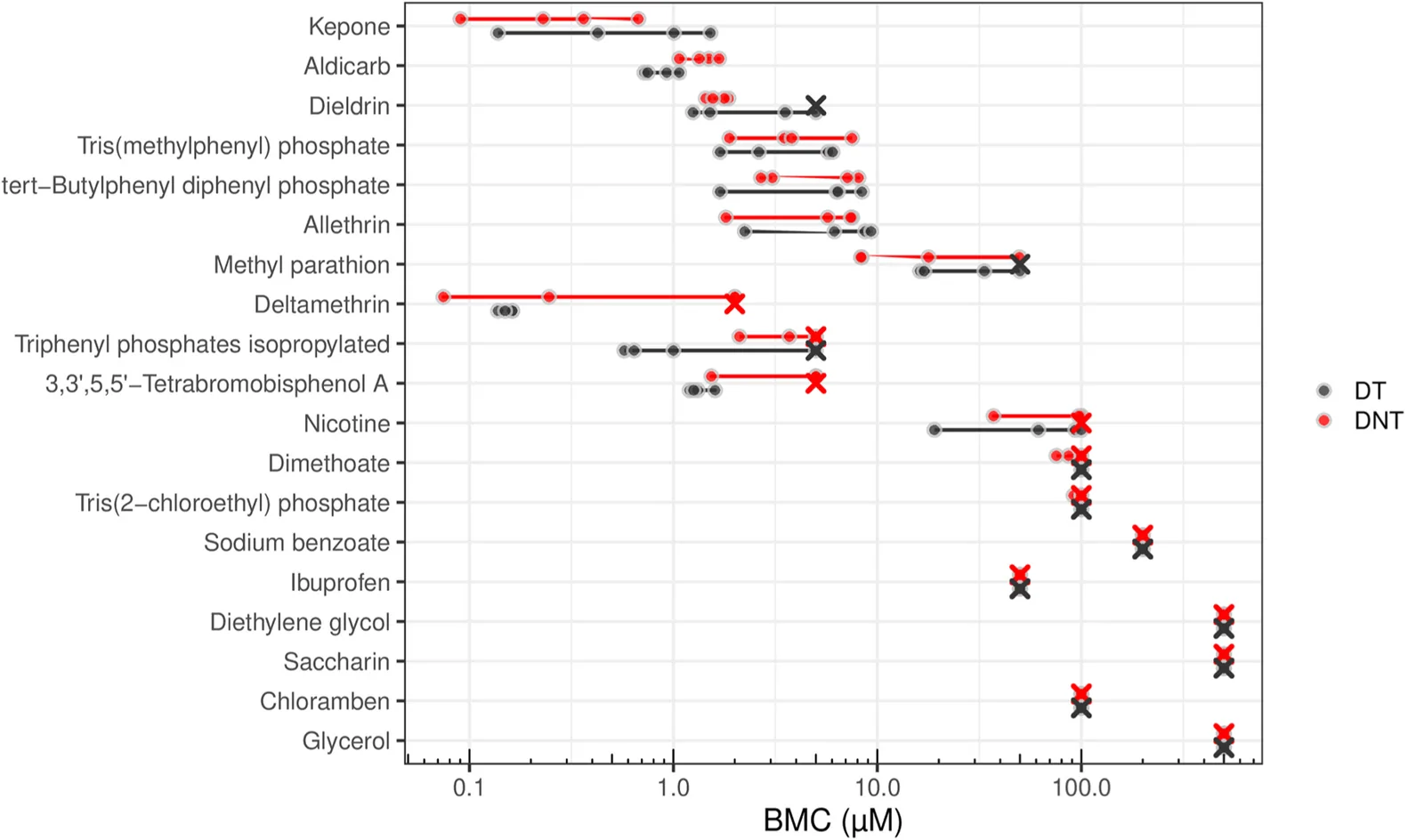
Zebrafish DNT and DT Activity Variation Analysis Across Laboratories. This plot presents the benchmark concentrations (BMC) for the effects of 19 compounds in zebrafish, illustrating DNT (red markers) and DT (black markers) effect, as depicted in the heatmap in Fig. 4. The order of the compounds follows the sequence used in Fig. 4. Each red/black line can feature up to four dots, representing the DNT/DT activities evaluated in four different laboratories. An “X” on top of a dot indicates that the compound was inactive in that specific lab; when inactive, the highest tested concentration is reported as the BMC for plotting purpose.

To evaluate inter-laboratory potency concordance, DNT and DT BMC values for each compound were visualized using dot plots (Fig. 5). After excluding the six compounds inactive in all laboratories (the five putative DNT-negative controls and chloramben) and substituting the highest tested concentration as the BMC for inactive calls, the mean inter-laboratory BMC variation was 0.51 log_10_ units (~3.2-fold) for DNT and 0.55 log_10_ units (~3.5-fold) for DT.

Inter-laboratory potency variability ranged widely across compounds: the largest DNT potency spread was observed for deltamethrin (26.9-fold across laboratories), while kepone showed the largest DT spread (11.0-fold). Conversely, deltamethrin exhibited the most consistent DT potency across laboratories (1.2-fold range among DT- active laboratories), and dieldrin showed similarly consistent DNT potency (1.3-fold). Notably, deltamethrin exhibited both the largest DNT potency spread (26.9-fold) and the most consistent DT potency (1.2-fold).

We also observed that most compounds showed considerable overlap between their DNT and DT BMC values (Figs. 4 and 5). To quantify this relationship, we calculated the log_10_ difference between DNT and DT BMCs for each compound-laboratory combination where both endpoints were active. Across all compounds and laboratories, the median log_10_ difference was close to zero, indicating that DNT and DT effects occurred at similar concentration ranges for more than half of the compounds. Laboratory-specific distributions of log_10_ differences are shown in Supplemental Figure 1.

A similar dot-plot visualization was generated for the three-laboratory comparison dataset (Supplemental Figure 3). After excluding acetamiprid, the only compound inactive in all three laboratories for both endpoints, and assigning the highest tested concentration as the BMC for inactive calls, the mean inter-laboratory BMC variation was 0.31 log_10_ units (~2.1-fold) for DNT and 0.42 log_10_ units (~2.7-fold) for DT. These values were slightly lower than those observed in the four-laboratory comparison, although direct comparison should be interpreted with caution because the three-laboratory dataset included a smaller, compositionally different set of compounds. Heptachlor and permethrin showed the largest potency ranges in this subset. For DT, inter-laboratory variation exceeded 0.9 log_10_ units for both chemicals (heptachlor: 1.02 log_10_, ~10.5-fold; permethrin: 0.95 log_10_, ~8.9-fold). Overall, the three-laboratory potency analysis provided additional context for compounds excluded from the primary four-laboratory comparison and did not suggest substantially greater potency variability than the main dataset.

### 3.4. Comparison with DNT-IVB

**Fig. 6.**
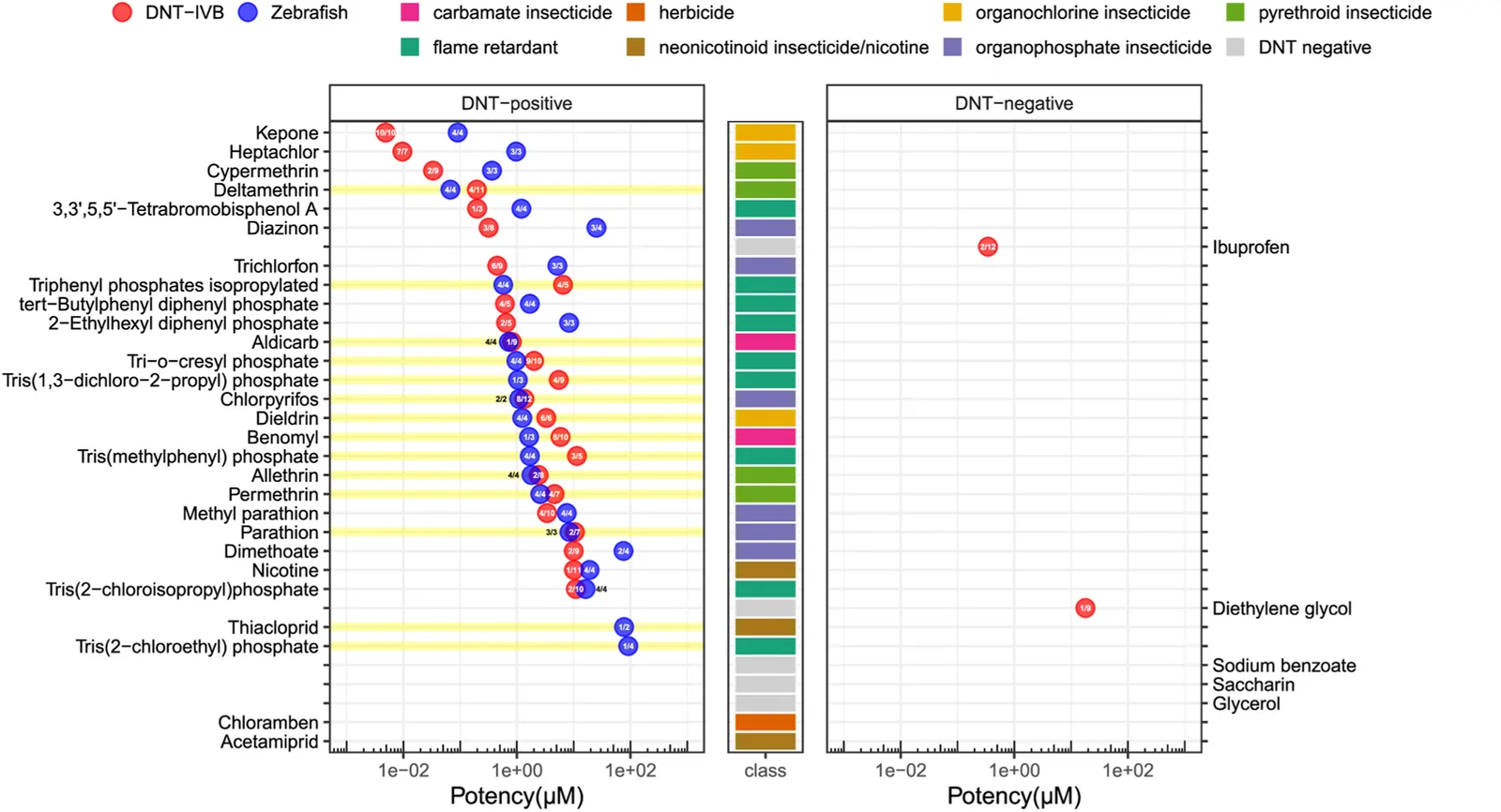
Comparative Potency Analysis of 33 Compounds in Zebrafish LDTT Assay and DNT-IVB. This plot illustrates the benchmark concentration (BMC) for zebrafish or activity at concentration cutoff (ACC) (μM) for compounds categorized as DNT-positive and DNT-negative. Each compound can have up to two dots representing its potency in each assay, with red dots for DNT-IVB and blue dots for zebrafish LDTT results. Compounds lacking dots in a given category are considered inactive. Yellow bands indicate cases where the zebrafish assay shows greater potency (i.e., a lower concentration) than the DNT-IVB assay. Compound class is also shown in the middle of the plot, with color codes representing categories. The number displayed on each dot for zebrafish represents “# of labs identified the compound as active / # of labs tested”; for DNT-IVB, it represents “# of active assays / # of assay tested”.

A total of 163 zebrafish plates passed quality control, representing all 33 compounds (28 DNT-positive and 5 DNT-negative). Due to QC exclusions, the number of laboratories contributing data per compound ranged from 2 to 4 (mean = 3.6). Additionally, tested concentration ranges varied across laboratories for some compounds (Section 2.4). Similarly, within the DNT-IVB, none of the 33 compounds were tested across all 13 assays. The number of assays per compound ranged from 2 to 12 (mean = 8.1), and the tested concentration ranges varied across assays. Fig. 6 plots the most potent zebrafish BMC (lowest value across laboratories) against the most potent DNT-IVB ACC (lowest value across assays) for each compound. Data summarization procedures are described in Sections 2.6.4 and 2.6.5, and the background data shown in Fig. 6 are available in Supplemental Table 4.

Across the 28 DNT-positive compounds, 26 showed concordant activity between the DNT-IVB and the zebrafish, demonstrating substantial agreement between zebrafish LDTT and the *in vitro* battery. Of these 26, 24 were concordantly active in both systems, while two (chloramben and acetamiprid) were concordantly inactive in both systems. The remaining two DNT-positive compounds—thiacloprid and tris(2-chloroethyl) phosphate (TCEP)—were active in zebrafish LDTT but not in the DNT-IVB. However, these compounds showed limited evidence of activity. Thiacloprid was tested in only two laboratories and was active in just one of them (Lab-B), although both of its tested plates (n = 2) from that laboratory showed activity. In contrast, TCEP was tested in all four laboratories; it was active in only 1 of 2 plates from Lab B and inactive in the remaining three laboratories (0 of 1 plate each). Both compounds showed relatively low potency in zebrafish (thiacloprid: 77.2 μM; TCEP: 91.6 μM), consistent with borderline activity near the upper limit of tested concentrations.

All five putative DNT-negative compounds were inactive in zebrafish LDTT across all four laboratories (0 of 4 laboratories active for each compound). In contrast, two DNT-negative compounds showed activity in the DNT-IVB: ibuprofen (active in 2 of 12 assays, ACC = 0.34 μM) and diethylene glycol (active in 1 of 9 assays, ACC = 17.9 μM). For ibuprofen, the observed activity originated from the CCTE_Mundy_HCI_iCellGluta_NOG assay and the CCTE_Shafer_MEA_dev assay, with one and seven active endpoints, respectively. For diethylene glycol, activity was detected in only a single assay (CCTE_Shafer_MEA_dev) and was driven by a single active endpoint, suggesting that diethylene glycol activity might be unreliable.

With five putative DNT-negative compounds, zebrafish LDTT showed complete concordance, with none exhibiting activity across the 33 plates tested in four laboratories, corresponding to 100% specificity in this dataset. In contrast, the DNT-IVB correctly classified three of the five compounds as inactive (60% specificity). While this observation suggests that the whole-organism zebrafish assay may offer higher specificity for distinguishing non-developmental neurotoxicants, this conclusion is based on a limited dataset and should be interpreted cautiously. Additional testing with a larger set of negative controls will be required to more robustly evaluate assay specificity of the LDTT.

Among the 24 compounds concordantly active in both systems, potency varied by compound. Twelve compounds were more potent in zebrafish (mean log_10_ difference = 0.42; ~2.6-fold), while 12 were more potent in the DNT-IVB (mean log_10_ difference = 0.94; ~8.7-fold). Seven compounds showed *>* 10-fold potency differences: heptachlor, diazinon, kepone, 2-ethylhexyl diphenyl phosphate (EHDP), trichlorfon, triphenyl phosphates isopropylated (IPP), and cypermethrin. Of these, six were more potent in the DNT-IVB, with only IPP showing greater potency in zebrafish. Notably, 2 of the 3 organochlorine insecticides showed some of the largest potency differences between the two systems.

Overall, the results for DNT-IVB appeared more potent, on average, across the available assays for each compound. However, when considering the combined multi-laboratory and multi-plate evidence, zebrafish LDTT, as a single whole-organism assay, identified 26 of 28 DNT-positive compounds (93% sensitivity; 24 concordantly active + 2 zebrafish-only actives), comparable to the 24 detected amongst the multi-assay DNT-IVB (86% sensitivity), while correctly identifying all five DNT-negative compounds (100% specificity vs 60% for DNT-IVB).

### 3.5. Comparison with data in OECD case studies

Ten of the 33 compounds evaluated in this study have been included in OECD Integrated Approaches to Testing and Assessment (IATA) case studies for DNT hazard characterization, spanning three chemical groups. These include one pyrethroid insecticide, deltamethrin (ENV/CBC/MONO(2022)24); two neonicotinoid insecticides, nicotine and acetamiprid (ENV/CBC/MONO(2022)27); and seven organophosphate flame retardants: tris(2-chloroethyl) phosphate (TCEP), tris(1,3-dichloro−2-propyl) phosphate (TDCIPP), tris(methylphenyl) phosphate (TMPP), tert-butylphenyl diphenyl phosphate (BPDP), 2-ethylhexyl diphenyl phosphate (EHDP), triphenyl phosphates isopropylated (IPP), and 3,3′,5,5′-tetrabromobisphenol A (TBBPA) (ENV/CBC/MONO(2022) 26). The availability of OECD case study evaluations for these compounds enables direct comparison of our multi-laboratory zebrafish results with existing weight-of-evidence assessments. It provides context for how zebrafish data can contribute to regulatory IATA frameworks as an additional data stream.

#### 3.5.1. Deltamethrin

In the OECD case study for deltamethrin, results from the DNT-IVB—particularly the MEA assay (neuronal network formation) and the NPC5 assay (oligodendrocyte differentiation) contributed to two key events (KE4: Altered Neuronal Network Function and KE5: Decreased Oligodendrocyte Differentiation) in the proposed adverse outcome pathway (AOP). Further studies on deltamethrin testing locomotor activity in zebrafish using differing protocols as described in (Kung, Richardson et al., 2015, Awoyemi, Kumar et al., 2019, Dach, Yaghoobi et al., 2019) were, in the first instance, considered to be at low risk of bias. However, after a detailed review, it was concluded that in these studies, the probability that locomotor activity was affected is lower than 66%. Therefore, these study results were not further considered in the subsequent ad hoc AOP generation.

In our multi-laboratory evaluation using agreed-upon standardized parameters, deltamethrin produced concordant DT activity in all four laboratories with low inter-laboratory potency variation (lowest BMC = 0.14 μM, 1.2-fold range). In contrast, DNT activity was observed in only two of four laboratories with substantial potency variation (lowest BMC = 0.07 μM, 26.9-fold range). Notably, zebrafish BMC values (0.07–0.14 μM) were 4–9-fold lower (more potent) than DNT-IVB BMCs for KE4 (BMC_50_= 0.5 μM) and KE5 (BMC_30_ = 0.6 μM).

#### 3.5.2. Nicotine and Acetamiprid

In the OECD case study for nicotine and acetamiprid, both compounds were reported to be largely inactive in the DNT-IVB. Consistent with this, our DNT-IVB data summary showed nicotine active in only 1 of 11 assays (ACC = 10.1 μM) and acetamiprid inactive across all 10 tested assays. In the OECD case study, neurobehavioral effects were examined in zebrafish using the embryonic coiling assay (24–48 hpf) and the larval basal swimming assay (83–120 hpf), both conducted by a single laboratory (University of Heidelberg). The resulting behavioral changes were mapped to KE4:Altered neurodevelopment. Although neither compound demonstrated significant effects in the basal swimming assay, nicotine produced a clear concentration-dependent response in the coiling assay, yielding a Lowest Continuous Effect Dose (LCED) of 1.25 μM. In contrast, acetamiprid produced less continuous and weakly dose-dependent coiling responses (LECD: 25 μM), raising considerable uncertainty regarding its neurodevelopmental activity.

In our multi-laboratory LDTT evaluation, acetamiprid was inactive for both DNT and DT endpoints in all three tested laboratories, strengthening the weight of evidence for its inactivity in zebrafish neurobehavioral assays. Nicotine showed concordant DT activity across all four laboratories (lowest BMC = 19.0 μM) but inconsistent DNT activity (active in 2 of 4 laboratories; lowest BMC = 37.1 μM).

#### 3.5.3. Flame retardants

The OECD flame retardant (FR) case study included zebrafish neurobehavioral data from three laboratories. Despite protocol diversity across laboratories, the datasets were harmonized and analyzed using a common analytical pipeline (Hsieh et al., 2018). Among the seven FR compounds overlapping with the 33 compounds tested in our study, TDCIPP had not been previously evaluated in zebrafish neurobehavior assays. In the previous OECD/DNT-DIVER dataset, tert-butylphenyl diphenyl phosphate (BPDP) and tris(methylphenyl) phosphate (TMPP) were concordantly active across all three laboratories, with the lowest reported BMC values of 4.4 μM and 4.2 μM, respectively. EHDP, IPP, and TBBPA were concordantly active in two of three laboratories, with the lowest BMCs between 2.7 and 3.8 μM. TCEP was active in only one of the three laboratories, with a lowest BMC of 17 μM.

In the current multi-laboratory study using agreed-upon standardized parameters and protocols, tris(1,3-dichloro− 2-propyl) phosphate (TDCIPP) showed activity in both DNT and DT endpoints (lowest BMCs: 1.7 μM and 1.0 μM, respectively). However, DNT activity was observed in only one of three laboratories. Among the remaining six FRs, tris(2-chloroethyl) phosphate (TCEP) again showed weak and inconsistent DNT activity (BMC = 91.6 μM in 1 of 4 laboratories). TMPP and BPDP exhibited the highest confidence: both were concordantly active for DNT and DT across all four laboratories (BMC range: 1–3 μM), consistent with the previous OECD dataset. For triphenyl phosphates isopropylated (IPP) and 3,3′,5,5′-tetrabromobisphenol A (TBBPA), the two most lipophilic compounds in the FR group (logKow*>*7), DNT activity was less consistent (active in 2 of 4 and 1 of 4 laboratories, respectively). However, DT activity was concordant for both compounds (BMC range: 0.57–2.67 μM). 2-Ethylhexyl diphenyl phosphate (EHDP), tested in three laboratories, was concordantly active in DNT (2 of 3 laboratories) and DT (3 of 3 laboratories), with BMCs ranging from 8.3 to 21.7 μM.

Across both multi-laboratory datasets (the previous OECD/DNT-DIVER dataset and the current study), TMPP and BPDP consistently emerged as the most concordantly active flame retardants, with comparable BMC ranges. This convergence substantially strengthens confidence in their DNT effects. A second tier of confidence includes EHDP, IPP, and TBBPA, all of which showed generally concordant responses with similar potency ranges across studies. TCEP demonstrated the weakest and least consistent DNT activity across both datasets, while TDCIPP, which lacked prior zebrafish behavioral data, shows potential DNT activity but requires further validation given limited inter-laboratory concordance (1 of 3 laboratories).

## 4. Discussion

The transition from traditional animal testing to New Approach Methodologies (NAMs) for developmental neurotoxicity assessment requires a robust demonstration of reproducibility across testing facilities. While *in vitro* assays have dominated recent NAM development, whole-organism models remain critical for bridging mechanistic cellular endpoints and mammalian toxicity. This study, building on the DNT-DIVER harmonization effort (Behl et al., 2019; Maass et al., 2023), demonstrates that zebrafish behavioral assessment can achieve reproducible results across laboratories when implemented with systematic quality control and coordinated protocol elements. In addition, the relatively low cost, efficiency, and rapid turnaround of a single zebrafish whole-organism assay argue for its use as an early-tier NAM—applied before the full DNT-IVB battery and mammalian guideline studies—to prioritize compounds for further testing while maintaining relevant organism-level context. By including compounds from OECD DNT IATA case studies, we demonstrate how multi-laboratory zebrafish data can strengthen weight-of-evidence evaluations and highlight where zebrafish fills gaps in existing approaches. Notably, our multi-laboratory zebrafish dataset provides a valuable counterpoint to *in vitro* battery data typically generated in single facilities, as the cross-laboratory validation inherently captures a broader range of technical variability and environmental conditions. While this multi-laboratory approach revealed reproducibility challenges that systematic QC successfully addressed, it remains an open question whether regulatory confidence in zebrafish LDTT data requires multi-laboratory validation or whether rigorously qualified single-laboratory data that meet established QC thresholds can achieve sufficient reliability. This consideration is particularly relevant given that most *in vitro* DNT battery data, despite being generated at a single facility, have gained regulatory acceptance—suggesting that with appropriate quality standards, single-laboratory zebrafish data may similarly prove adequate for certain regulatory applications.

### 4.1. Quality control framework

The systematic QC framework implemented in this study—removing 10.9% of plates that passed basic viability criteria—proved essential for managing data quality across laboratories with different facility-specific practices. Importantly, the QC approach developed here is broadly applicable beyond zebrafish: the intra-plate and inter-plate consistency metrics can be applied to any multi-well plate-based system where movement or activity data are collected, including other aquatic models (e.g., *Daphnia* (Yuan et al., 2021), fish embryos of other species (Takai et al., 2022; Albers et al., 2024)), cell-based migration assays, or even mammalian behavioral studies using multi-chamber systems.

The laboratory-specific patterns in baseline movement (tight clustering for Lab-A and Lab-D versus broader dispersion for Lab-B and Lab-C) revealed that coordinating certain protocol elements is highly valuable but cannot eliminate all sources of variability. Laboratories retained facility-specific practices in husbandry, handling techniques, and environmental conditions that influenced baseline behavioral responses. This observation has important implications for the broader adoption of behavioral assays across diverse model systems. Apart from requiring the standardization of the technically relevant procedures —which may be difficult across diverse testing facilities—our results demonstrate that data-driven QC approaches can effectively account for laboratory-specific characteristics while maintaining consistent quality thresholds.

The novel intra-plate QC metric uses movement pattern similarity among control wells to identify plates with irregular baseline behavior, while the inter-plate QC uses principal component analysis to flag statistical outliers relative to laboratory-specific distributions. Both approaches establish quality benchmarks based on empirical data distributions, making them adaptable to different assay systems with varying baseline characteristics.

For regulatory acceptance of NAMs, including behavioral endpoints, such QC frameworks will be critical (ICCVAM, 2024). They provide a mechanism for laboratories to demonstrate data quality and comparability even when facility-specific differences exist. The QC approach could be readily implemented in any laboratory with automated tracking systems, requiring only the calculation of movement similarity metrics and basic statistical analyses (PCA), which are available in standard software packages. As the field moves toward regulatory guidelines for diverse NAM platforms, incorporating robust, transferable QC procedures as integral components—rather than optional add-ons—will strengthen confidence in cross-laboratory comparability across multiple testing systems.

### 4.2. Reproducibility: Progress and remaining challenges

DT endpoints showed high reproducibility across laboratories and concordance (84–100% pairwise agreement) while several hydrophilic compounds also demonstrated discordant DT activity, suggesting that not all variability can be explained by the chemical characteristics assessed here. DNT calls were more variable (68–84%), though potency variation for active compounds was similar (mean 3.2-fold for DNT vs 3.5-fold for DT). This indicates variability manifests primarily in binary active/inactive classification rather than potency estimates. Importantly, inter‑study differences in point‑of‑departure metrics of up to one order of magnitude are commonly observed and considered acceptable in developmental toxicity testing, suggesting that the 3–4‑fold BMC differences seen here fall well within expected experimental variability (Braakhuis et al., 2019; Paul Friedman et al., 2023). Our concordance rates align with DNT-DIVER (NTP, 2018; Hsieh et al., 2022), in which DT concordance ranged from 83 to 94% and NT/DNT concordance ranged from 77 to 85%. Despite different approaches, the consistency suggests inherent assay robustness that supports regulatory adoption through multiple pathways.

An important caveat is that laboratories tested different numbers of plates per compound, and our comparison selected the most active plate from each laboratory (Section 2.6.4). This approach may inflate apparent concordance because our dataset is enriched for putative DNT-positive compounds; therefore, our findings should be interpreted as demonstrating feasibility and promise rather than definitive validation. Formal validation would require all laboratories to test identical replicates and account for within-laboratory variability in statistical analyses. Nevertheless, it is noteworthy that the same data summary approach was applied to all plates from the DNT-negative compounds, and none were classified as active, providing additional confidence in the assay’s specificity.

The lower DNT concordance indicates sensitivity to uncontrolled variation. Our analysis found no single factor (lipophilicity, exposure amount, active endpoint number) that fully explained variability. Several factors that differed across laboratories including instrumentation differences, including tracking detection algorithms, camera sensitivity, and lighting conditions, may contribute to variability in the detection of subtle behavioral changes and are documented in Supplemental Table 1 Additionally, unstandardized factors (husbandry, handling, environmental conditions) may have influenced baseline behavioral sensitivity. Statistical power may be insufficient for compounds with modest effects, based on our companion power analysis (Konrad et al., 2026); 8–32 embryos per concentration is needed to achieve 80% power for a 20% effect size, and variability in the number of QC-passing larvae per plate may have contributed to inconsistent activity calls for borderline compounds.

Importantly, biological variability among individual larvae may represent a meaningful signal rather than merely technical noise (Jacobs and Ryu, 2023). Zebrafish lines are typically not isogenic, introducing genetic diversity that more closely resembles human populations than inbred rodent strains (Adams et al., 2020; Franěk et al., 2020). For regulatory toxicology aimed at protecting genetically diverse populations, this variability may reflect biologically relevant differences in chemical susceptibility. The challenge lies in distinguishing technical variability (to be minimized through standardization and QC) from biological variability (to be measured and characterized as informative). This is complicated by laboratories employing different strategies to manage variability. Variance component studies can help identify these sources and determine whether variability primarily reflects control-lable technical factors or biological individuality, which requires appropriate statistical approaches and sample sizes. Addressing these questions is essential for advancing toward guideline status, though current reproducibility supports weight-of-evidence applications and tiered screening.

### 4.3. Biological integration: DNT-DT overlap in whole organisms

The substantial DNT-DT potency overlap (median log_10_ difference near zero) may reflect biological integration rather than assay limitation, but warrants careful interpretation in the context of this study design. Unlike *in vitro* systems, where functional endpoints are tested at sub-cytotoxic concentrations to isolate mechanisms (Escher, Henneberger et al., 2020; Blum et al., 2023), separating “primary” neurotoxicity from “secondary” developmental effects in intact organisms may not be biologically meaningful. From a regulatory perspective, compounds causing behavioral alterations at concentrations that also produce morphological effects remain of concern, as both would manifest *in vivo* (OECD, 2007).

The near-zero BMC overlap also warrants mechanistic consideration. While malformed larvae were excluded from behavioral analysis prior to BMC modeling, the 96-well square well plate format used across all laboratories provides a larger water volume per well than round wells used in prior work, which may affect chemical bioavailability and introduce variability in effective internal concentrations at the organism level and adsorption to well walls. Combined with the modest per-chemical replication available after QC filtering in a multi-laboratory setting, this likely reduced the precision of behavioral BMC estimates — particularly for chemicals where the dynamic range of vehicle control responses was variable across plates. Together, these factors can compress the apparent separation between DNT and DT potency estimates even when true biological selectivity exists. Notably, single-laboratory zebrafish LDTT studies with sufficient replication have demonstrated clear selectivity for DNT endpoints relative to DT, supporting the assay’s specificity for neurobehavioral disruption independent of gross morphological effects, and suggesting that the near-zero overlap observed here reflects the practical constraints of this multi-laboratory design rather than a fundamental property of the assay (Hagstrom et al., 2019; Gutsfeld et al., 2024).

A related interpretive consideration in continuous-exposure paradigms such as ours is the potential contribution of acute pharmacological effects to observed behavioral changes. Because larvae remain in chemical-containing medium throughout the 6–120 hpf exposure period and are assessed behaviorally at 120 hpf without an intervening washout step, it is not possible to fully distinguish developmental programming effects, changes arising from chemical perturbation of neural circuit formation during sensitive developmental windows, from acute effects of the chemical acting directly on the nervous system at the time of behavioral testing (Gutsfeld et al., 2024). A washout design, in which larvae are transferred to clean medium prior to assessment, would be required to formally separate these contributions. We note, however, that this interpretive challenge is not unique to zebrafish as functional endpoints in the DNT-IVB battery are similarly assessed in the continued presence of the test chemical, without a washout step, and are broadly accepted as reflecting the net biological consequence of exposure during critical developmental windows. Systematic washout studies comparing behavioral outcomes before and after chemical clearance in addition to tissue concentration, would nonetheless be a valuable direction for future work to clarify the relative contribution of acute versus developmental effects across chemical classes and to further characterize the specificity of the zebrafish LDTT as a DNT tool.

Taken together, these considerations position the zebrafish LDTT as an integrative screen capturing functional consequences of developmental perturbations rather than a mechanism-specific assay (Ton et al., 2006). It bridges mechanistic *in vitro* screening (isolating pathways) and mammalian studies (organism-level outcomes) and is therefore well-suited as a first-tier whole-organism NAM, in which combined DNT/DT responses provide an initial point of departure for regulatory use, with the DNT-IVB applied subsequently to active compounds to elucidate underlying mechanisms. Compounds showing DNT without DT represent the highest follow-up priority (specific neurotoxic mechanisms), those showing both indicate general developmental disruption with behavioral consequences, and those showing only DT suggest morphological changes without detectable functional deficits at this life stage. Future refinements such as testing earlier developmental windows (e.g., 6–24 vs 6–120 hpf), incorporating additional behavioral paradigms, and integrating mechanistic endpoints, could enhance neurotoxicity specificity, though the current assay already provides valuable organism-level integration that complements mechanism-specific approaches.

### 4.4. Efficiency and Complementarity in Tiered Testing

Zebrafish LDTT achieved comparable sensitivity (detecting 93% of DNT-positive compounds) and superior specificity (100% vs 60% correct identification of negatives) relative to the DNT-IVB – the battery comprising 13 assays and 64 endpoints (Carstens et al., 2025). This efficiency enables laboratories to screen substantially more compounds with zebrafish than the full *in vitro* battery, and additional plates per compound could be tested to improve statistical power for borderline effects. The higher specificity reduces the false positive burden, allowing resources to focus on compounds with genuine DNT concern.

This represents the first multi-laboratory evaluation of any DNT testing approach beyond individual *in vitro* assays, with zebrafish now validated across two independent efforts (NTP, 2018). The demonstrated reproducibility, combined with moderate cost and throughput capacity, supports multiple strategic uses of zebrafish within tiered frameworks rather than a single fixed position.

As understanding of the zebrafish model advances, several complementary applications emerge. Zebrafish can serve as an initial whole-organism screen prior to *in vitro* testing (tier 1), providing early organism-level context that helps prioritize compounds and interpret subsequent mechanistic findings. This approach leverages zebrafish’s ability to integrate metabolism, distribution, barriers, and compensatory mechanisms to identify compounds that produce functional consequences in an intact system. Alternatively, zebrafish can be used to validate mechanistic predictions, confirm that cellular-level perturbations translate into organism-level effects, and eliminate assay-specific artifacts as false positives. The two compounds active only in DNT-IVB (ibuprofen, diethylene glycol—both putative DNT-negatives showing isolated *in vitro* activity) underscore the value of whole-organism validation (Jones et al., 2012), but further testing of additional compounds is needed to distinguish true positives from assay-specific artifacts. A third application positions zebrafish alongside *in vitro* approaches, providing parallel organism-level evidence that complements mechanistic data for weight-of-evidence evaluation.

The optimal positioning depends on specific regulatory or research contexts. For large-scale screening programs prioritizing throughput, zebrafish could serve as a first-pass filter before committing resources to multi-assay *in vitro* batteries. For compounds with existing *in vitro* data, zebrafish provides organism-level validation. For IATA evaluations that require multiple evidence streams, zebrafish provides complementary whole-organism data. The two compounds active only in zebrafish (thiacloprid (Xie et al., 2022a, 2022b), TCEP (Sutha et al., 2024), though with limited inter-laboratory reproducibility, suggest potential sensitivity to metabolic activation or intact organismal context (Xie et al., 2022a, 2022b; Hu et al., 2023), illustrating unique information that whole-organism assessment can provide.

Rather than prescribing a single-tier placement, these findings support flexible integration of zebrafish based on testing objectives, available resources, and the need to balance mechanistic understanding with organism-level functional outcomes. As the model continues to be refined and validated, this flexibility will enable optimization of tiered strategies for diverse regulatory and research applications.

### 4.5. Strengthening OECD IATA evaluations

The three OECD case studies ((ENV/CBC/MONO(2022)24), (ENV/CBC/MONO(2022)27), (ENV/CBC/MONO(2022)26)) (OECD, 2022a, 2022b, 2022c) illustrate how zebrafish data contribute to regulatory evaluations under different evidentiary contexts. For flame retardants, the OECD case study successfully incorporated DNT-DIVER multi--laboratory zebrafish data, demonstrating that when reproducibility is established, behavioral data can serve as a credible line of evidence in IATA frameworks (Kreutz et al., 2024). Our independent validation of these findings—showing concordance for the same compounds across two separate multi-laboratory efforts—establishes a confidence tier that regulatory decision-makers can use to weigh the strength of the evidence. The convergence of findings from two independent multi-laboratory efforts strengthens the evidence base for the DNT potential of several flame retardants, particularly TMPP and BPDP. It supports the utility of zebrafish LDTT for regulatory DNT assessment (Maass et al., 2023; Hernandez-Jerez et al., 2024).

The deltamethrin and nicotine cases highlight the complexity of multi-laboratory testing with chemically challenging compounds. Both showed concordant DT but variable DNT activity in our study, noted in the published zebrafish literature (Borrego-Soto and Eberhart, 2022), as in the OECD deltamethrin case study reviewed. Several factors beyond assay performance may contribute to this variability. For both compounds, the exposures in the literature were conducted differently, which could contribute to the variable DNT calls (Borrego-Soto and Eberhart, 2022; Lei et al., 2022). Deltamethrin’s high lipophilicity (logKow 6.2) and potential chemical instability during aqueous exposure could result in variable internal concentrations across laboratories despite identical nominal concentrations, thereby affecting detection sensitivity. Similarly, nicotine’s discordant DNT activity (concordant across laboratories in the earlier embryonic coiling assay but variable in the 120 hpf LDTT) may reflect developmental-stage-specific sensitivity windows rather than assay limitations. Nicotine has a short half-life and is cleared rapidly by liver metabolism (Benowitz, 2009). For these reasons, the presence of an effect in zebrafish when exposure occurred 5 days prior can contribute to variability, whereas the embryonic coiling assay is conducted within 1 day post-exposure (von Hellfeld et al., 2023). These patterns underscore the importance of measuring actual internal tissue concentrations and testing across multiple developmental stages to fully characterize compound effects.

For nicotine and acetamiprid, where DNT-IVB showed limited activity, zebrafish provides a valuable organism-level assessment. Acetamiprid’s consistent inactivity across all laboratories strengthens confidence in this negative call. At the same time, nicotine’s developmental-stage-dependent responses indicate that a comprehensive DNT assessment may require behavioral batteries spanning multiple time points, analogous to how DNT-IVB uses multiple assays to capture different biological processes.

These comparisons demonstrate that zebrafish’s regulatory value lies in providing complementary organism-level validation, particularly for certain compound classes (flame retardants), and in confirming negative calls (acetamiprid). The key advance is establishing transparent confidence frameworks based on multi-laboratory reproducibility—enabling evidence integration in IATA evaluations that appropriately weights data quality. Where variability occurs, it identifies research needs (internal concentration measurement, developmental stage optimization, chemical stability assessment) that can guide future method refinement.

### 4.6. Path forward: From screening tool to guideline method

Current reproducibility and specificity support the use of zebrafish LDTT for immediate regulatory applications in weight-of-evidence approaches and tiered screening. Advancing toward guideline status requires strategic validation efforts that build on the foundation established by this study and DNT-DIVER. A formal ring trial represents the critical next step for regulatory acceptance. Such a validation study, with all laboratories testing identical compounds at predetermined replicate numbers under fully standardized conditions, would establish definitive performance benchmarks, acceptance criteria, and proficiency standards required for guideline methods. Reference compounds spanning a range of potencies would enable laboratories to demonstrate competency and comparability, providing the rigorous validation framework regulatory agencies require.

Our recent publication on power calculation (Konrad et al., 2026) can assist on optimizing experimental designs, though an important consideration for zebrafish is that they are living organisms in dynamic developmental states. Unlike chemical or cell-based assays, experimental duration and chamber conditions (plate format, volume, density) directly impact organism physiology and behavior. Understanding how these biological factors interact with statistical power requirements is essential for designing efficient yet biologically appropriate protocols.

Exposure characterization remains a priority for interpreting variability, particularly measuring internal tissue concentrations to clarify whether inter-laboratory differences reflect biological variation or differential chemical uptake and stability. Systematic investigation of unstandardized factors (husbandry practices, handling techniques, environmental conditions) would identify critical control points for future harmonization. Expanded chemical coverage across diverse modes of action and the evaluation of multiple developmental-stage windows would further validate the assay’s scope and sensitivity.

Together, these priorities provide a roadmap for advancing zebrafish behavioral assessment from its demonstrated utility as a screening tool toward broader regulatory guideline acceptance. Within such tiered strategies, zebrafish-based endpoints—including the LDTT described here as well as additional current and future behavioral or morphological assays—would function as primary whole-organism screens, providing organism-level points of departure and helping to target downstream mechanistic DNT-IVB testing and mammalian studies to those compounds where additional refinement is most needed. The convergence of findings across independent multi-laboratory efforts establishes a strong foundation for this transition and supports the progressive integration of zebrafish endpoints into formal DNT testing frameworks.

## 5. Conclusions

This multi-laboratory evaluation demonstrates that zebrafish LDTT with systematic QC and coordinated protocols achieves reproducible DNT assessment suitable for regulatory weight-of-evidence approaches, with comparable sensitivity and increased specificity to multi-assay *in vitro* batteries. Important challenges remain before guideline standardization, particularly for compounds with modest effects or challenging chemical properties. However, convergence across two independent efforts establishes zebrafish as a reproducible, practical tool that bridges high-throughput mechanistic screening and mammalian studies while capturing the biological integration essential for predicting *in vivo* effects in rodents and humans, and central nervous system effects. Integration with IATA case studies demonstrates how zebrafish strengthens regulatory evaluations by validating *in vitro* predictions, filling gaps, and enabling tiered confidence frameworks. With systematic investigation of remaining variability sources and continued testing of a broader set of well-characterized DNT-positive and DNT-negative compounds, zebrafish behavioral assessment has strong potential to advance from a screening tool toward broader regulatory acceptance as a component of DNT testing batteries.

## Supplementary Material

MMC7

MMC2

MMC6

MMC5

MMC3

MMC4

MMC1

## Figures and Tables

**Table 1:** Chemical Information for the 33 Compounds Evaluated in the Zebrafish LDTT Study. The compounds are sorted based on LogKow.

DTXSID	CASRN	Chemical Name	Molecular Weight	Supplier	Lot Number	CoA Purity (%)	LogKoW	Use Class	Potential MOA for DNT	OECD publication

DTXSID8020462	111–46–6	Diethylene glycol	106.12	Sigma-Aldrich	01824CJ	99.4	−1.22			
DTXSID7034961	111988–49–9	Thiacloprid	252.72	Toronto Research Chemicals	23-ABY-4–1	98	1.26	neonicotinoid insecticide/nicotine	nicotinic acetylcholine receptor agonist	
DTXSID5021411	115–96–8	Tris(2-chloroethyl) phosphate (TCEP)	285.49	Sigma-Aldrich	07430JR	98.8	1.44	flame retardant		ENV/CBC/MONO(2022) 26
DTXSID0039223	116–06–3	Aldicarb	190.3	Chem Service Inc.	7110400	99.5	1.13	carbamate insecticide	acetylcholinesterase inhibitor	
DTXSID1025300	1241 –94–7	2-Ethylhexyl diphenyl phosphate	362.41	TCI America	BKUNE	92.8	5.73	flame retardant		ENV/CBC/MONO(2022) 26
DTXSID4021391	1330–78–5	Tris(methylphenyl) phosphate (TMPP)	368.36	Acros Organics via Fisher Scientific	A0271226	99 Sum of Isomers	5.11	flame retardant		ENV/CBC/MONO(2022) 26
DTXSID2020262	133–90–4	Chloramben	206.02	TCI America	PSJUA	98	1.9	herbicide	auxin-type growth regulator	
DTXSID0034300	135410–20–7	Acetamiprid	222.67	Toronto Research Chemicals	10-MIC-74–1	98	0.8	neonicotinoid insecticide/nicotine	nicotinic acetylcholine receptor agonist	ENV/CBC/MONO(2022) 27
DTXSID5026259	13674–84–5	Tris(2-chloroisopropyl) phosphate (TCPP)	327.57	Albemarle Corporation via MRIGlobal	M072911NP	Unknown	2.59	flame retardant		
DTXSID9026261	13674–87–8	Tris(1,3-dichloro–2-propyl) phosphate (TDCIPP)	430.89	TCI America	EFGBE	95.4	3.65	flame retardant		ENV/CBC/MONO(2022) 26
DTXSID1020770	143–50–0	Kepone	490.6	Chem Service Inc.	9035500	99.4	5.41	organochlorine insecticide	GABAergic and dopaminergic modulator + oxidative stress	
DTXSID5020732	15687–27–1	Ibuprofen	206.28	Sigma-Aldrich	026H1368	99.8	3.74			
DTXSID5023900	17804–35–2	Benomyl	290.32	Sigma-Aldrich	MKCC3036	~ 99.5†	2.12	carbamate insecticide	acetylcholinesterase inhibitor	
DTXSID4020458	2921–88–2	Chlorpyrifos	350.59	Chem Service Inc.	7230400	99.5	4.98	organophosphate insecticide	acetylcholinesterase inhibitor	
DTXSID1020855	298–00–0	Methyl parathion	263.21	Supelco via Sigma Aldrich	LC22545V	99.9	2.86	organophosphate insecticide	acetylcholinesterase inhibitor	
DTXSID9020407	333–41–5	Diazinon	304.35	Toronto Research Chemicals	22-GHZ-116–1	98	3.81	organophosphate insecticide	acetylcholinesterase inhibitor	
DTXSID1023998	52315–07–8	Cypermethrin	416.3	Alfa Aesar via Fisher Scientific	W30A048	95.4	6.6	pyrethroid insecticide	voltage-gated sodium channel blocker	
DTXSID8022292	52645–53–1	Permethrin	391.31	Chem Service Inc.	9035600	77.3 trans; 22.5 cis	6.5	pyrethroid insecticide	voltage-gated sodium channel blocker	
DTXSID0021389	52–68–6	Trichlorfon	257.44	Chem Service Inc.	9035700	99.5	0.51	organophosphate insecticide	acetylcholinesterase inhibitor	
DTXSID8020381	52918–63–5	Deltamethrin	505.2	Toronto Research Chemicals	17-ANR-63–1	98	6.2	pyrethroid insecticide	voltage-gated sodium channel blocker	ENV/CBC/MONO(2022) 24
DTXSID1020930	54–11–5	Nicotine	162.23	Sigma-Aldrich	068K1252	98.9	1.17	neonicotinoid insecticide/nicotine	nicotinic acetylcholine receptor agonist	ENV/CBC/MONO(2022) 27
DTXSID7021100	56–38–2	Parathion	291.26	Supelco via Sigma Aldrich	LC12215V	96	3.83	organophosphate insecticide	acetylcholinesterase inhibitor	
DTXSID6024701	56803–37–3	tert-Butylphenyl diphenyl phosphate (BPDP)	382.39	Ubichem PLC via MRIGlobal	M062011NS	Unknown	5.12	flame retardant		ENV/CBC/MONO(2022) 26
DTXSID8035180	584–79–2	Allethrin	302.41	Toronto Research Chemicals	3-BLH-130–1	96	4.78	pyrethroid insecticide	voltage-gated sodium channel blocker	
DTXSID7020479	60–51–5	Dimethoate	229.26	Toronto Research Chemicals	1-CGS–8–1	98	0.78	organophosphate insecticide	acetylcholinesterase inhibitor	
DTXSID9020453	60–57–1	Dieldrin	380.91	Sigma-Aldrich	01720DT	92.9	5.4	organochlorine insecticide	GABAergic and dopaminergic modulator + oxidative stress	
DTXSID4028880	68937–41–7	Triphenyl phosphates isopropylated (IPP)	452.52	ICL-IP America Inc.	10335H0302	Not Listed	9.07	flame retardant		ENV/CBC/MONO(2022) 26
DTXSID3020679	76–44–8	Heptachlor	373.32	Radian International	32455–05	99	6.1	organochlorine insecticide	GABAergic and dopaminergic modulator + oxidative stress	
DTXSID6032192	78–30–8	Tri-o-cresyl phosphate	368.36	Acros Organics via Fisher Scientific	A016705201	96.8	6.34	flame retardant		
DTXSID1026081	79–94–7	3,3′,5,5′-Tetrabromobisphenol A (TBBPA)	543.87	Sigma-Aldrich	03816DJ	98.8	7.2	flame retardant		ENV/CBC/MONO(2022) 26
DTXSID5021251	81–07–2	Saccharin	183.18	Sigma-Aldrich	STBK6758	> 99.9	0.91			

* as DNT-negative (n = 5). The remainder (n = 28) is considered DNT-positive.

**Table 2:** Analytical endpoints and their corresponding phases and definitions.

Endpoint Type	Phase	Definition	# of Endpoints

Movement Similarity	Across all phases	The Spearman correlation between each larva and the VC median. Median calculated per plate.	1
Distance Change	Light 1 (L1) Dark 1 (D1) Light 2 (L2) Dark 2 (D2)	Measure of the magnitude of difference between each larva and the median of its plate’s VCs. The area between the larva and VC median curve. Median calculated per plate.	4
Distance Shift (hyperactivity)	Light 1 (L1) Dark 1 (D1) Light 2 (L2) Dark 2 (D2)	Measure of the magnitude and increased direction of difference between each larva and the VC median. Median calculated per plate.	4
Distance Shift (hyporactivity)	Dark 1 (D1) Dark 2 (D2)	Measure of the magnitude and decreased direction of difference between each larva and the VC median. Median calculated per plate.	2

## Data Availability

Data will be made available on request.

## Appendix A. Supporting information

Supplementary data associated with this article can be found in the online version at doi:10.1016/j.neuro.2026.103504.

## References

[R1] Adams LG, Gordon MS, Buth DG, Hutchings EM, 2020. A comparison of isogenic homozygous clone and Wildtype Zebrafish (Danio rerio): survival and developmental responses to low pH conditions. Zebrafish. 10.1089/zeb.2019.1780 32315581

[R2] Albers JL, Ivan LN, Clark BW, Nacci DE, Klingler RH, Thrash A, Steibel JP, Vinas NG, Carvan MJ, Murphy CA, 2024. Impacts on Atlantic killifish from neurotoxicants: genes, behavior, and population-relevant outcomes. Environ. Sci. Technol. 58 (39), 17235–17246. 39287556 10.1021/acs.est.4c04207 PMC11447911

[R3] Alzualde A, Behl M, Sipes NS, Hsieh JH, Alday A, Tice RR, Paules RS, Muriana A, Quevedo C, 2018. Toxicity profiling of flame retardants in zebrafish embryos using a battery of assays for developmental toxicity, neurotoxicity, cardiotoxicity and hepatotoxicity toward human relevance. Neurotoxicol. Teratol. 70, 40–50. 30312655 10.1016/j.ntt.2018.10.002

[R4] Awoyemi OM, Kumar N, Schmitt C, Subbiah S, Crago J, 2019. Behavioral, molecular and physiological responses of embryo-larval zebrafish exposed to types I and II pyrethroids. Chemosphere 219, 526–537. 30553213 10.1016/j.chemosphere.2018.12.026

[R5] Bal-Price AK, Coecke S, Costa L, Crofton KM, Fritsche E, Goldberg A, Grandjean P, Lein PJ, Li A, Lucchini R, Mundy WR, Padilla S, Persico AM, Seiler AE, Kreysa J, 2012. Advancing the science of developmental neurotoxicity (DNT): testing for better safety evaluation. Altex 29 (2), 202–215. 22892558 10.14573/altex.2012.2.202

[R6] Behl M, Ryan K, Hsieh J-H, Parham F, Shapiro AJ, Collins BJ, Sipes NS, Birnbaum LS, Bucher JR, Foster PMD, Walker NJ, Paules RS, Tice RR, 2019. Screening for Developmental Neurotoxicity at the National Toxicology Program: The Future Is Here. Toxicol. Sci. 167 (1), 6–14. 30496580 10.1093/toxsci/kfy278 PMC6657567

[R7] Benowitz NL, 2009. Pharmacology of nicotine: addiction, smoking-induced disease, and therapeutics. Annu. Rev. Pharmacol. Toxicol. 49, 57–71. 18834313 10.1146/annurev.pharmtox.48.113006.094742 PMC2946180

[R8] Blum J, Masjosthusmann S, Bartmann K, Bendt F, Dolde X, Dönmez A, Förster N, Holzer AK, Hübenthal U, Keßel HE, Kilic S, Klose J, Pahl M, Stürzl LC, Mangas I, Terron A, Crofton KM, Scholze M, Mosig A, Leist M, Fritsche E, 2023. Establishment of a human cell-based in vitro battery to assess developmental neurotoxicity hazard of chemicals. Chemosphere 311 (Pt2), 137035. 10.1016/j.chemosphere.2022.137035 36328314

[R9] Borrego-Soto G, Eberhart JK, 2022. Embryonic nicotine exposure disrupts adult social behavior and craniofacial development in zebrafish. Toxics 10 (10). 10.3390/toxics10100612 PMC9611253 36287892

[R10] Braakhuis HM, Theunissen PT, Slob W, Rorije E, Piersma AH, 2019. Testing developmental toxicity in a second species: are the differences due to species or replication error? Regul. Toxicol. Pharmacol. 107, 104410. 10.1016/j.yrtph.2019.104410 31226390

[R11] Carstens KE, Dönmez A, Hsieh J-H, Bartmann K, Friedman KP, Koch K, Scholze M, Fritsche E, 2025. A comparative study of biostatistical pipelines for benchmark concentration modeling of in vitro screening assays. Comput. Toxicol. 34, 100360. 10.1016/j.comtox.2025.100360 PMC12261583 40672902

[R12] Cassar S, Adatto I, Freeman JL, Gamse JT, Iturria I, Lawrence C, Muriana A, Peterson RT, Van Cruchten S, Zon LI, 2020. Use of Zebrafish in Drug Discovery Toxicology. Chem. Res. Toxicol. 33 (1), 95–118. 31625720 10.1021/acs.chemrestox.9b00335 PMC7162671

[R13] Collins ES, Hessel EVS, Hughes S, 2024. How neurobehavior and brain development in alternative whole-organism models can contribute to prediction of developmental neurotoxicity. Neurotoxicology 102, 48–57. 38552718 10.1016/j.neuro.2024.03.005 PMC11139590

[R14] Cornet C, Calzolari S, Miñana-Prieto R, Dyballa S, van Doornmalen E, Rutjes H, Savy T, D’Amico D, Terriente J, 2017. ZeGlobalTox: An Innovative Approach to Address Organ Drug Toxicity Using Zebrafish. Int. J. Mol. Sci. 18 (4). 10.3390/ijms18040864 PMC5412445 28422076

[R15] Dach K, Yaghoobi B, Schmuck MR, Carty DR, Morales KM, Lein PJ, 2019. Teratological and Behavioral Screening of the National Toxicology Program 91-Compound Library in Zebrafish (Danio rerio). Toxicol Sci 167 (1), 77–91. 30364989 10.1093/toxsci/kfy266 PMC6317431

[R16] Escher BI, Henneberger L, König M, Schlichting R, Fischer FC, 2020. Cytotoxicity Burst? Differentiating Specific from Nonspecific Effects in Tox21 in Vitro Reporter Gene Assays. Env. Health Perspect 128 (7), 77007. 32700975 10.1289/EHP6664 PMC7377237

[R17] European Parliament C o t E.U., 2010. Directive 2010/63/EU on the protection of animals used for scientific purposes, L276. Official Journal of the European Union, Brussels, Belgium, pp. 233–279. Directive 2010/63/EU.

[R18] Franěk R, Baloch AR, Kašpar V, Saito T, Fujimoto T, Arai K, Pšenička M, 2020. Isogenic lines in fish – a critical review. Rev. Aquac. 12 (3), 1412–1434.

[R19] Fritsche E, Grandjean P, Crofton KM, Aschner M, Goldberg A, Heinonen T, Hessel EVS, Hogberg HT, Bennekou SH, Lein PJ, Leist M, Mundy WR, Paparella M, Piersma AH, Sachana M, Schmuck G, Solecki R, Terron A, Monnet-Tschudi F, Wilks MF, Witters H, Zurich MG, Bal-Price A, 2018. Consensus statement on the need for innovation, transition and implementation of developmental neurotoxicity (DNT) testing for regulatory purposes. Toxicol. Appl. Pharmacol. 354, 3–6. 29447839 10.1016/j.taap.2018.02.004 PMC6097873

[R20] Gutsfeld S, Wehmas L, Omoyeni I, Schweiger N, Leuthold D, Michaelis P, Howey XM, Gaballah S, Herold N, Vogs C, Wood C, Bertotto L, Wu GM, Klüver N, Busch W, Scholz S, Schor J, Tal T, 2024. Investigation of Peroxisome Proliferator-Activated Receptor Genes as Requirements for Visual Startle Response Hyperactivity in Larval Zebrafish Exposed to Structurally Similar Per- and Polyfluoroalkyl Substances (PFAS). Environ. Health Perspect. 132 (7), 77007. 39046251 10.1289/EHP13667 PMC11268134

[R21] Hagstrom D, Truong L, Zhang S, Tanguay R, Collins ES, 2019. Comparative Analysis of Zebrafish and Planarian Model Systems for Developmental Neurotoxicity Screens Using an 87-Compound Library. Toxicol. Sci. 167 (1), 15–25. 30011007 10.1093/toxsci/kfy180 PMC6317421

[R22] Hamm JT, Hsieh JH, Roberts GK, Collins B, Gorospe J, Sparrow B, Walker NJ, Truong L, Tanguay RL, Dyballa S, Minana R, Schiavone V, Terriente J, Weiner A, Muriana A, Quevedo C, Ryan KR, 2024. Interlaboratory Study on Zebrafish in Toxicology: Systematic Evaluation of the Application of Zebrafish in Toxicology’s (SEAZIT’s) Evaluation of Developmental Toxicity. Toxics 12 (1). 10.3390/toxics12010093 PMC10820928 38276729

[R23] Hernandez-Jerez A, Coja T, Paparella M, Price A, Henri J, Focks A, Louisse J, Terron A, Binaglia M, Guajardo IM, Mangas I, Guajardo IM, Ferreira L, Kardassi D, De Lentdecker C, Molnar T, Vianello G, 2024. Statement on the toxicological properties and maximum residue levels of acetamiprid and its metabolites. Efsa J. 22 (5), e8759. 38751503 10.2903/j.efsa.2024.8759 PMC11094581

[R24] Hsieh J-H, Ryan K, Sedykh A, Lin J-A, Shapiro AJ, Parham F, Behl M, 2018. Application of Benchmark Concentration (BMC) Analysis on Zebrafish Data: A New Perspective for Quantifying Toxicity in Alternative Animal Models. Toxicol. Sci. 167 (1), 92–104. 10.1093/toxsci/kfy258 PMC6317423 30321397

[R25] Hsieh J-H, Behl M, Parham F, Ryan K, 2022. Exploring the Influence of Experimental Design on Toxicity Outcomes in Zebrafish Embryo Tests. Toxicol. Sci. 188 (2), 198–207. 35639960 10.1093/toxsci/kfac053 PMC9609873

[R26] Hu F, Li W, Wang H, Peng H, He J, Ding J, Zhang W, 2023. Environmentally relevant concentrations of tris (2-chloroethyl) phosphate (TCEP) induce hepatotoxicity in zebrafish (Danio rerio): a whole life-cycle assessment. Fish. Physiol. Biochem. 49 (6), 1421–1433. 37950834 10.1007/s10695-023-01265-7

[R27] Hughes S, Hessel EVS, 2024. Zebrafish and nematodes as whole organism models to measure developmental neurotoxicity. Crit. Rev. Toxicol. 54 (5), 330–343. 38832580 10.1080/10408444.2024.2342448

[R28] ICCVAM, 2024. Validation, Qualification, and Regulatory Acceptance of New Approach Methodologies. National Toxicology Program, Research Triangle Park, NC, USA.

[R29] Irons TD, MacPhail RC, Hunter DL, Padilla S, 2010. Acute neuroactive drug exposures alter locomotor activity in larval zebrafish. Neurotoxicol. Teratol. 32 (1), 84–90. 19465114 10.1016/j.ntt.2009.04.066

[R30] Jacobs EAK, Ryu S, 2023. Larval zebrafish as a model for studying individual variability in translational neuroscience research. Front. Behav. Neurosci. 17, 1143391. 10.3389/fnbeh.2023.1143391 PMC10328419 37424749

[R31] Jeffers A, Konrad K, Larson G, Allen-Moyer K, Cunny H, Shockley K, 2024. Simulation methodologies to determine statistical power in laboratory animal research studies. Lab. Anim. 58 (5), 486–492. 39315534 10.1177/00236772241273002 PMC11588134

[R32] Jones HS, Trollope HT, Hutchinson TH, Panter GH, Chipman JK, 2012. Metabolism of ibuprofen in zebrafish larvae. Xenobiotica 42 (11), 1069–1075. 22594345 10.3109/00498254.2012.684410

[R33] Juberg DR, Fox DA, Forcelli PA, Kacew S, Lipscomb JC, Saghir SA, Sherwin CM, Koenig CM, Hays SM, Kirman CR, 2023. A perspective on In vitro developmental neurotoxicity test assay results: An expert panel review. Regul. Toxicol. Pharmacol. 143, 105444. 10.1016/j.yrtph.2023.105444 37442267

[R34] Konrad KS, Allen-Moyer K, Ellis L, Hessel E, Jaka O, Muriana A, Martinez BM, Del Pozo A, Schiavone V, Di Donato V, Sanz CM, Truong L, Tanguay R, Shockley KR, Ryan K, Hsieh JH, 2026. Power calculations for larval zebrafish in light-dark transition test for developmental neurotoxicity. Neurotoxicology 112, 103371. 10.1016/j.neuro.2025.103371 PMC12776591 41448279

[R35] Kreutz A, Oyetade OB, Chang X, Hsieh JH, Behl M, Allen DG, Kleinstreuer NC, Hogberg HT, 2024. Integrated Approach for Testing and Assessment for Developmental Neurotoxicity (DNT) to Prioritize Aromatic Organophosphorus Flame Retardants. Toxics 12 (6). 10.3390/toxics12060437 PMC11209292 38922117

[R36] Kung TS, Richardson JR, Cooper KR, White LA, 2015. Developmental Deltamethrin Exposure Causes Persistent Changes in Dopaminergic Gene Expression, Neurochemistry, and Locomotor Activity in Zebrafish. Toxicol Sci 146 (2), 235–243. 25912032 10.1093/toxsci/kfv087 PMC4517053

[R37] Lauwereyns J, Bajramovic J, Bert B, Camenzind S, De Kock J, Elezović A, Erden S, Gonzalez-Uarquin F, Ulman YI, Hoffmann OI, Kitsara M, Kostomitsopoulos N, Neuhaus W, Petit-Demouliere B, Pollo S, Riso B, Schober S, Sotiropoulos A, Thomas A, Vitale A, Wilflingseder D, Ahluwalia A, 2024. Toward a common interpretation of the 3Rs principles in animal research. Lab. Anim. 53 (12), 347–350. 10.1038/s41684-024-01476-2 PMC11599037 39548348

[R38] Lei L, Zhu B, Qiao K, Zhou Y, Chen X, Men J, Yang L, Wang Q, Han J, Zhou B, 2022. New evidence for neurobehavioral toxicity of deltamethrin at environmentally relevant levels in zebrafish. Sci. Total. Environ. 822, 153623. 10.1016/j.scitotenv.2022.153623 35124052

[R39] Lubin A, Otterstrom J, Hoade Y, Bjedov I, Stead E, Whelan M, Gestri G, Paran Y, Payne E, 2021. A versatile, automated and high-throughput drug screening platform for zebrafish embryos. Biol. Open. 10 (9). 10.1242/bio.058513 PMC8430230 34472582

[R40] Maass C, Schaller S, Dallmann A, Bothe K, Müller D, 2023. Considering developmental neurotoxicity in vitro data for human health risk assessment using physiologically-based kinetic modeling: deltamethrin case study. Toxicol. Sci. 192 (1), 59–70. 36637193 10.1093/toxsci/kfad007 PMC10025876

[R41] MacPhail RC, Brooks J, Hunter DL, Padnos B, Irons TD, Padilla S, 2009. Locomotion in larval zebrafish: Influence of time of day, lighting and ethanol. NeuroToxicology 30 (1), 52–58. 18952124 10.1016/j.neuro.2008.09.011

[R42] MacRae CA, Peterson RT, 2015. Zebrafish as tools for drug discovery. Nat. Rev. Drug. Discov. 14 (10), 721–731. 26361349 10.1038/nrd4627

[R43] Morash MG, Soanes KH, Achenbach JC, Ellis LD, 2022. Assessing the Morphological and Behavioral Toxicity of Catechol Using Larval Zebrafish. Int. J. Mol. Sci. 23 (14). 10.3390/ijms23147985 PMC9316700 35887331

[R44] NTP, N.T.P., 2018. Data Release: Developmental NeuroToxicity Data Integration and Visualization Enabling Resource (DNT-DIVER). National Toxicology Program. National Institute of Environmental Health Sciences (NIEHS), Research Triangle Park, NC, USA.

[R45] OECD, 2007. Test No. 426: Developmental Neurotoxicity Study. O. f. E. C.-o. a. Development, Paris.

[R46] OECD, 2023b. Initial Recommendations on Evaluation of Data from the Developmental Neurotoxicity (DNT) In-Vitro Testing Battery, 377. OECD Series on Testing and Assessment, Paris.

[R47] OECD, O. f E.C.-o a D., 2022c. Case study on the use of integrated approaches for testing and assessment for developmental neurotoxicity hazard characterisation of imidacloprid and the metabolite desnitro-imidacloprid. OECD Publishing, Paris, France.

[R48] OECD, O. f E.C.-o a D., 2022a. Case study for the integration of in vitro data in the developmental neurotoxicity hazard identification and characterisation using deltamethrin as a prototype chemical. OECD Publishing, Paris, France.

[R49] OECD, O. f E.C.-o a D., 2022b. Case study on the use of integrated approaches for testing and assessment for developmental neurotoxicity hazard characterisation of acetamiprid. OECD Publishing, Paris, France.

[R50] OECD, O. f E.C.-o a D., 2023a. Initial Recommendations on Evaluation of Data from the Developmental Neurotoxicity (DNT) In-Vitro Testing Battery. OECD Publishing, Paris, France.

[R51] Parichy DM, 2015. Advancing biology through a deeper understanding of zebrafish ecology and evolution. eLife 4, e05635. 10.7554/eLife.05635 PMC4373672 25807087

[R52] Paul Friedman K, Foster MJ, Pham LL, Feshuk M, Watford SM, Wambaugh JF, Judson RS, Setzer RW, Thomas RS, 2023. Reproducibility of organ-level effects in repeat dose animal studies. Comput. Toxicol. 28, 100287. 10.1016/j.comtox.2023.100287 PMC10659077 37990691

[R53] Rennekamp AJ, Peterson RT, 2015. 15 years of zebrafish chemical screening. Curr. Opin. Chem. Biol. 24, 58–70. 25461724 10.1016/j.cbpa.2014.10.025 PMC4339096

[R54] Sachana M, Shafer TJ, Terron A, 2021. Toward a Better Testing Paradigm for Developmental Neurotoxicity: OECD Efforts and Regulatory Considerations. Biol. (Basel) 10 (2). 10.3390/biology10020086 PMC7912397 33498772

[R55] Sakai C, Ijaz S, Hoffman EJ, 2018. Zebrafish Models of Neurodevelopmental Disorders: Past, Present, and Future. Front. Mol. Neurosci. 11, 2018. 10.3389/fnmol.2018.00294 PMC6123572 30210288

[R56] Sedykh A, 2016. CurveP Method for Rendering High-Throughput Screening Dose-Response Data into Digital Fingerprints. Methods Mol. Biol. 1473, 135–141. 27518631 10.1007/978-1-4939-6346-1_14

[R57] Sedykh A, Zhu H, Tang H, Zhang L, Richard A, Rusyn I, Tropsha A, 2011. Use of in vitro HTS-derived concentration-response data as biological descriptors improves the accuracy of QSAR models of in vivo toxicity. Environ. Health Perspect. 119 (3), 364–370. 20980217 10.1289/ehp.1002476 PMC3060000

[R58] Sewell F, Alexander-White C, Brescia S, Currie RA, Roberts R, Roper C, Vickers C, Westmoreland C, Kimber I, 2024. New approach methodologies (NAMs): identifying and overcoming hurdles to accelerated adoption. Toxicol. Res. (Camb. ) 13 (2), tfae044. 10.1093/toxres/tfae044 PMC10964841 38533179

[R59] Spath J, Raab J, Schor J, Scholz S, Gutsfeld S, Tal T, 2026. The zebrafish visual and acoustic motor response (VAMR) assay has the potential to add value to the developmental neurotoxicity in Vitro battery (DNT IVB). NeuroToxicology 114, 103414. 10.1016/j.neuro.2026.103414 41780647

[R60] Strähle U, Scholz S, Geisler R, Greiner P, Hollert H, Rastegar S, Schumacher A, Selderslaghs I, Weiss C, Witters H, Braunbeck T, 2012. Zebrafish embryos as an alternative to animal experiments–a commentary on the definition of the onset of protected life stages in animal welfare regulations. Reprod. Toxicol. 33 (2), 128–132. 21726626 10.1016/j.reprotox.2011.06.121

[R61] Sutha J, Gayathri M, Ramesh M, 2024. Chronic exposure to tris (2-chloroethyl) phosphate (TCEP) induces brain structural and functional changes in zebrafish (Danio rerio): A comparative study on the environmental and LC50 concentrations of TCEP. Environ. Sci. Pollut. Res. Int. 31 (11), 16770–16781. 38321284 10.1007/s11356-024-32154-y

[R62] Takai Y, Tokusumi H, Sato M, Inoue D, Chen K, Takamura T, Enoki S, Ueno Y, Kang IJ, Shimasaki Y, Qiu X, Oshima Y, 2022. Combined effect of diazepam and polystyrene microplastics on the social behavior of medaka (Oryzias latipes). Chemosphere 299, 134403. 10.1016/j.chemosphere.2022.134403 35341767

[R63] Teame T, Zhang Z, Ran C, Zhang H, Yang Y, Ding Q, Xie M, Gao C, Ye Y, Duan M, Zhou Z, 2019. The use of zebrafish (Danio rerio) as biomedical models. Anim. Front. 9 (3), 68–77. 32002264 10.1093/af/vfz020 PMC6951987

[R64] Ton C, Lin Y, Willett C, 2006. Zebrafish as a model for developmental neurotoxicity testing. Birth Defects Res. A Clin. Mol. Teratol. 76 (7), 553–567. 16933308 10.1002/bdra.20281

[R65] Truong L, Reif DM, St Mary L, Geier MC, Truong HD, Tanguay RL, 2014. Multidimensional in vivo hazard assessment using zebrafish. Toxicol. Sci. 137 (1), 212–233. 24136191 10.1093/toxsci/kft235 PMC3871932

[R66] von Hellfeld R, Gade C, Baumann L, Leist M, Braunbeck T, 2023. The sensitivity of the zebrafish embryo coiling assay for the detection of neurotoxicity by compounds with diverse modes of action. Environ. Sci. Pollut. Res. Int. 30 (30), 75281–75299. 37213015 10.1007/s11356-023-27662-2 PMC10293418

[R67] Xie Z, Lu G, Yu Y, 2022a. Early-stage high-concentration thiacloprid exposure induced persistent behavioral alterations in zebrafish. Int. J. Environ. Res. Public. Health 19 (17). 10.3390/ijerph191710920 PMC9518391 36078631

[R68] Xie Z, Lu G, Zhou R, Ma Y, 2022b. Thiacloprid-induced hepatotoxicity in zebrafish: Activation of the extrinsic and intrinsic apoptosis pathways regulated by p53 signaling pathway. Aquat. Toxicol. 246, 106147. 10.1016/j.aquatox.2022.106147 35349858

[R69] Yuan S, Liang C, Li W, Letcher RJ, Liu C, 2021. A comprehensive system for detection of behavioral change of D. magna exposed to various chemicals. J. Hazard. Mater. 402, 123731. 10.1016/j.jhazmat.2020.123731 33254763

[R70] Zhang G, Truong L, Tanguay RL, Reif DM, 2017. A new statistical approach to characterize chemical-elicited behavioral effects in high-throughput studies using zebrafish. PLoS. One 12 (1), e0169408. 10.1371/journal.pone.0169408 PMC5242475 28099482

