# Supplementary material for: Zebrafish behavioral assessment as a first-tier whole-organism NAM for developmental neurotoxicity: A multi-laboratory evaluation
Source: Neurotoxicology. Author manuscript; Available in PMC 2026 Aug 15. (PMC13475518; doi:10.1016/j.neuro.2026.103504)

Tris(1,3−dichloro−2−propyl) phosphate

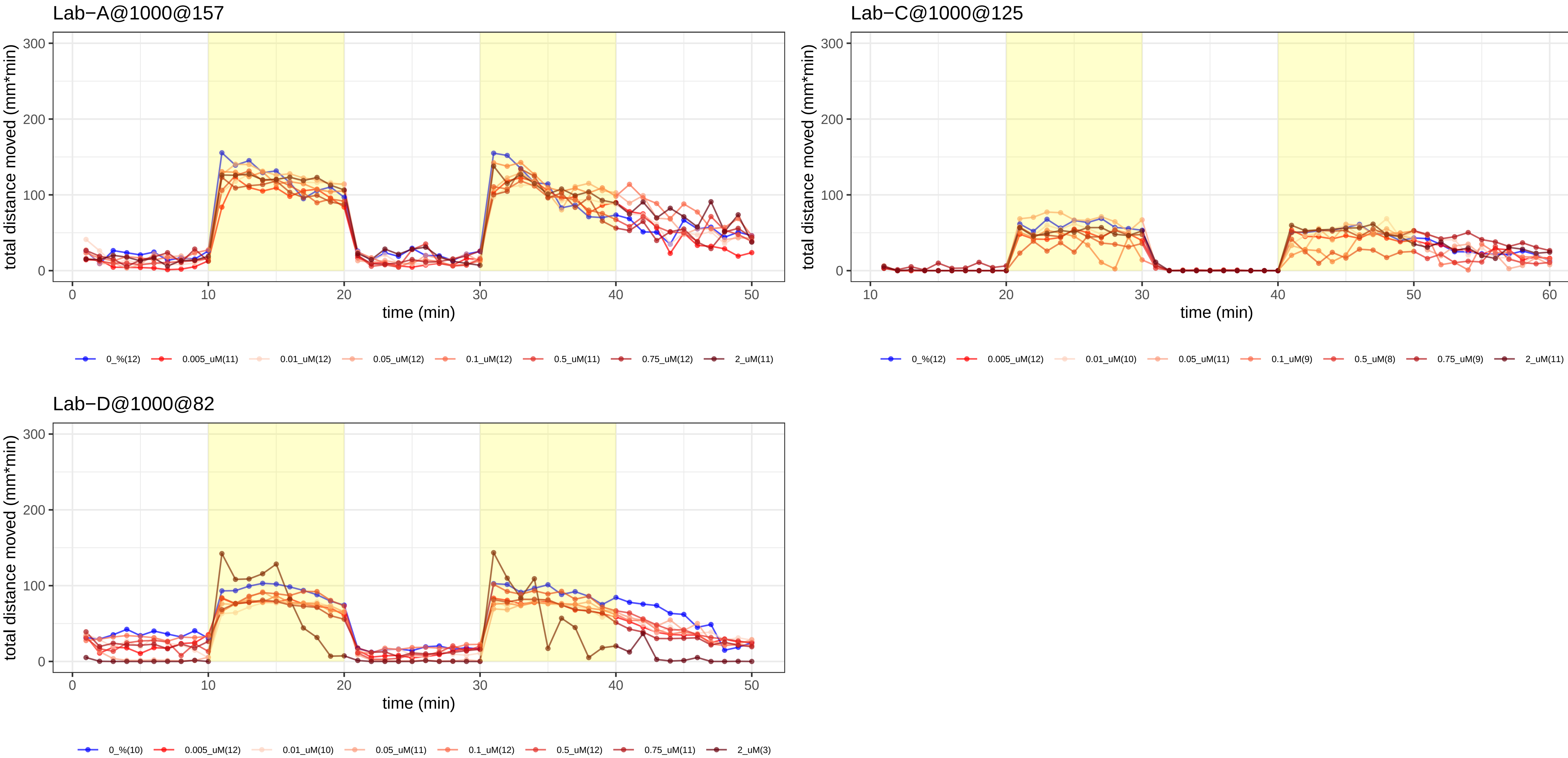

Acetamiprid

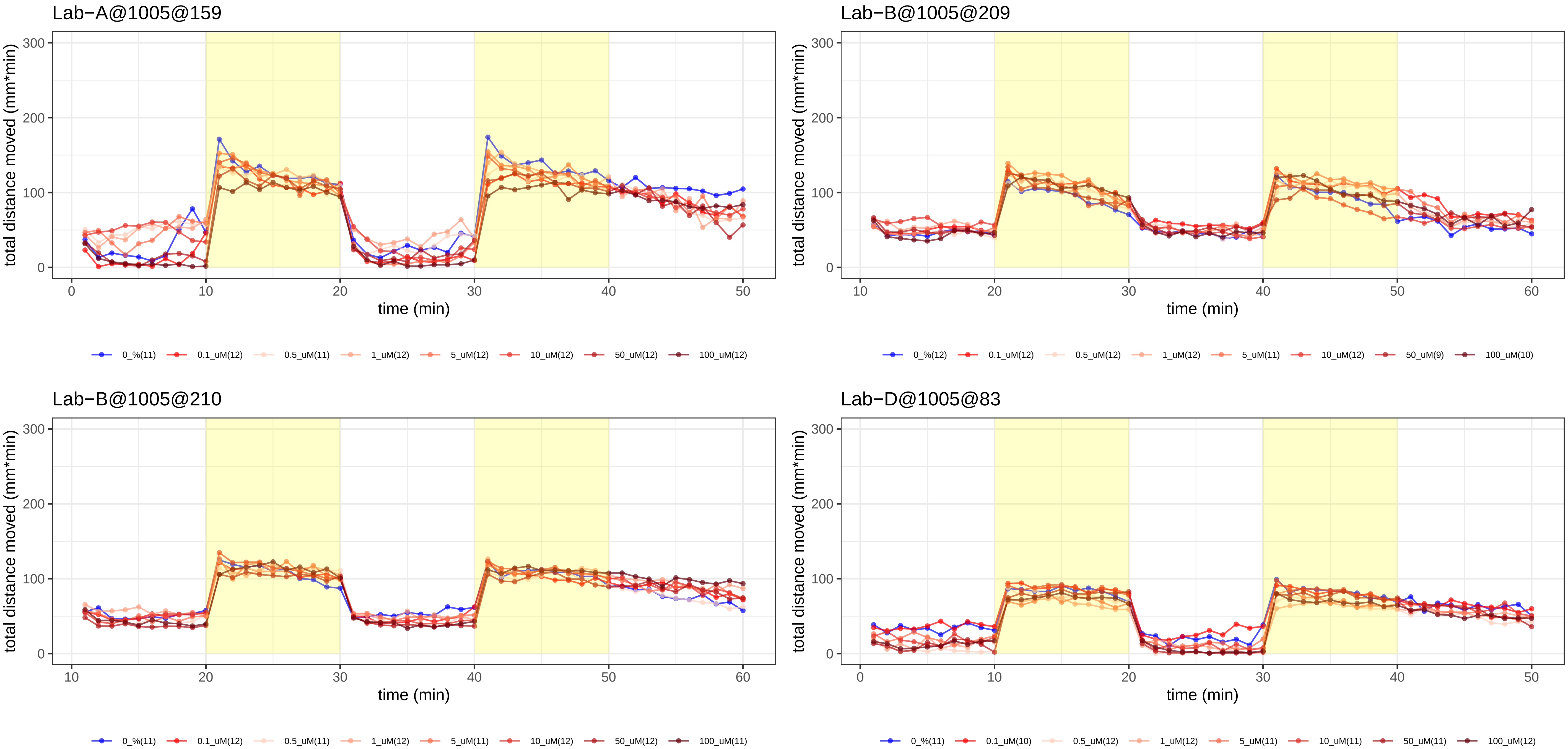

Cypermethrin

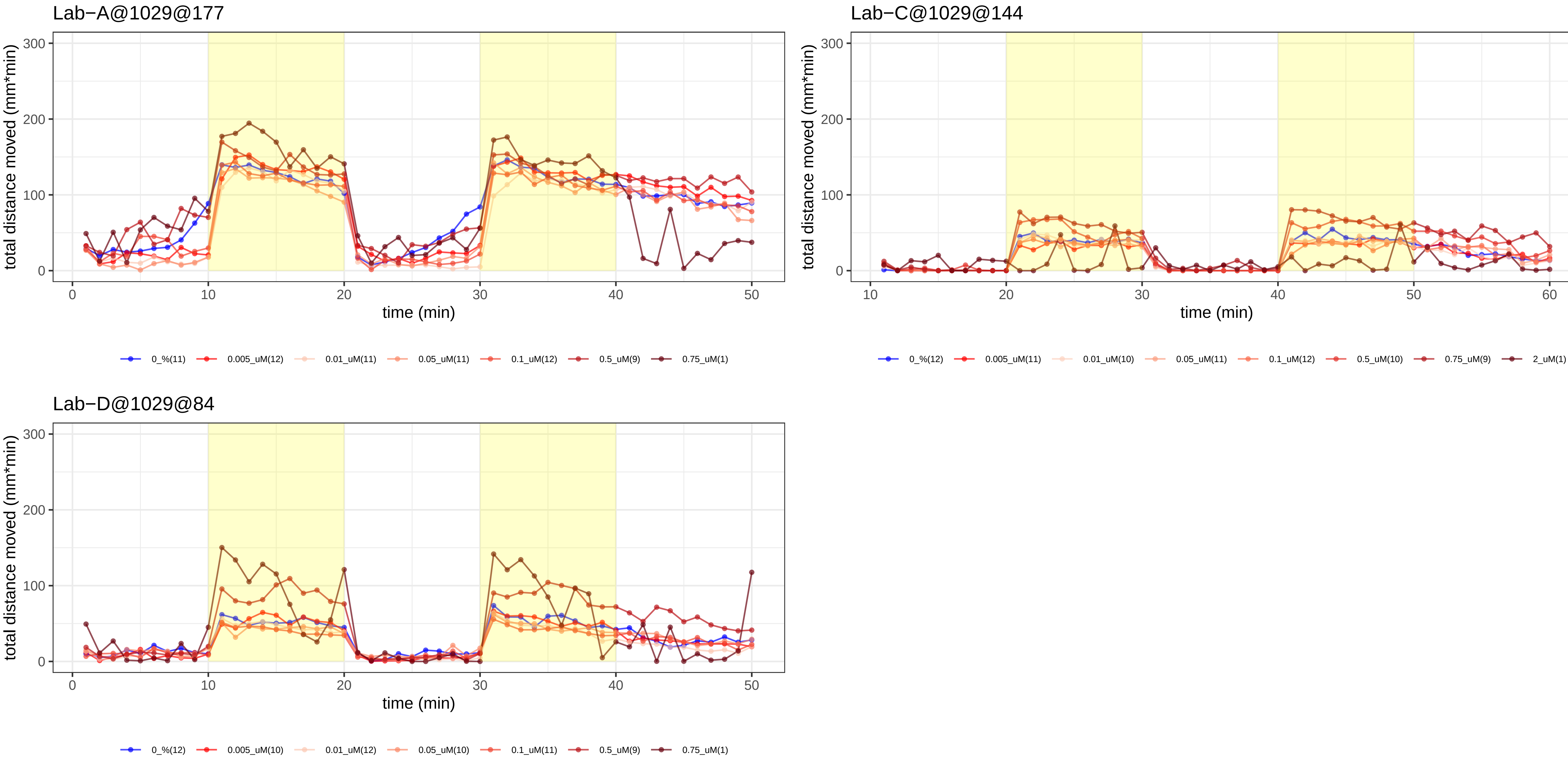

Triphenyl phosphates isopropylated

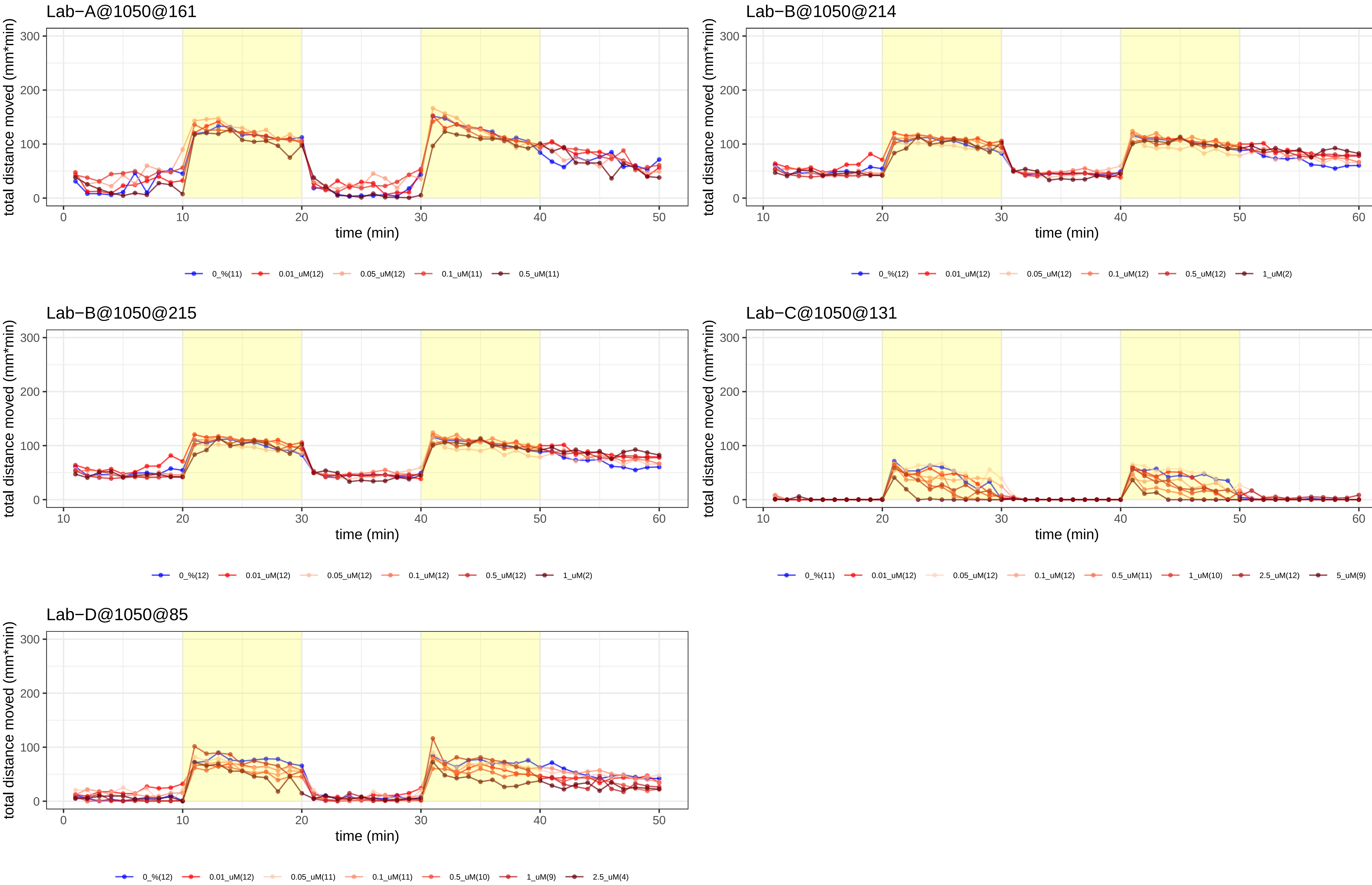

Tris(2−chloroisopropyl)phosphate

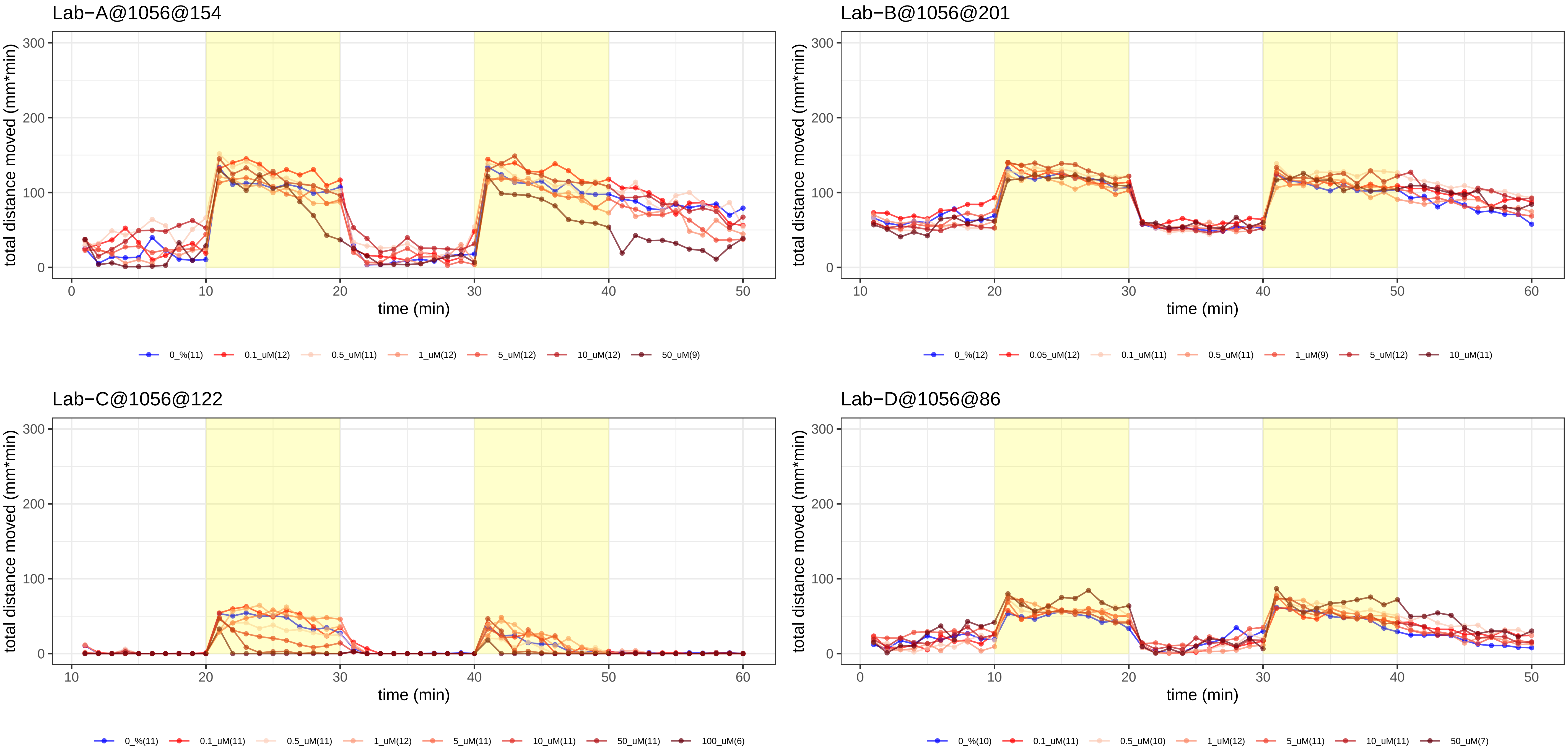

Diazinon

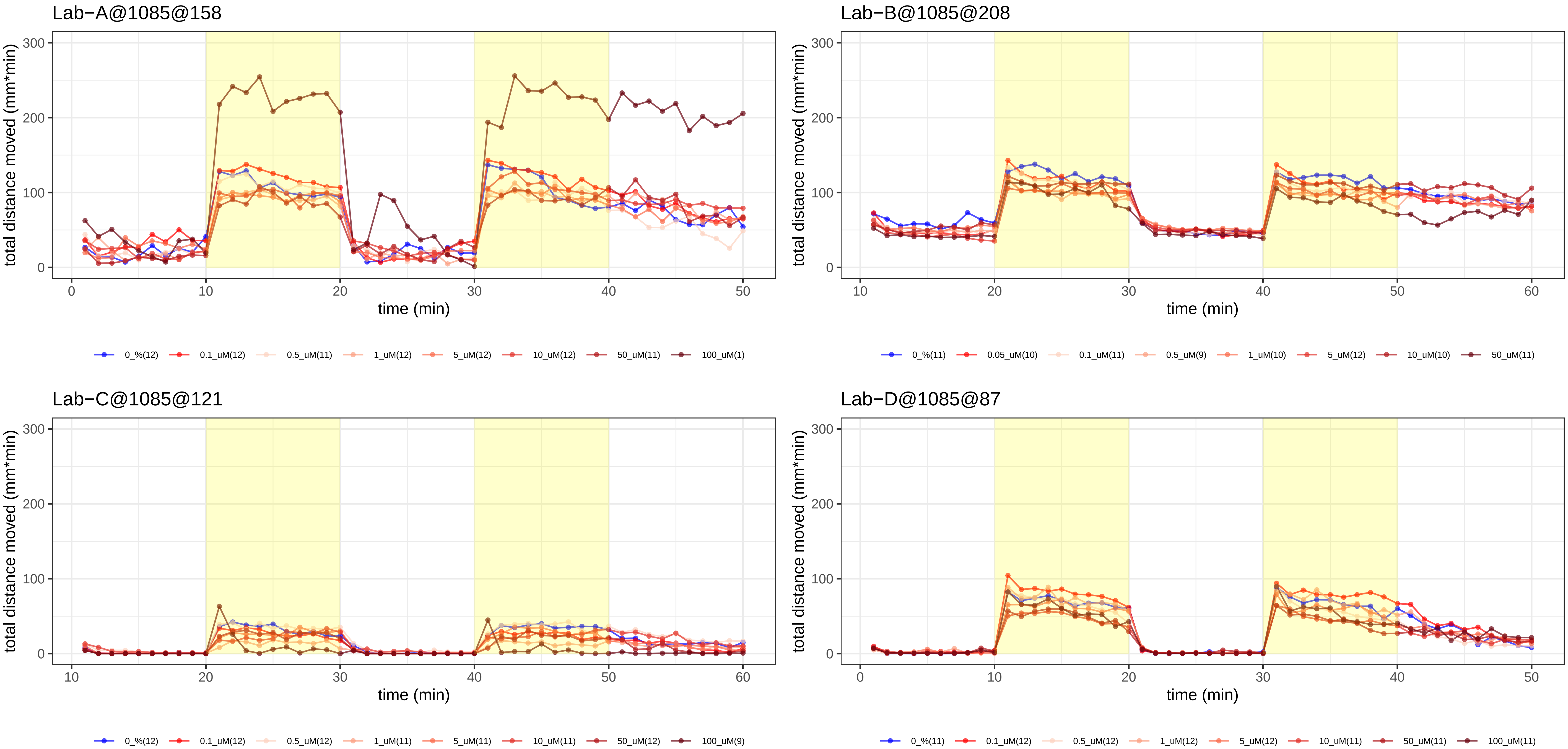

Lab−A@1085@158
Lab−B@1085@208
Lab−C@1085@121
Lab−D@1085@87
total distance moved (mm*min)
time (min)
0_%(12) 0.1_uM(12) 0.5_uM(11) 1_uM(12) 5_uM(12) 10_uM(12) 50_uM(11) 100_uM(1)
0_%(11) 0.05_uM(10) 0.1_uM(11) 0.5_uM(9) 1_uM(10) 5_uM(12) 10_uM(10) 50_uM(11)
0_%(12) 0.1_uM(12) 0.5_uM(12) 1_uM(11) 5_uM(11) 10_uM(11) 50_uM(12) 100_uM(9)
0_%(11) 0.1_uM(12) 0.5_uM(12) 1_uM(12) 5_uM(12) 10_uM(11) 50_uM(11) 100_uM(11)

Trichlorfon

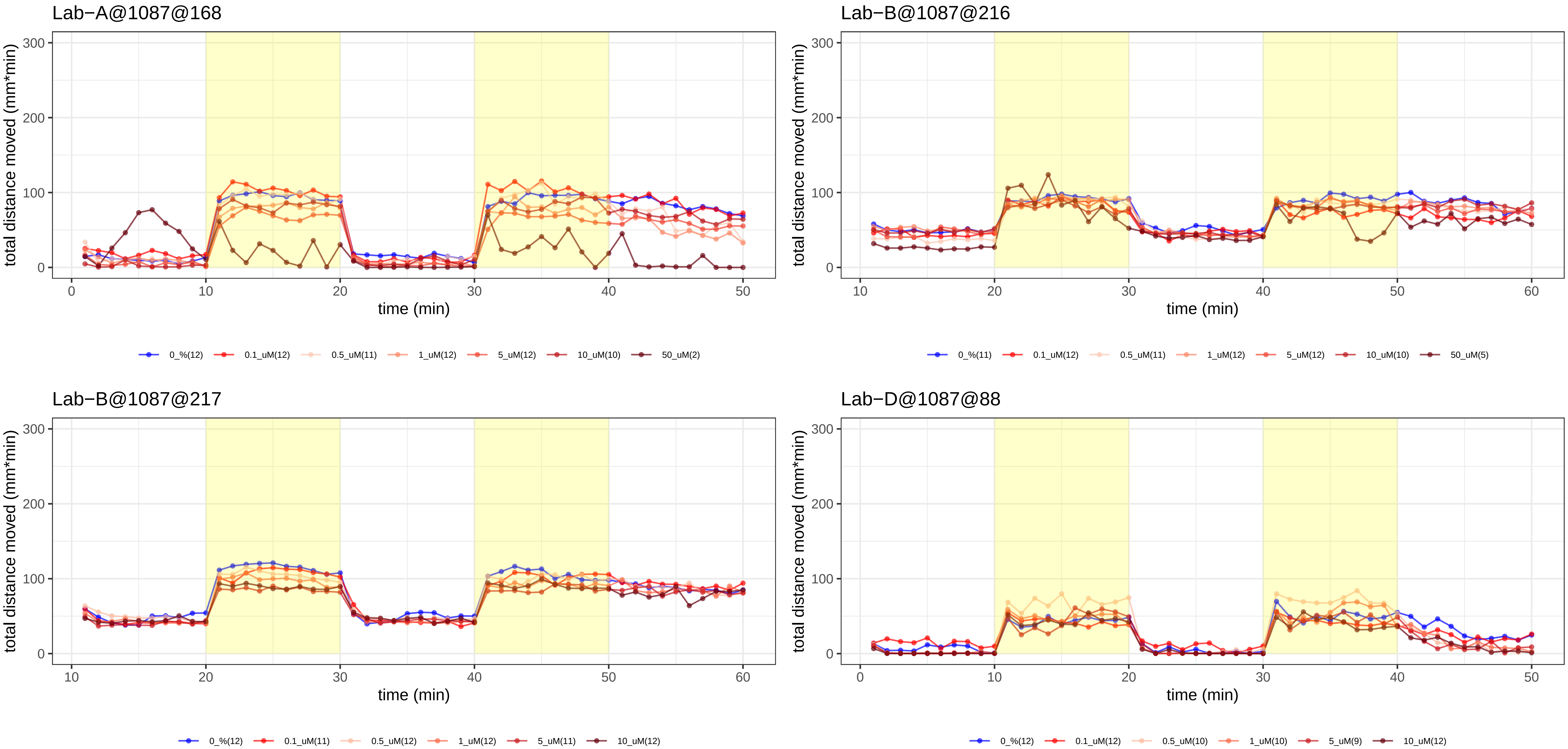

tert−Butylphenyl diphenyl phosphate

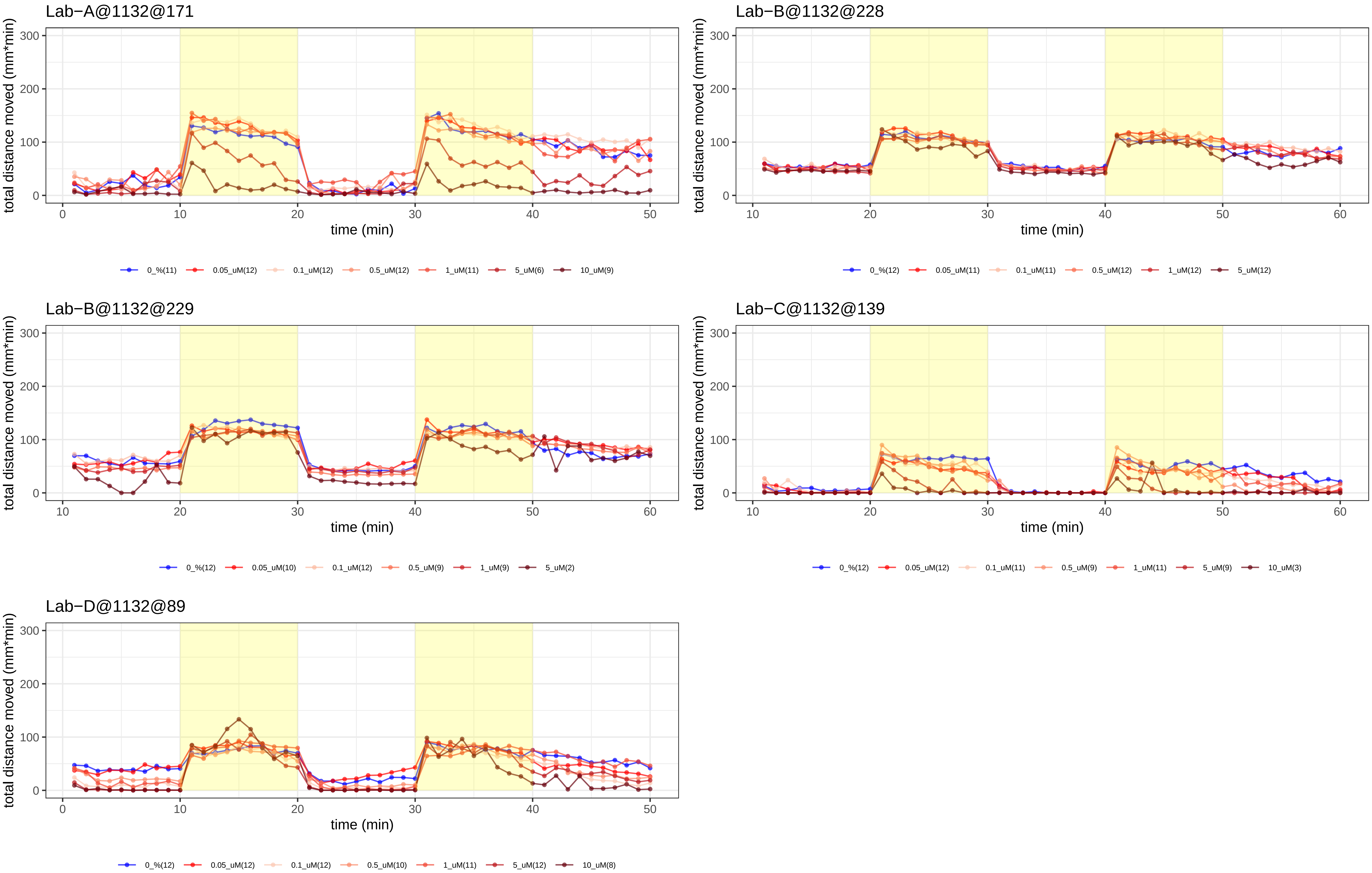

3,3',5,5'−Tetrabromobisphenol A

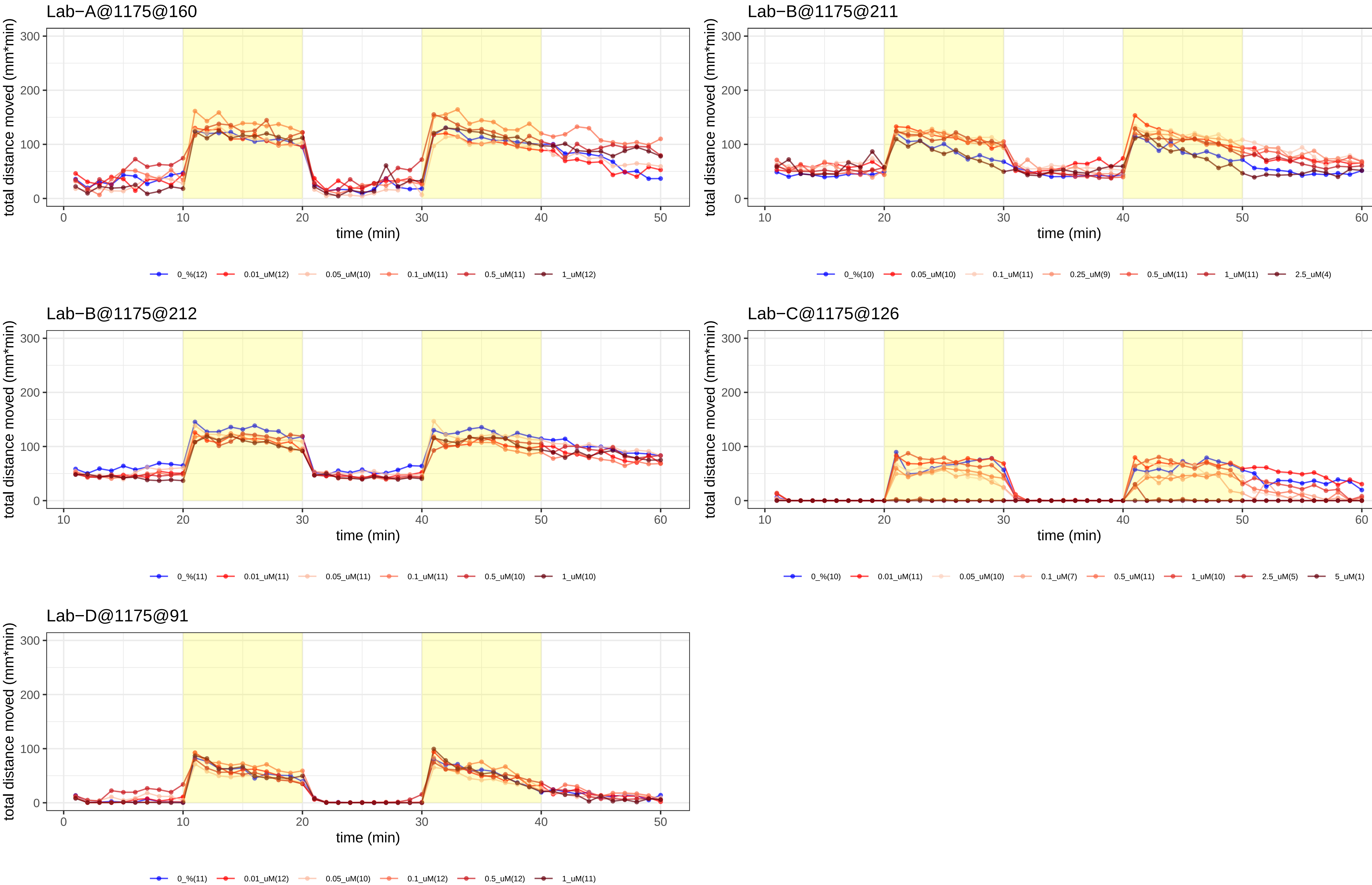

Tri−o−cresyl phosphate

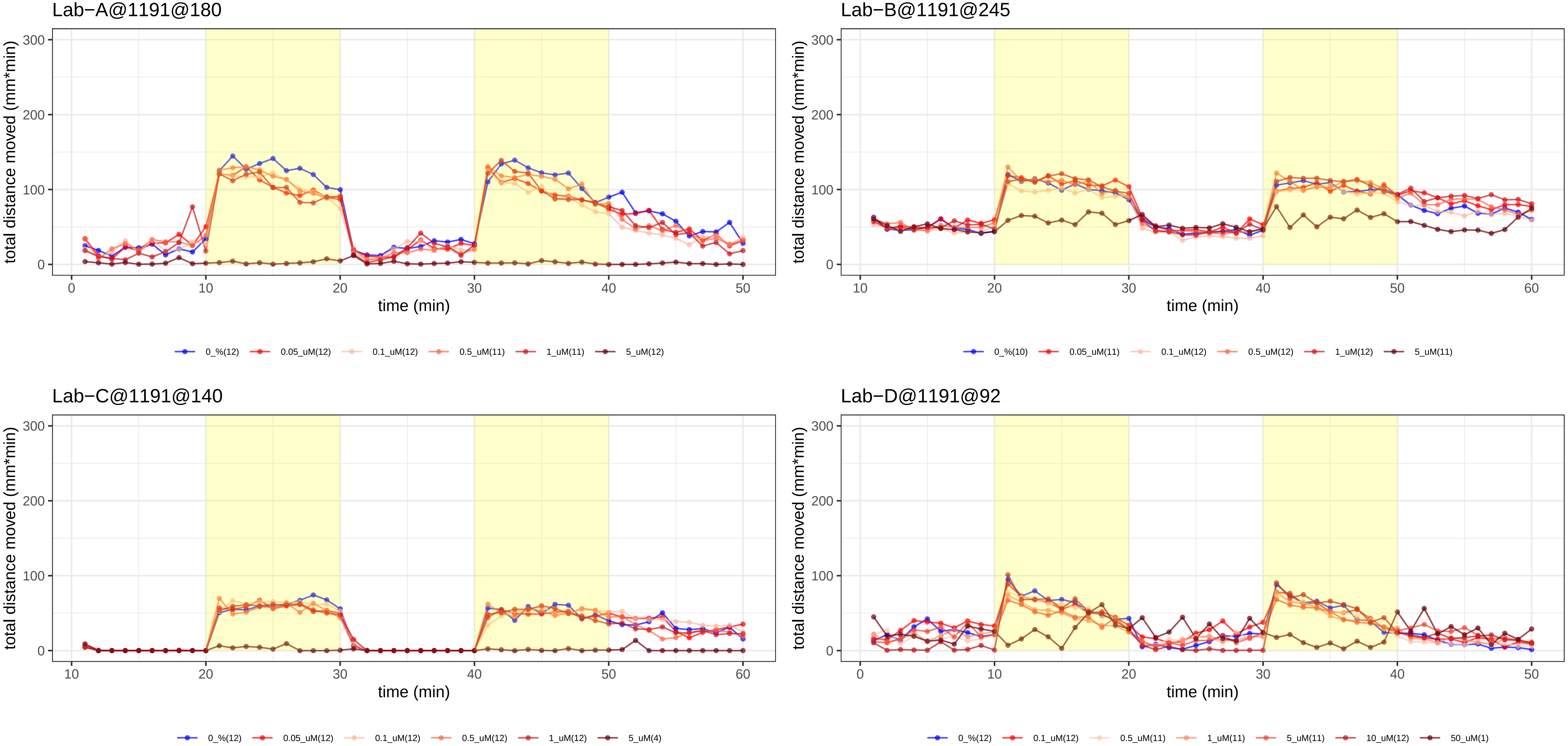

Chloramben

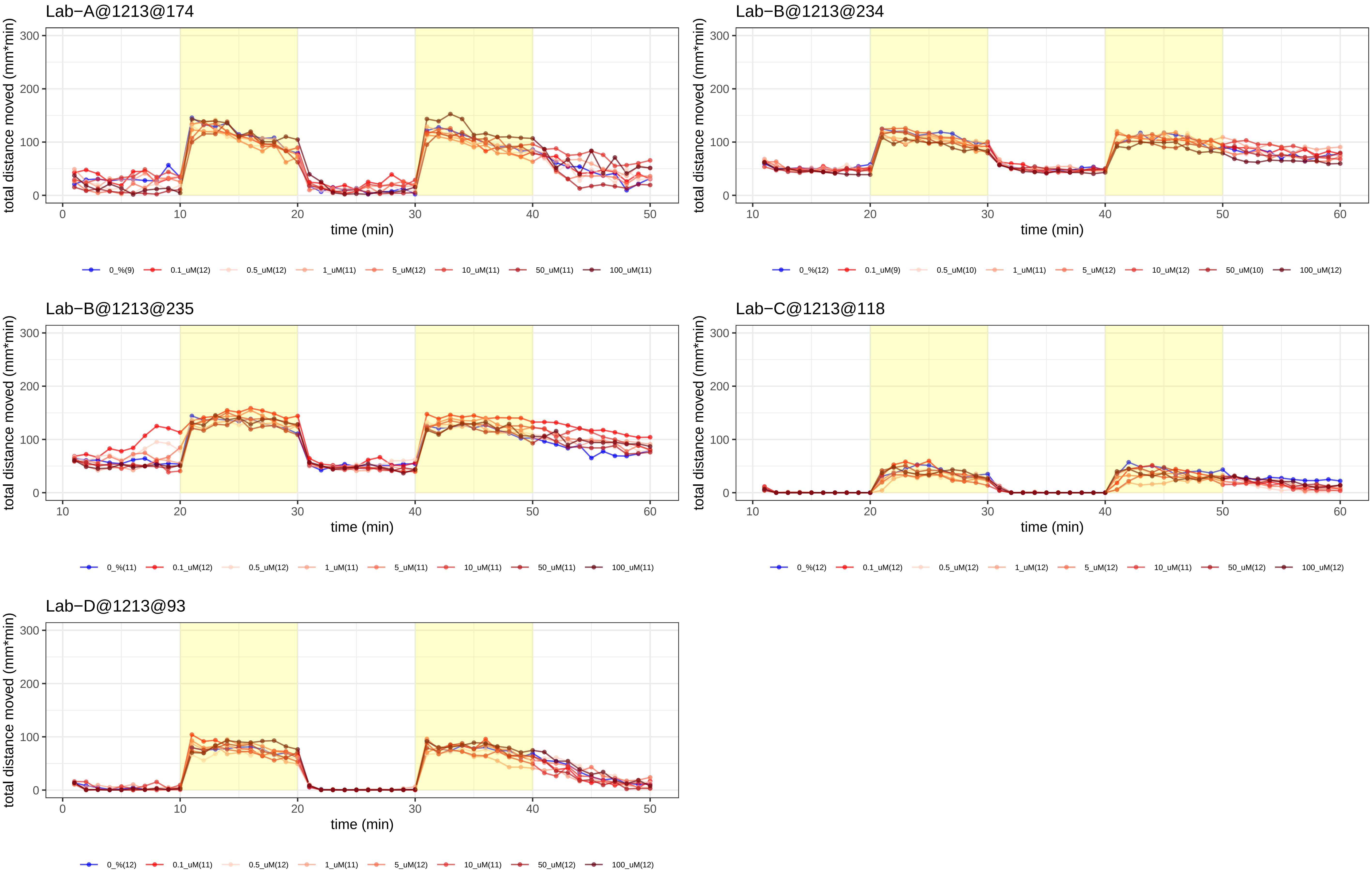

Lab−A@1213@174
total distance moved (mm*min)
time (min)
0_%(9)
0.1_uM(12)
0.5_uM(12)
1_uM(11)
5_uM(12)
10_uM(11)
50_uM(11)
100_uM(11)
Lab−B@1213@234
total distance moved (mm*min)
time (min)
0_%(12)
0.1_uM(9)
0.5_uM(10)
1_uM(11)
5_uM(12)
10_uM(12)
50_uM(10)
100_uM(12)
Lab−B@1213@235
total distance moved (mm*min)
time (min)
0_%(11)
0.1_uM(12)
0.5_uM(12)
1_uM(11)
5_uM(11)
10_uM(11)
50_uM(11)
100_uM(11)
Lab−C@1213@118
total distance moved (mm*min)
time (min)
0_%(12)
0.1_uM(12)
0.5_uM(12)
1_uM(12)
5_uM(12)
10_uM(11)
50_uM(12)
100_uM(12)
Lab−D@1213@93
total distance moved (mm*min)
time (min)
0_%(12)
0.1_uM(11)
0.5_uM(12)
1_uM(11)
5_uM(12)
10_uM(11)
50_uM(12)
100_uM(12)

Dimethoate

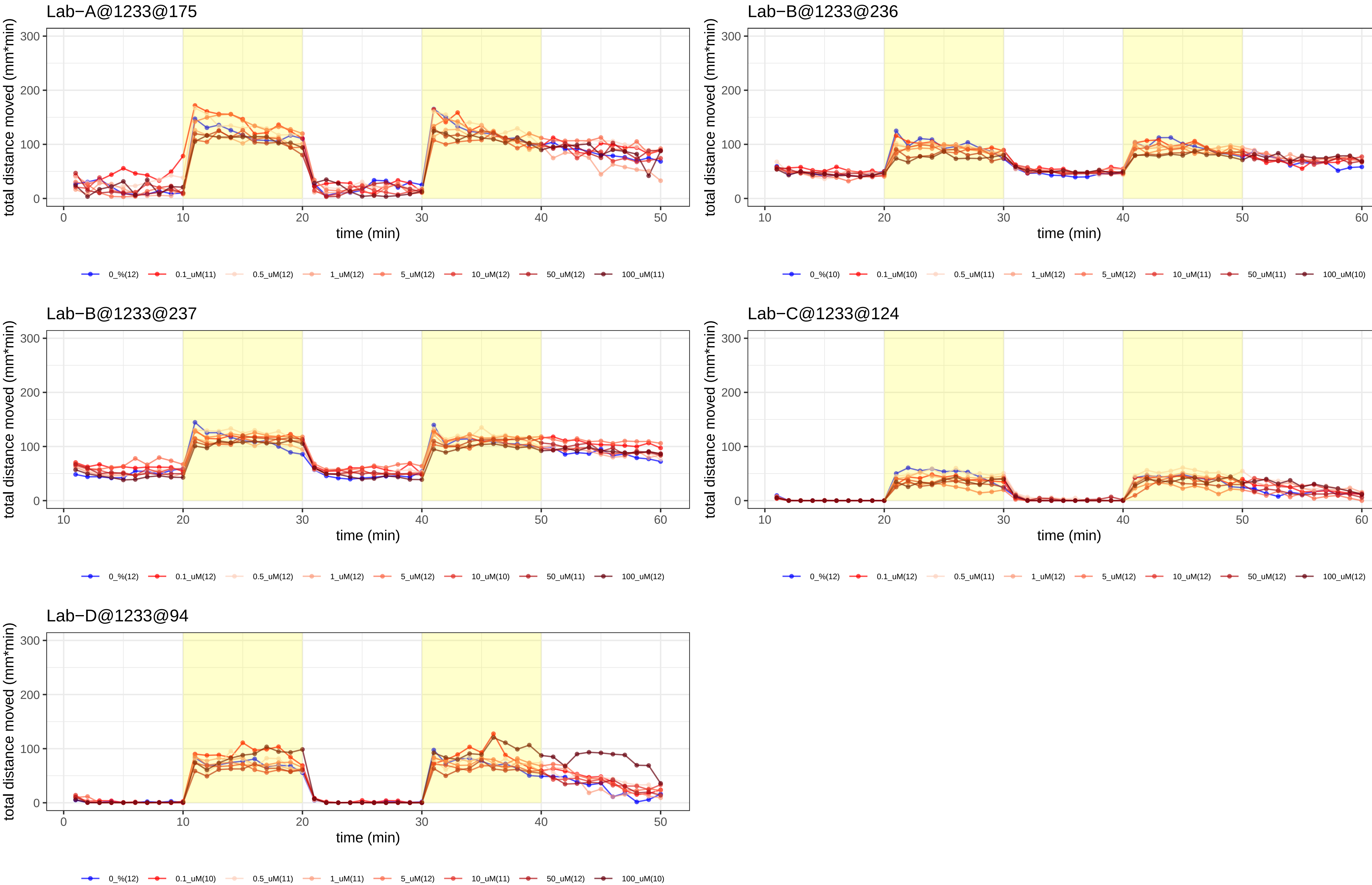

Lab−A@1233@175
Lab−B@1233@236
Lab−B@1233@237
Lab−C@1233@124
Lab−D@1233@94
total distance moved (mm*min)
time (min)
0_%(12) 0.1_uM(11) 0.5_uM(12) 1_uM(12) 5_uM(12) 10_uM(12) 50_uM(12) 100_uM(11)
0_%(10) 0.1_uM(10) 0.5_uM(11) 1_uM(12) 5_uM(12) 10_uM(11) 50_uM(11) 100_uM(10)
0_%(12) 0.1_uM(12) 0.5_uM(12) 1_uM(12) 5_uM(12) 10_uM(10) 50_uM(11) 100_uM(12)
0_%(12) 0.1_uM(12) 0.5_uM(11) 1_uM(12) 5_uM(12) 10_uM(12) 50_uM(12) 100_uM(12)
0_%(12) 0.1_uM(10) 0.5_uM(11) 1_uM(11) 5_uM(12) 10_uM(11) 50_uM(12) 100_uM(10)

Heptachlor

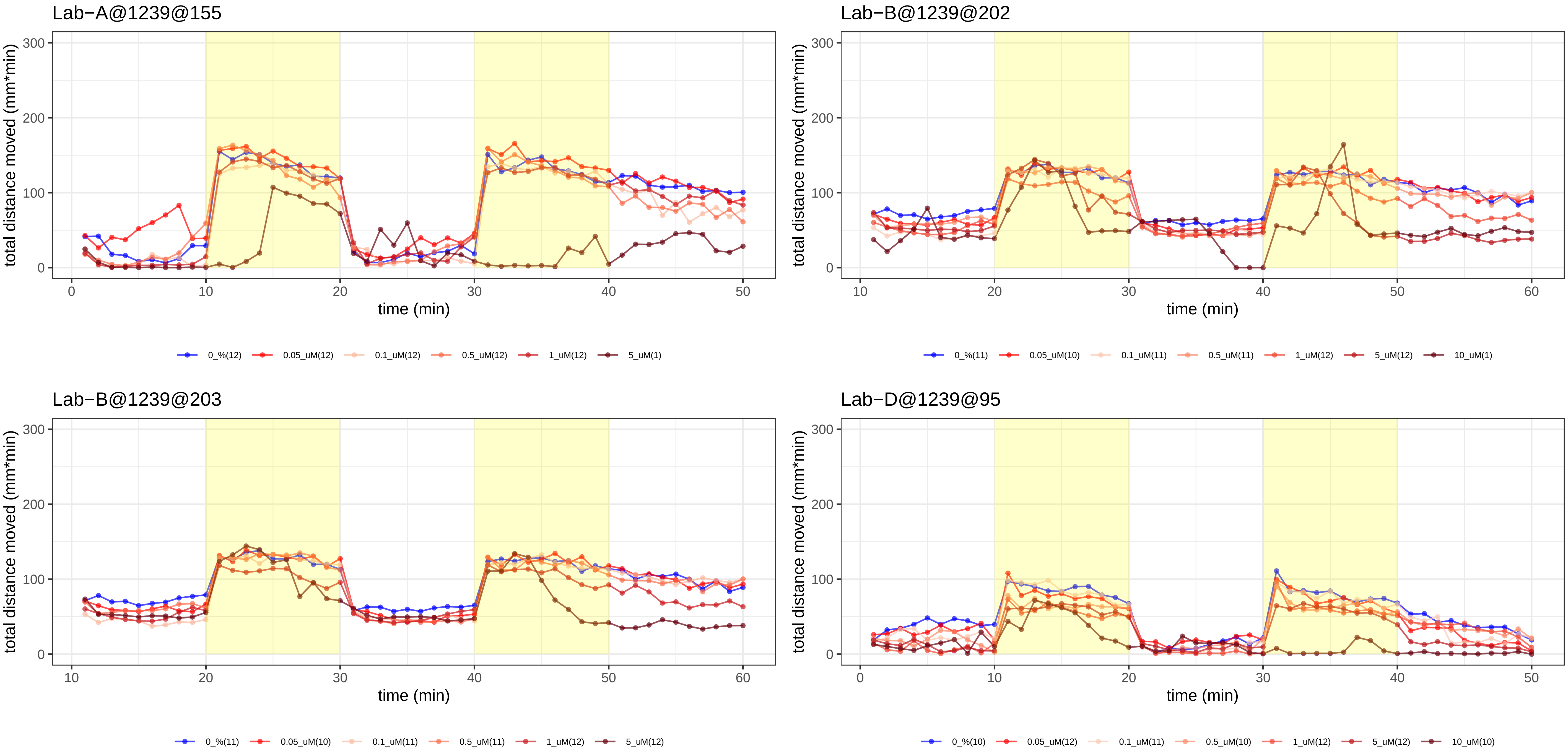

Lab−A@1239@155
Lab−B@1239@202
Lab−B@1239@203
Lab−D@1239@95
total distance moved (mm*min)
time (min)
0_%(12)
0.05_uM(12)
0.1_uM(12)
0.5_uM(12)
1_uM(12)
5_uM(1)
0_%(11)
0.05_uM(10)
0.1_uM(11)
0.5_uM(11)
1_uM(12)
5_uM(12)
10_uM(1)
0_%(11)
0.05_uM(10)
0.1_uM(11)
0.5_uM(11)
1_uM(12)
5_uM(12)
0_%(10)
0.05_uM(12)
0.1_uM(11)
0.5_uM(10)
1_uM(12)
5_uM(12)
10_uM(10)

2−Ethylhexyl diphenyl phosphate

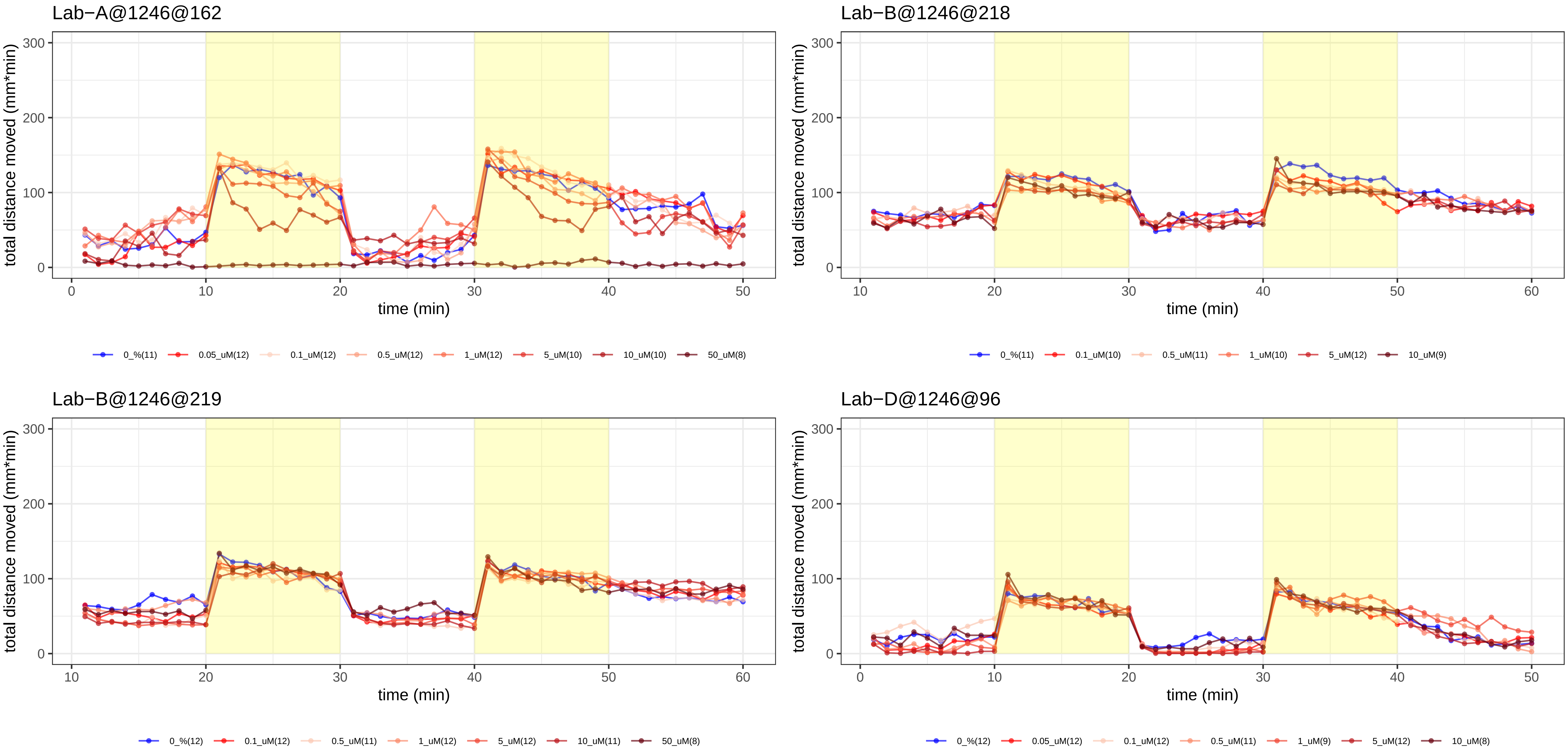

Tris(methylphenyl) phosphate

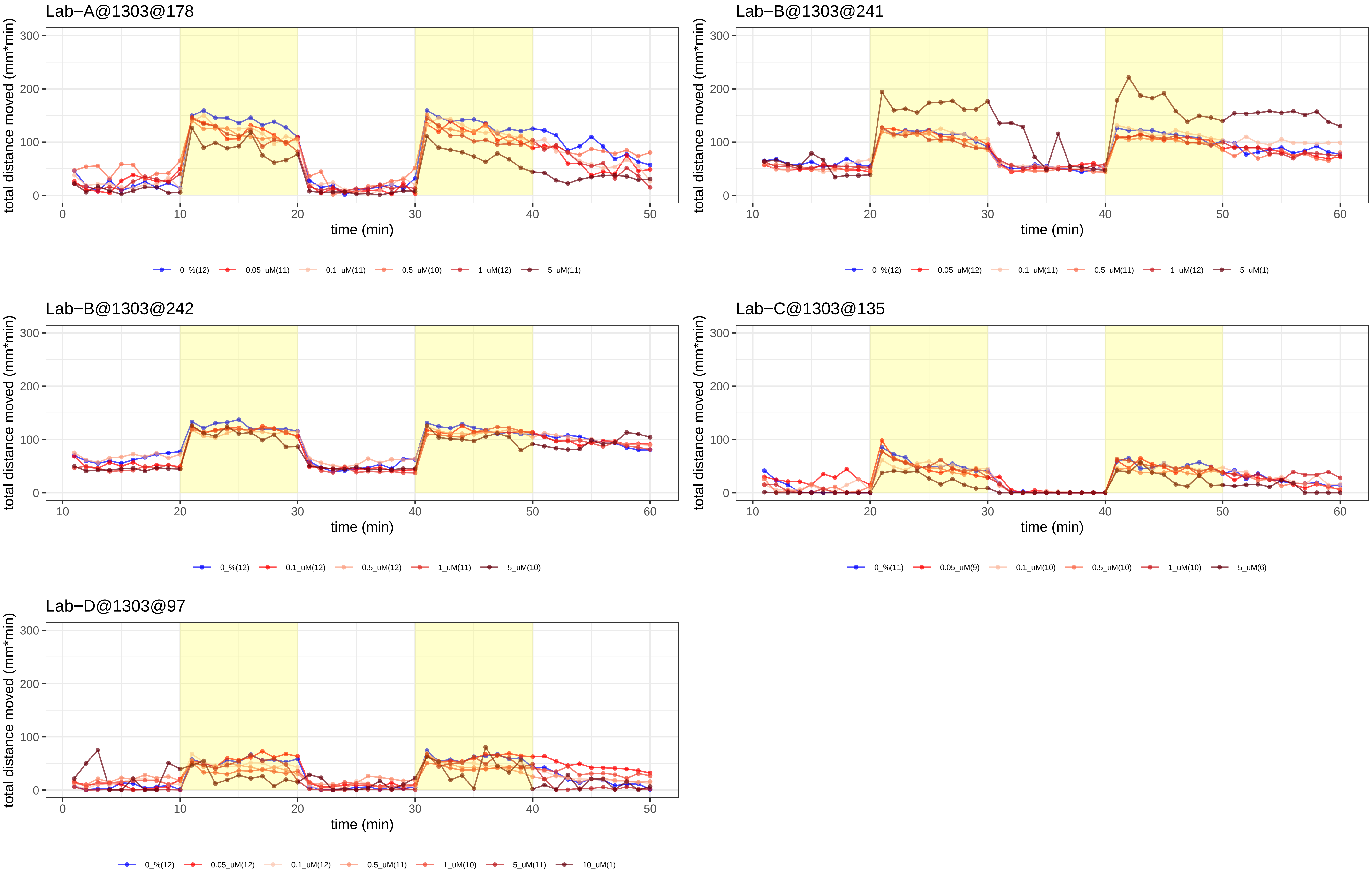

Aldicarb

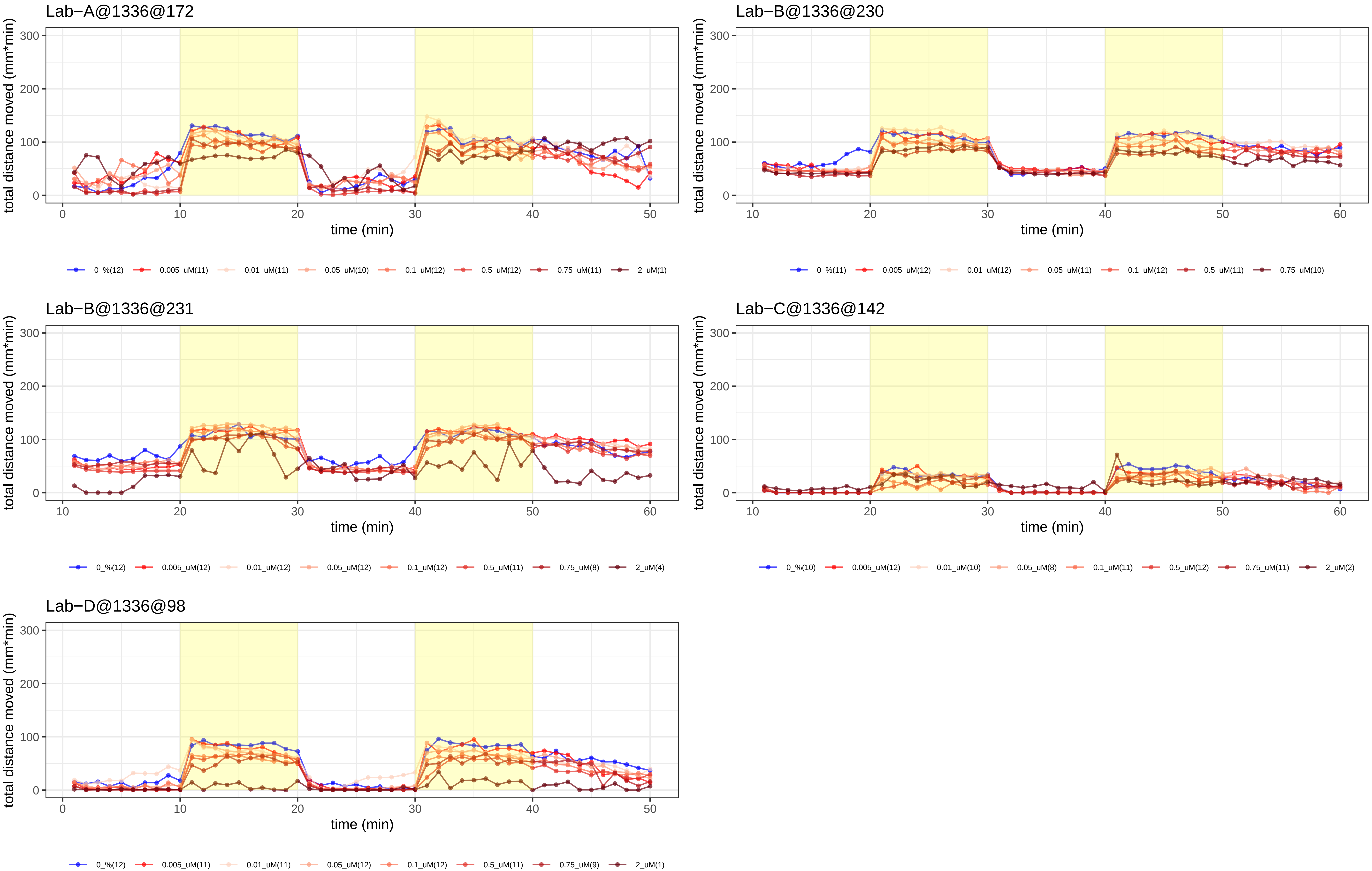

Lab−A@1336@172
Lab−B@1336@230
Lab−B@1336@231
Lab−C@1336@142
Lab−D@1336@98
total distance moved (mm*min)
time (min)
0_%(12)
0.005_uM(11)
0.01_uM(11)
0.05_uM(10)
0.1_uM(12)
0.5_uM(12)
0.75_uM(11)
2_uM(1)
0_%(11)
0.005_uM(12)
0.01_uM(12)
0.05_uM(11)
0.1_uM(12)
0.5_uM(11)
0.75_uM(10)
0_%(12)
0.005_uM(12)
0.01_uM(12)
0.05_uM(12)
0.1_uM(12)
0.5_uM(11)
0.75_uM(8)
2_uM(4)
0_%(10)
0.005_uM(12)
0.01_uM(10)
0.05_uM(8)
0.1_uM(11)
0.5_uM(12)
0.75_uM(11)
2_uM(2)
0_%(12)
0.005_uM(11)
0.01_uM(11)
0.05_uM(12)
0.1_uM(12)
0.5_uM(11)
0.75_uM(9)
2_uM(1)

Permethrin

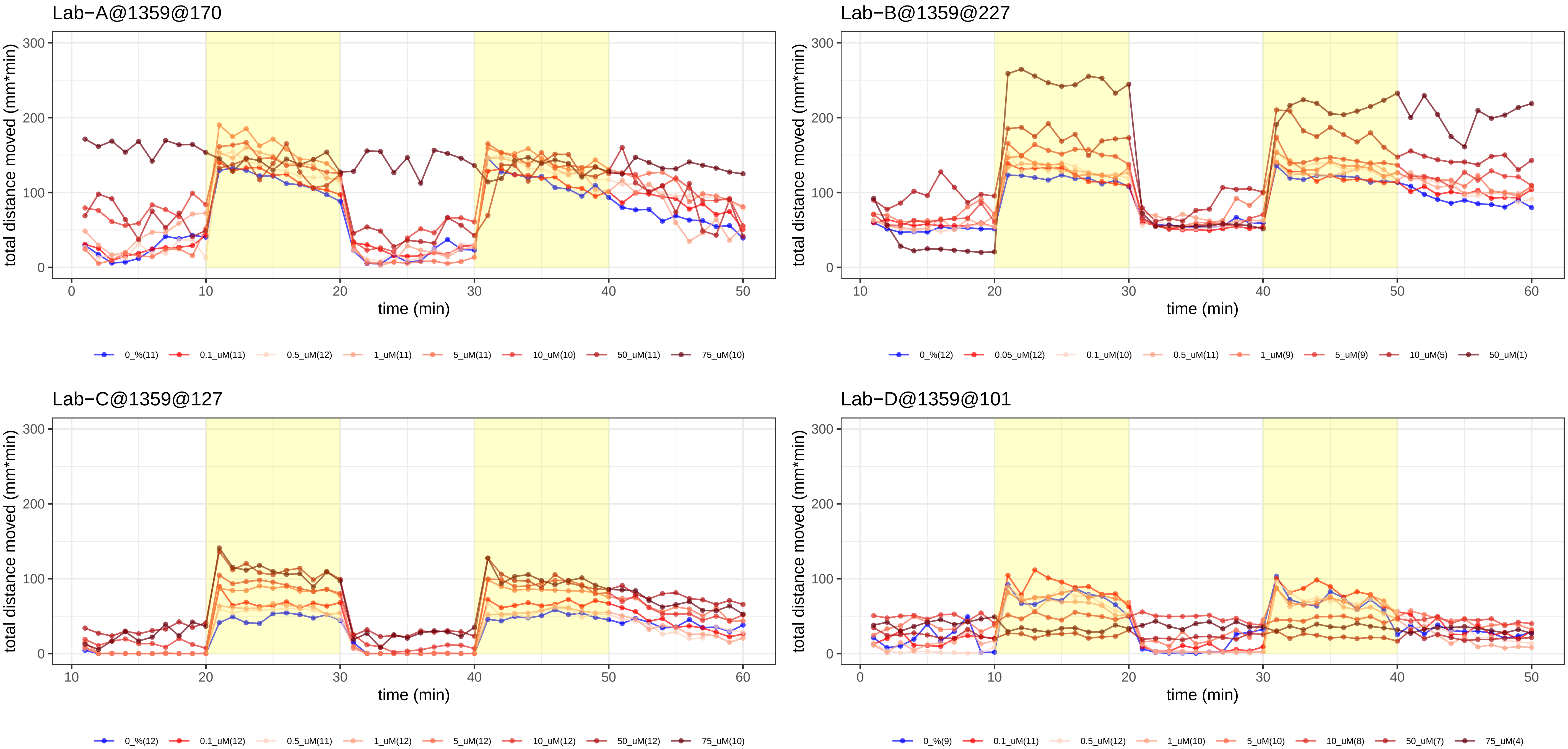

Lab−A@1359@170
total distance moved (mm*min)
time (min)
0_%(11)
0.1_uM(11)
0.5_uM(12)
1_uM(11)
5_uM(11)
10_uM(10)
50_uM(11)
75_uM(10)
Lab−B@1359@227
total distance moved (mm*min)
time (min)
0_%(12)
0.05_uM(12)
0.1_uM(10)
0.5_uM(11)
1_uM(9)
5_uM(9)
10_uM(5)
50_uM(1)
Lab−C@1359@127
total distance moved (mm*min)
time (min)
0_%(12)
0.1_uM(12)
0.5_uM(11)
1_uM(12)
5_uM(12)
10_uM(12)
50_uM(12)
75_uM(10)
Lab−D@1359@101
total distance moved (mm*min)
time (min)
0_%(9)
0.1_uM(11)
0.5_uM(12)
1_uM(10)
5_uM(10)
10_uM(8)
50_uM(7)
75_uM(4)

Allethrin

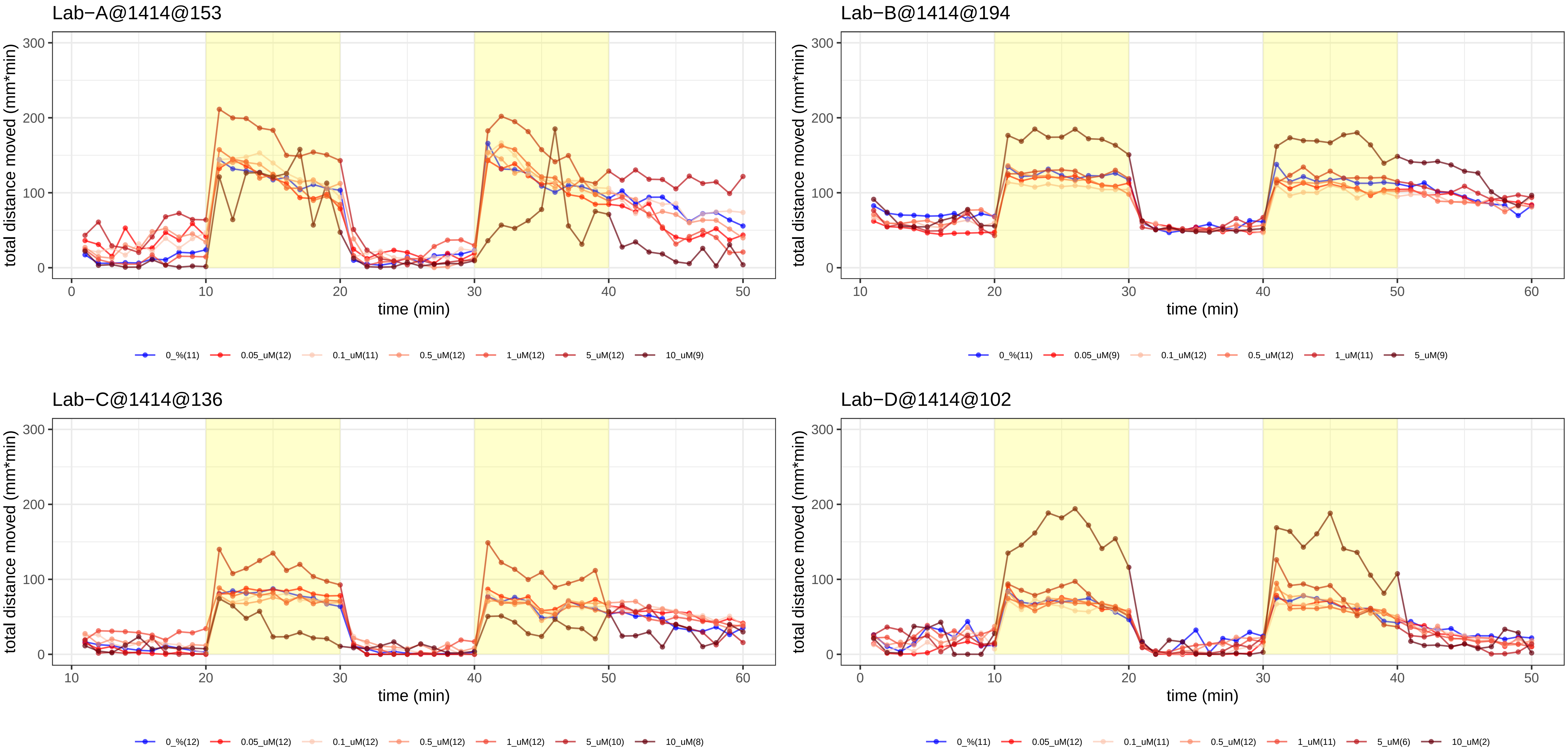
Lab−A@1414@153
Lab−B@1414@194
Lab−C@1414@136
Lab−D@1414@102
total distance moved (mm*min)
time (min)
0_%(11)
0.05_uM(12)
0.1_uM(11)
0.5_uM(12)
1_uM(12)
5_uM(12)
10_uM(9)
0_%(11)
0.05_uM(9)
0.1_uM(12)
0.5_uM(12)
1_uM(11)
5_uM(9)
0_%(12)
0.05_uM(12)
0.1_uM(12)
0.5_uM(12)
1_uM(12)
5_uM(10)
10_uM(8)
0_%(11)
0.05_uM(12)
0.1_uM(11)
0.5_uM(12)
1_uM(11)
5_uM(6)
10_uM(2)

Tris(2−chloroethyl) phosphate

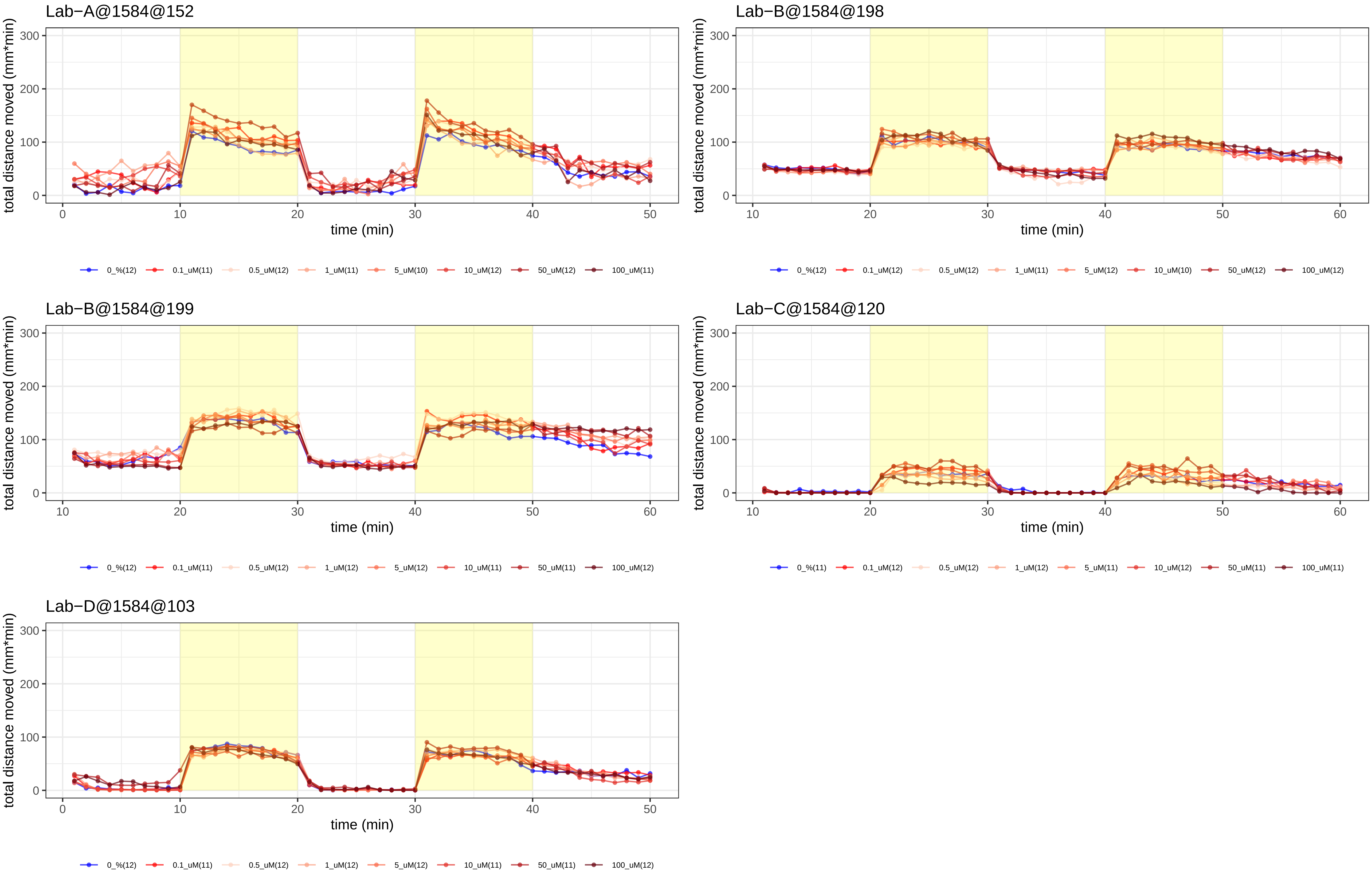

Dieldrin

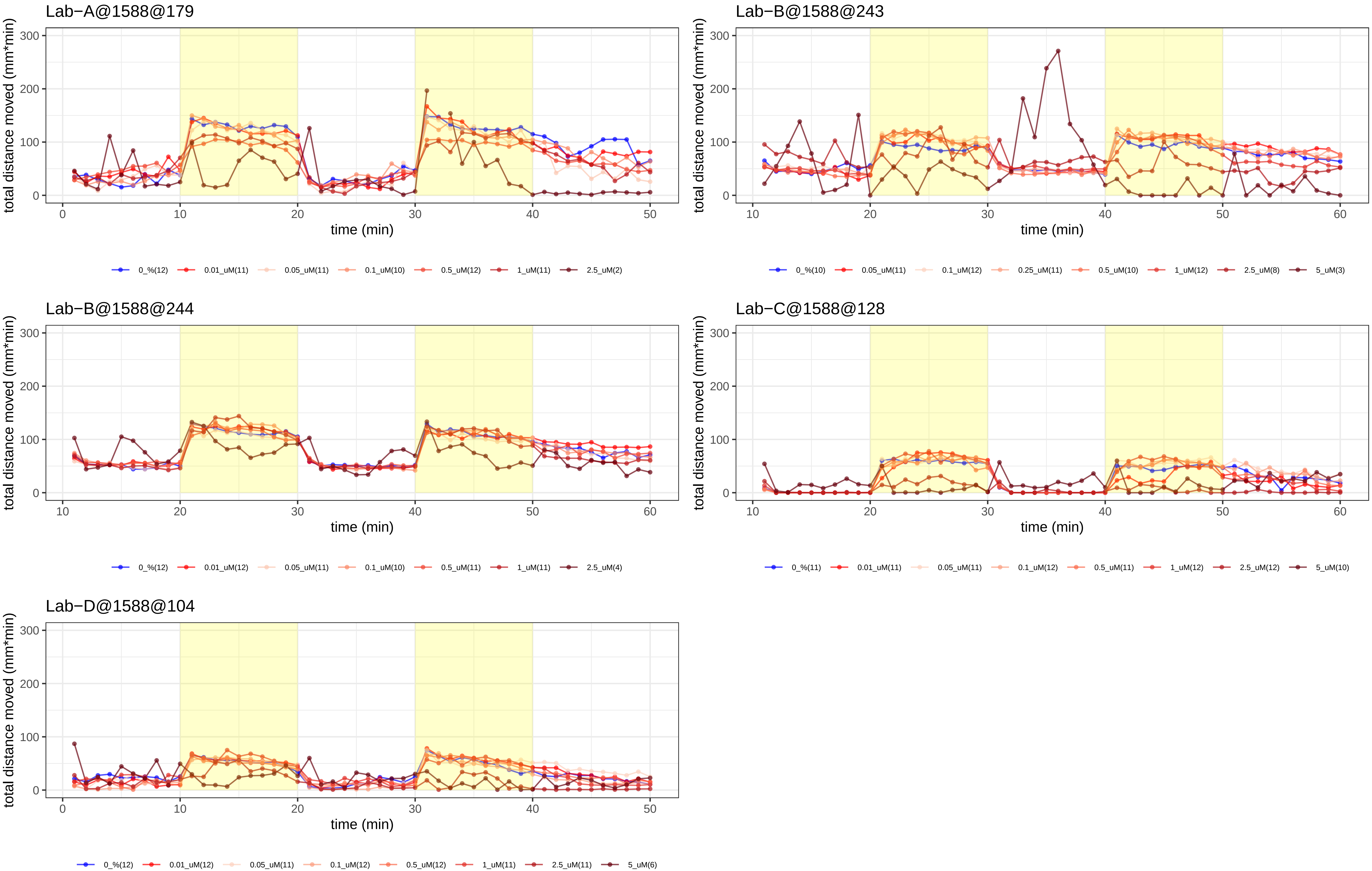

Lab−A@1588@179
Lab−B@1588@243
Lab−B@1588@244
Lab−C@1588@128
Lab−D@1588@104
total distance moved (mm*min)
time (min)
0_%(12) 0.01_uM(11) 0.05_uM(11) 0.1_uM(10) 0.5_uM(12) 1_uM(11) 2.5_uM(2)
0_%(10) 0.05_uM(11) 0.1_uM(12) 0.25_uM(11) 0.5_uM(10) 1_uM(12) 2.5_uM(8) 5_uM(3)
0_%(12) 0.01_uM(12) 0.05_uM(11) 0.1_uM(10) 0.5_uM(11) 1_uM(11) 2.5_uM(4)
0_%(11) 0.01_uM(11) 0.05_uM(11) 0.1_uM(12) 0.5_uM(11) 1_uM(12) 2.5_uM(12) 5_uM(10)
0_%(12) 0.01_uM(12) 0.05_uM(11) 0.1_uM(12) 0.5_uM(12) 1_uM(11) 2.5_uM(11) 5_uM(6)

Deltamethrin

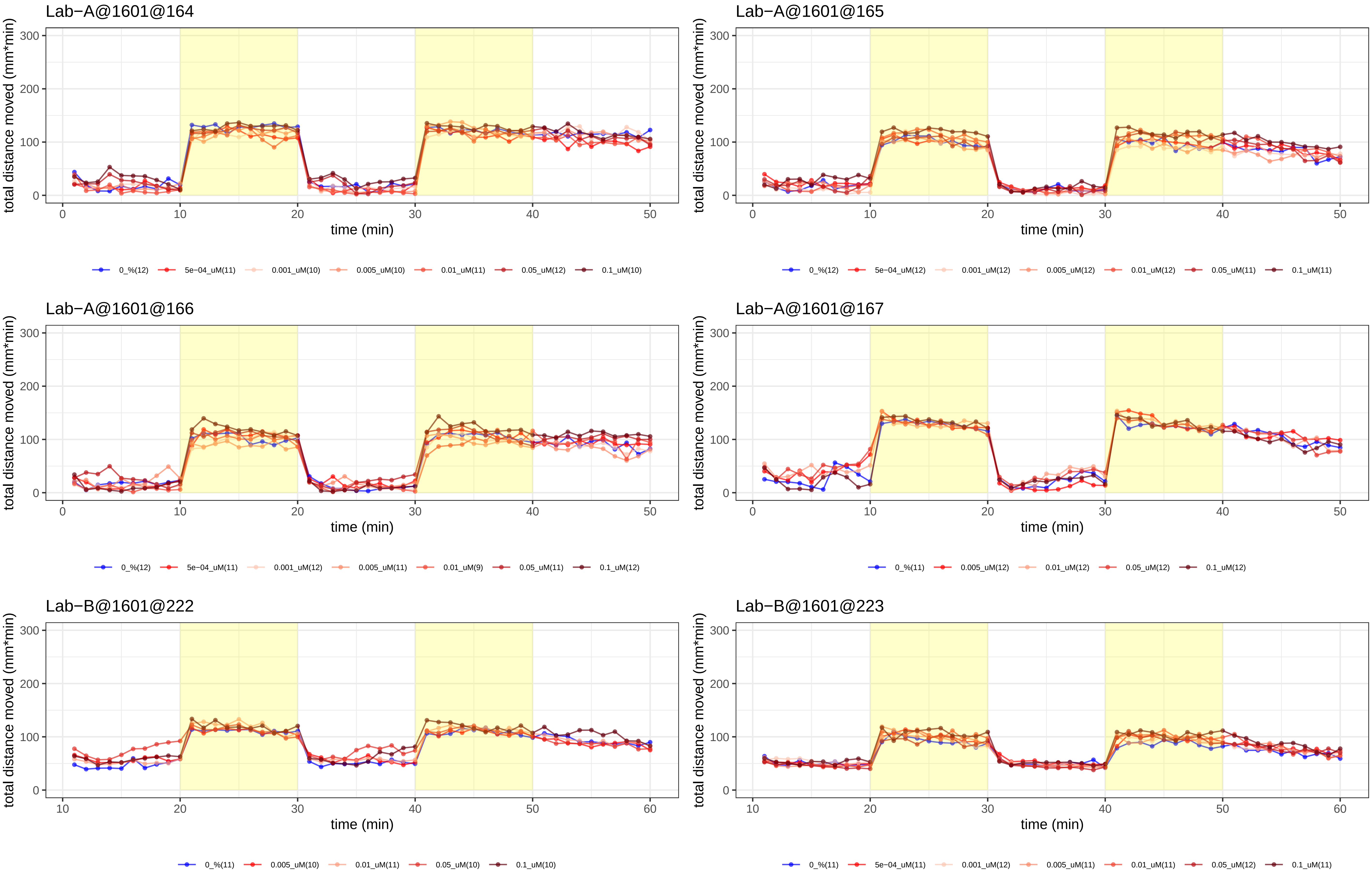

Deltamethrin

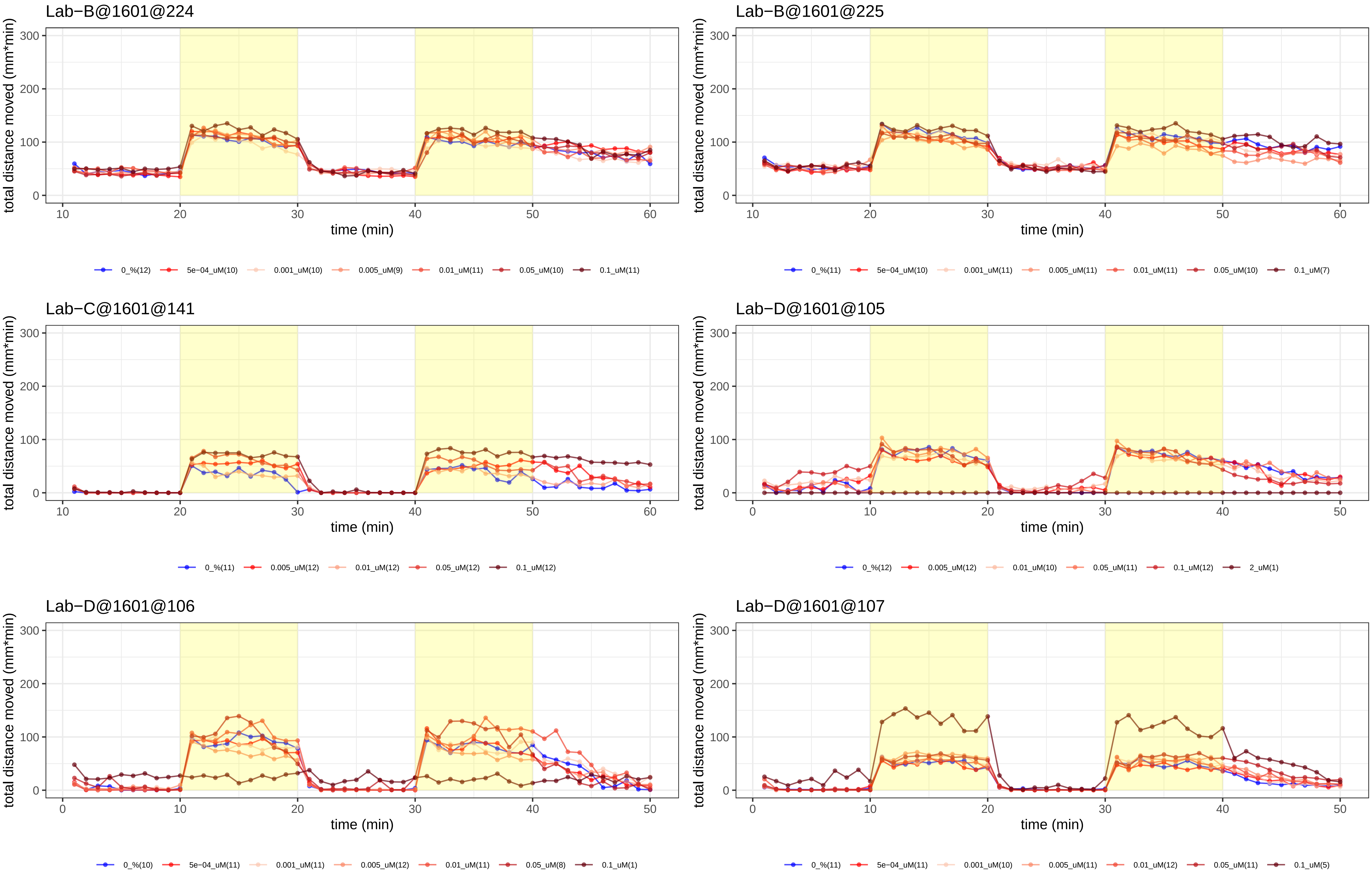

Deltamethrin

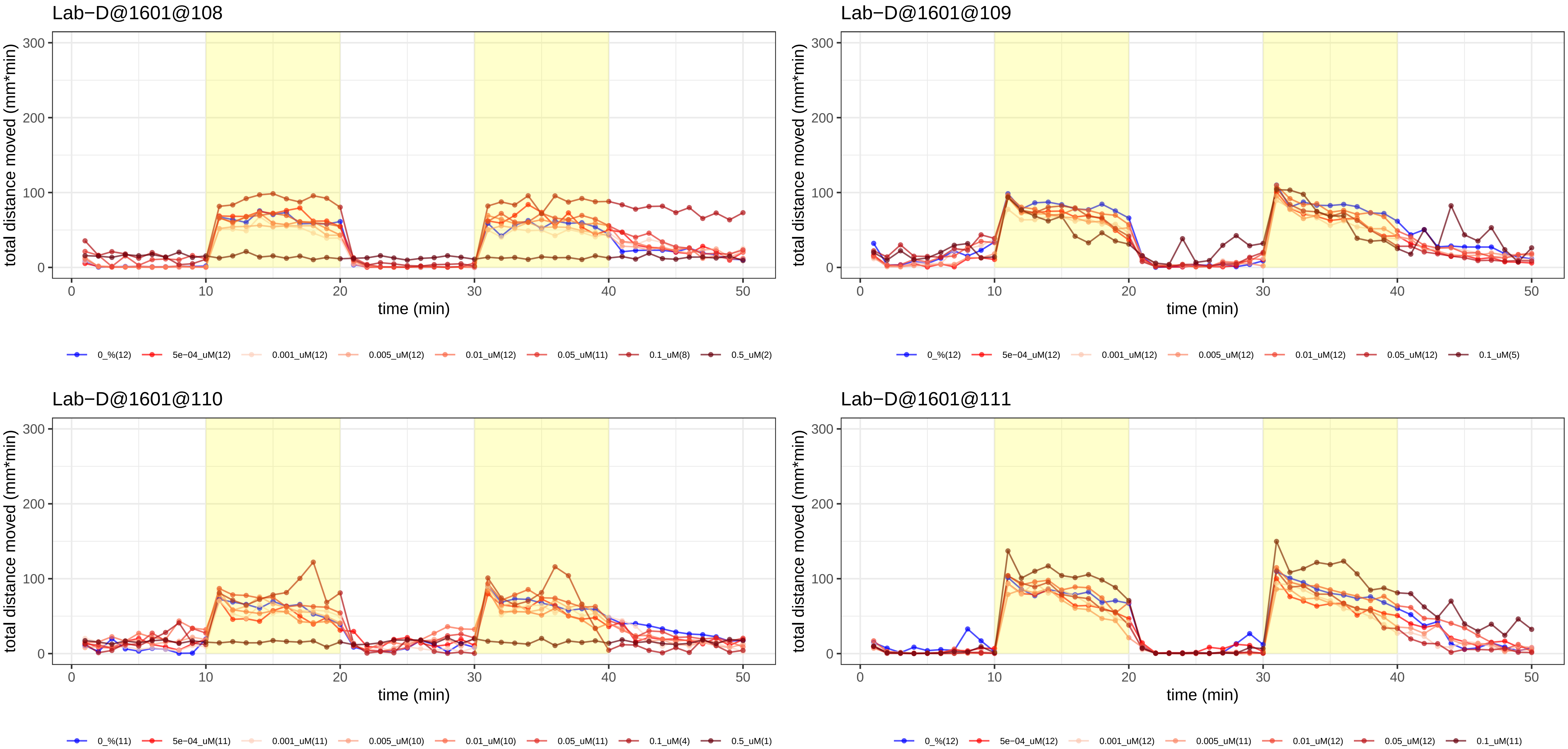

Nicotine

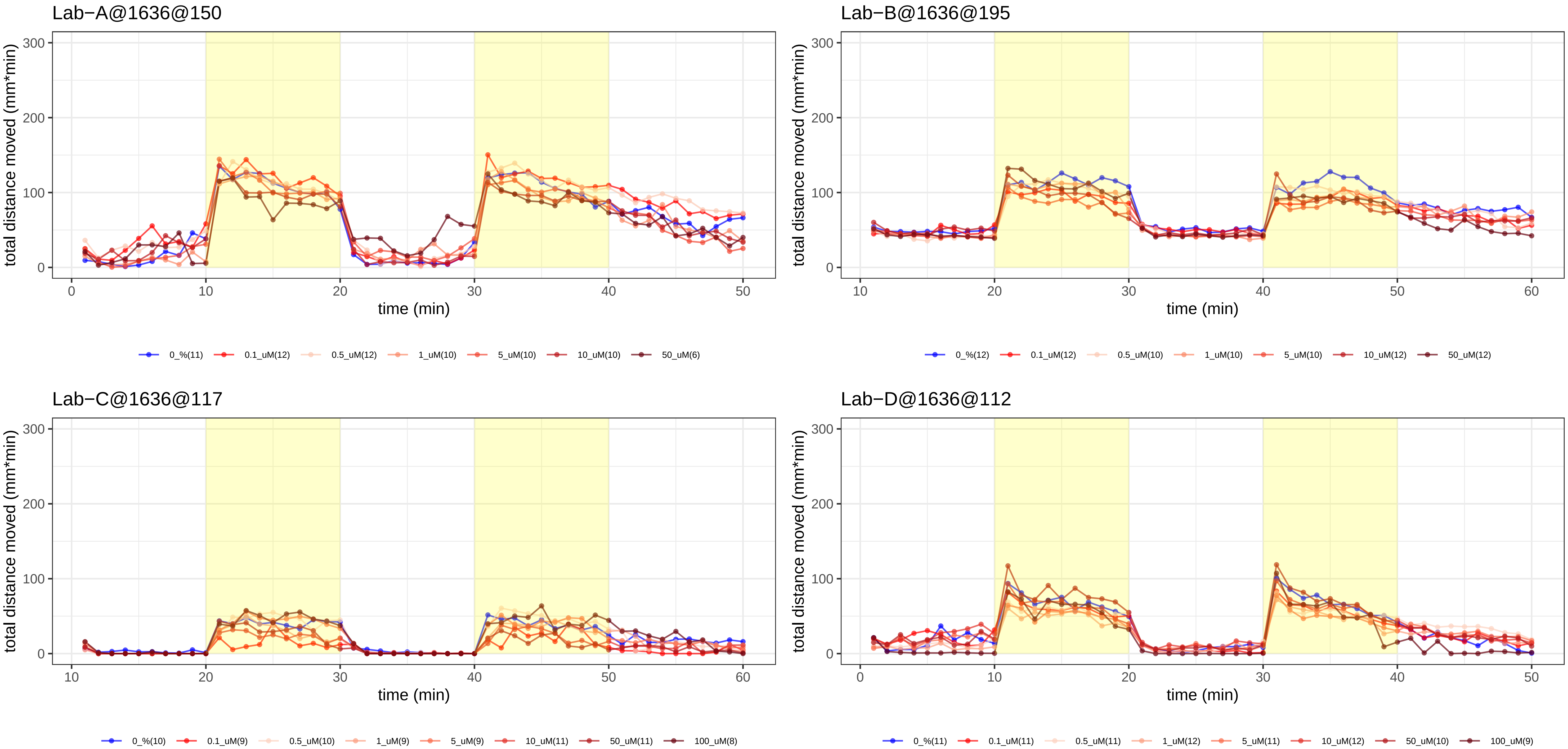

Lab−A@1636@150
Lab−B@1636@195
Lab−C@1636@117
Lab−D@1636@112
total distance moved (mm*min)
time (min)
0_%(11)
0.1_uM(12)
0.5_uM(12)
1_uM(10)
5_uM(10)
10_uM(10)
50_uM(6)
0_%(12)
0.1_uM(12)
0.5_uM(10)
1_uM(10)
5_uM(10)
10_uM(12)
50_uM(12)
0_%(10)
0.1_uM(9)
0.5_uM(10)
1_uM(9)
5_uM(9)
10_uM(11)
50_uM(11)
100_uM(8)
0_%(11)
0.1_uM(11)
0.5_uM(11)
1_uM(12)
5_uM(11)
10_uM(12)
50_uM(10)
100_uM(9)

Parathion

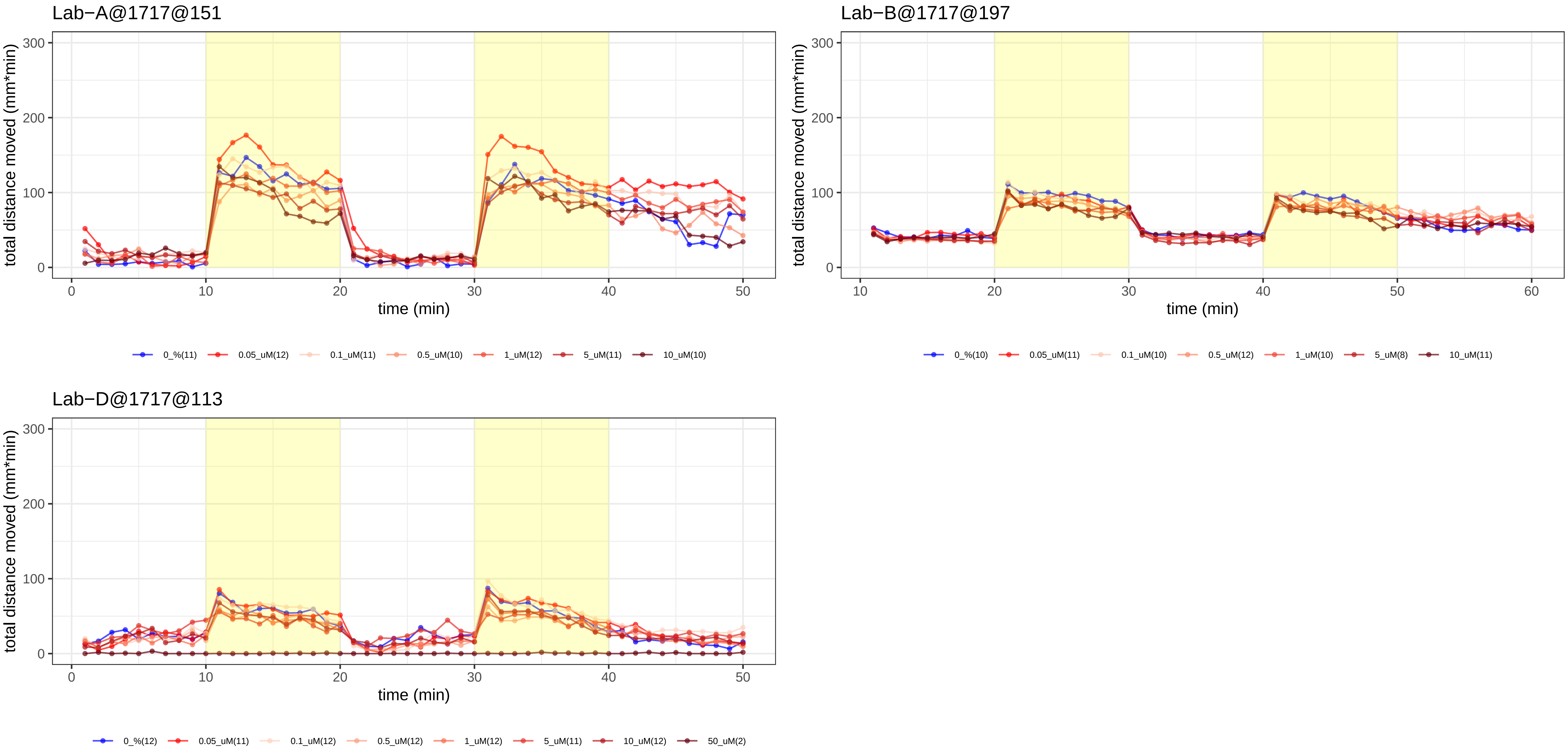
Lab−A@1717@151
total distance moved (mm*min)
0
100
200
300
time (min)
0
10
20
30
40
50
0_%(11)
0.05_uM(12)
0.1_uM(11)
0.5_uM(10)
1_uM(12)
5_uM(11)
10_uM(10)
Lab−B@1717@197
total distance moved (mm*min)
0
100
200
300
time (min)
10
20
30
40
50
60
0_%(10)
0.05_uM(11)
0.1_uM(10)
0.5_uM(12)
1_uM(10)
5_uM(8)
10_uM(11)
Lab−D@1717@113
total distance moved (mm*min)
0
100
200
300
time (min)
0
10
20
30
40
50
0_%(12)
0.05_uM(11)
0.1_uM(12)
0.5_uM(12)
1_uM(12)
5_uM(11)
10_uM(12)
50_uM(2)

Benomyl

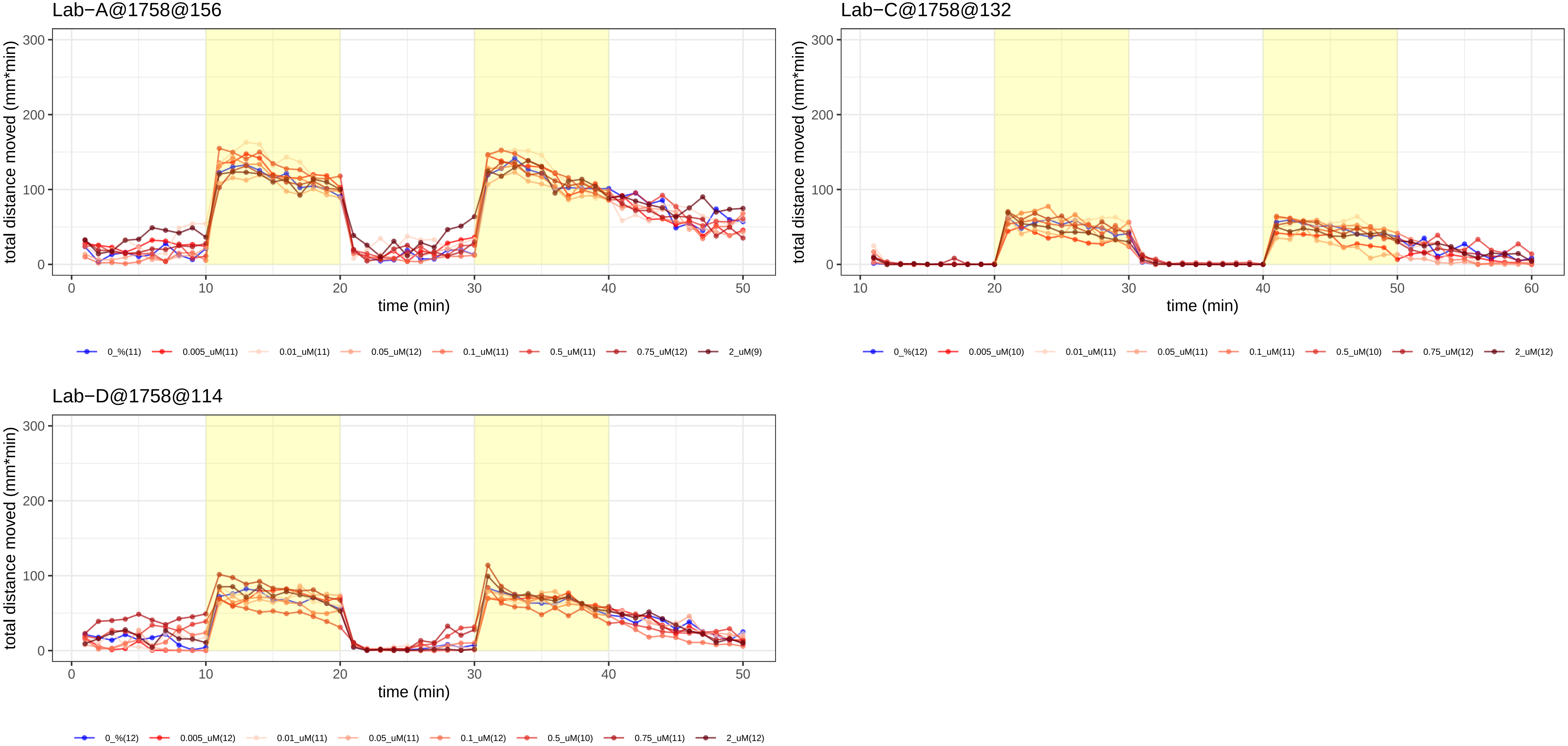

Lab−A@1758@156
Lab−C@1758@132
Lab−D@1758@114
total distance moved (mm*min)
time (min)
0_%(11) 0.005_uM(11) 0.01_uM(11) 0.05_uM(12) 0.1_uM(11) 0.5_uM(11) 0.75_uM(12) 2_uM(9)
0_%(12) 0.005_uM(10) 0.01_uM(11) 0.05_uM(11) 0.1_uM(11) 0.5_uM(10) 0.75_uM(12) 2_uM(12)
0_%(12) 0.005_uM(12) 0.01_uM(11) 0.05_uM(11) 0.1_uM(12) 0.5_uM(10) 0.75_uM(11) 2_uM(12)

Kepone

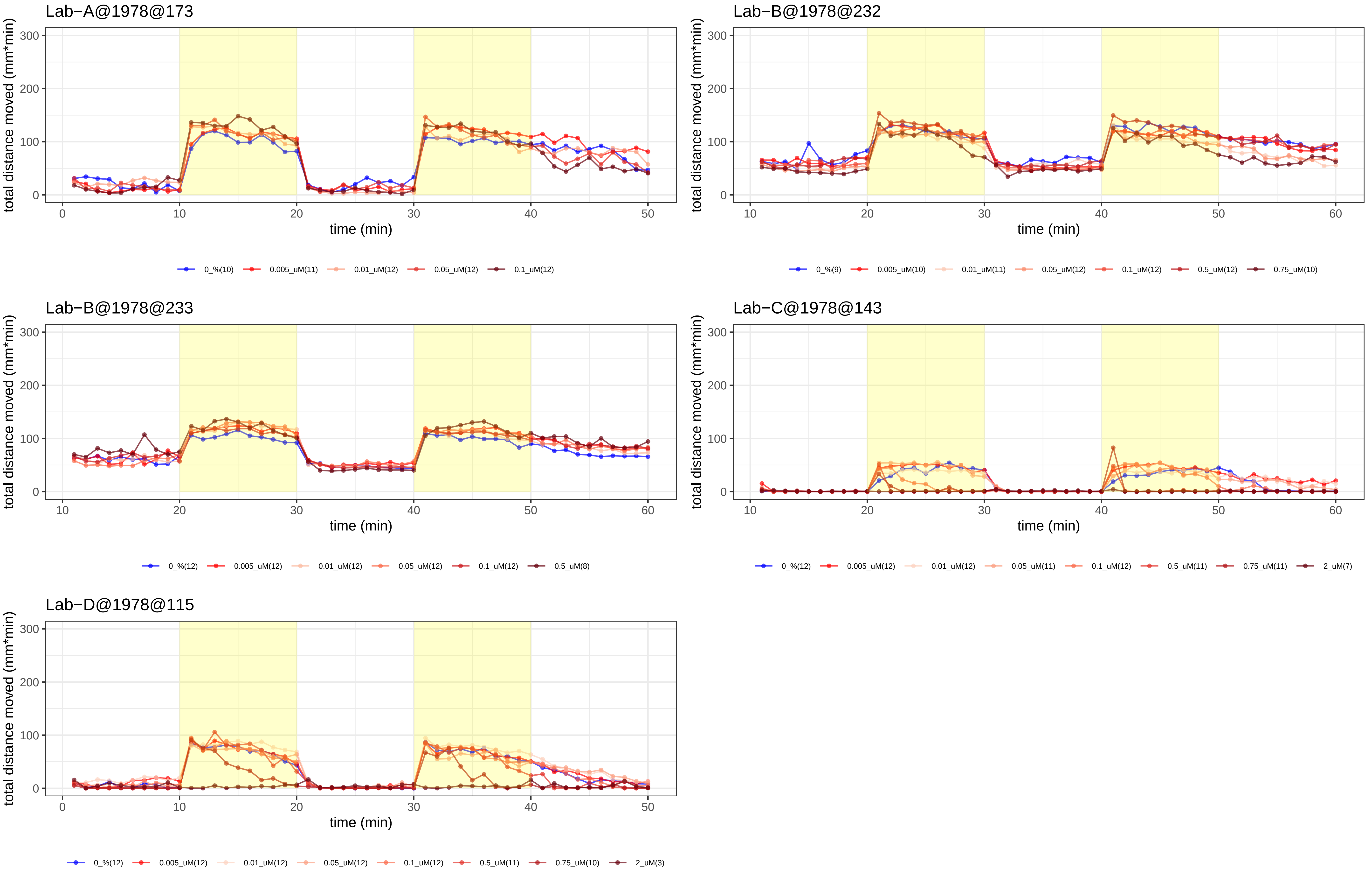

Lab−A@1978@173
Lab−B@1978@232
Lab−B@1978@233
Lab−C@1978@143
Lab−D@1978@115
total distance moved (mm*min)
time (min)
0_%(10) 0.005_uM(11) 0.01_uM(12) 0.05_uM(12) 0.1_uM(12)
0_%(9) 0.005_uM(10) 0.01_uM(11) 0.05_uM(12) 0.1_uM(12) 0.5_uM(12) 0.75_uM(10)
0_%(12) 0.005_uM(12) 0.01_uM(12) 0.05_uM(12) 0.1_uM(12) 0.5_uM(8)
0_%(12) 0.005_uM(12) 0.01_uM(12) 0.05_uM(11) 0.1_uM(12) 0.5_uM(11) 0.75_uM(11) 2_uM(7)
0_%(12) 0.005_uM(12) 0.01_uM(12) 0.05_uM(12) 0.1_uM(12) 0.5_uM(11) 0.75_uM(10) 2_uM(3)

Methyl parathion

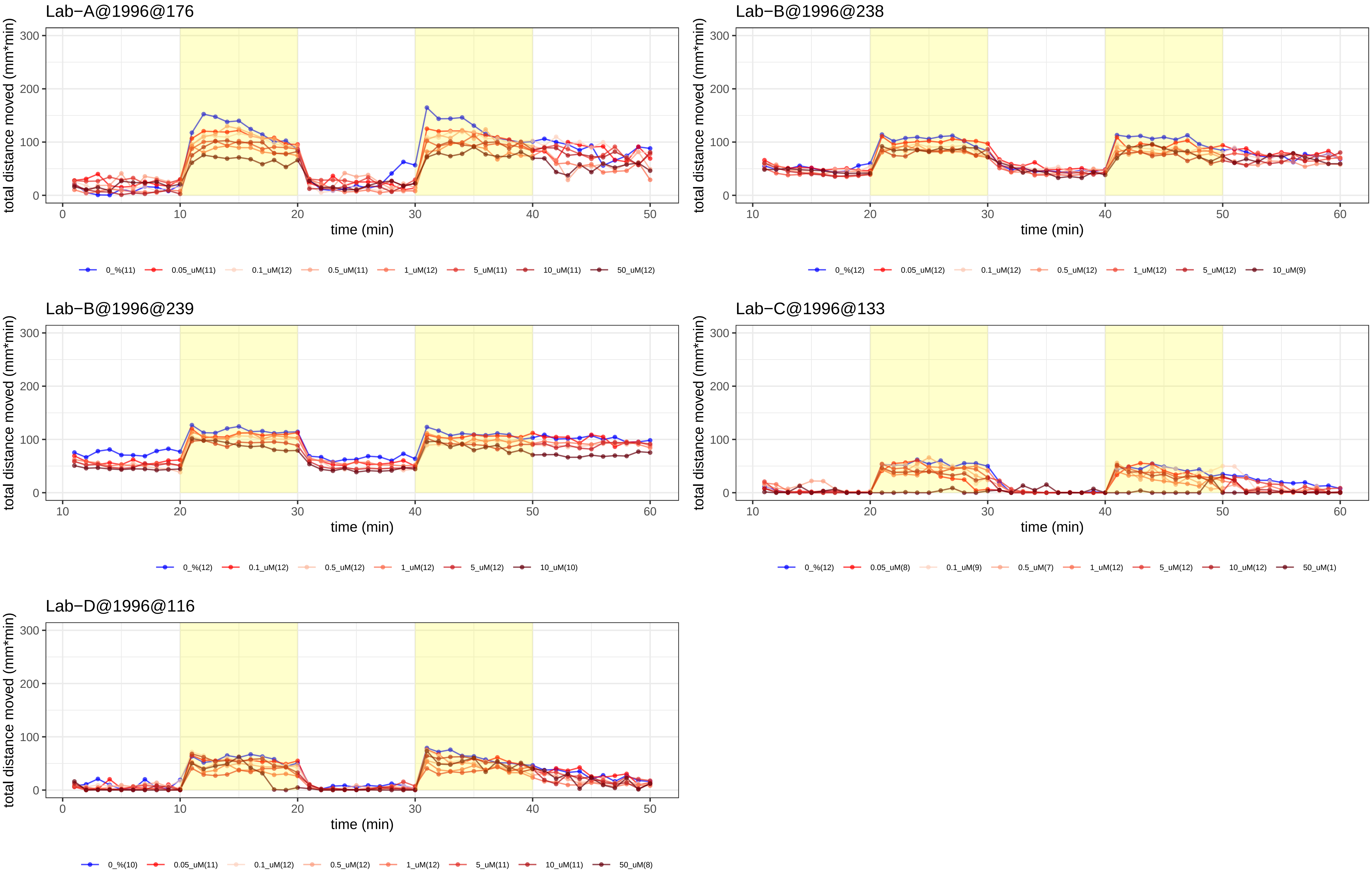

Glycerol

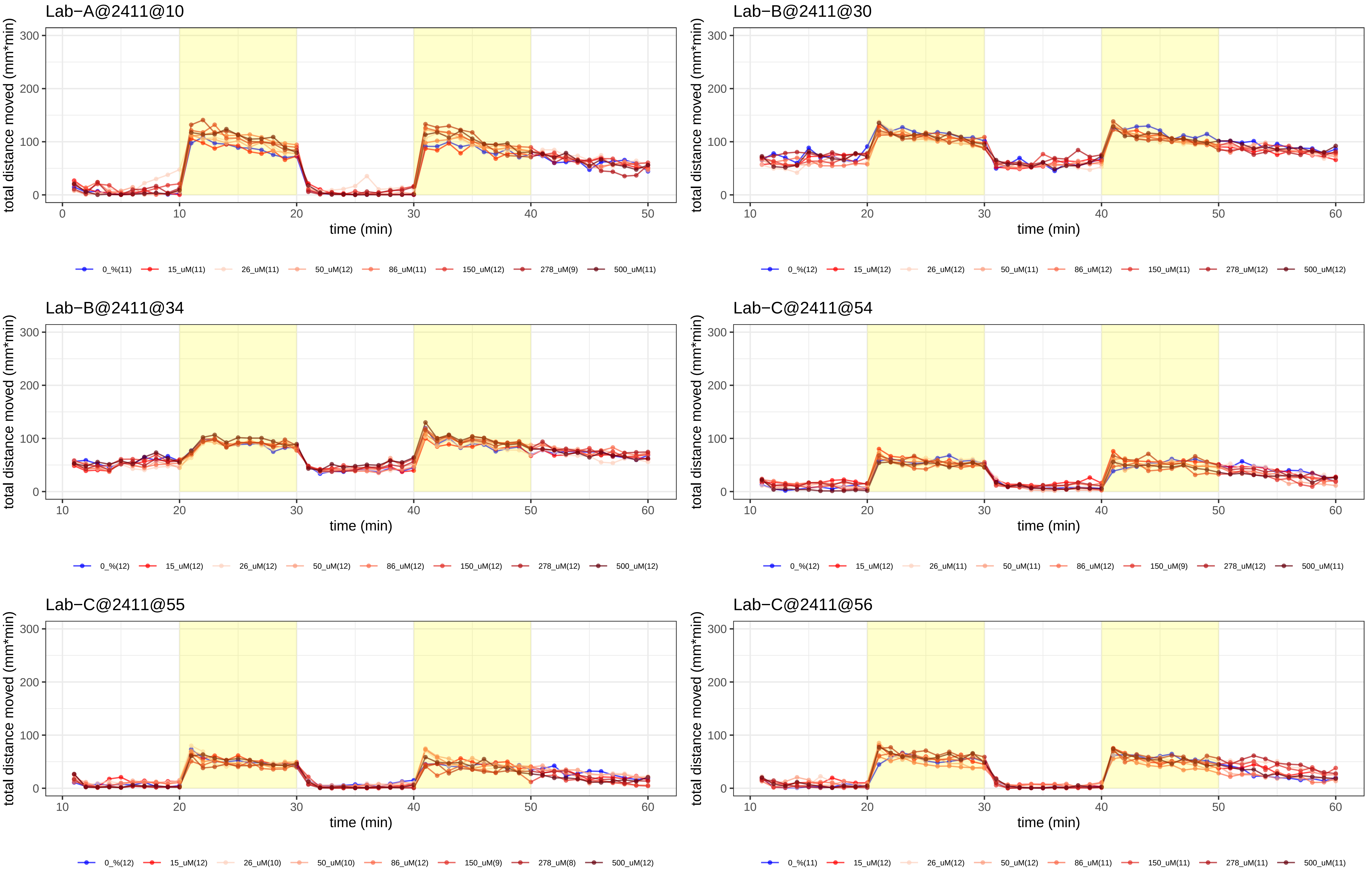
Lab−A@2411@10
total distance moved (mm*min)
time (min)
0_%(11) 15_uM(11) 26_uM(11) 50_uM(12) 86_uM(11) 150_uM(12) 278_uM(9) 500_uM(11)
Lab−B@2411@30
total distance moved (mm*min)
time (min)
0_%(12) 15_uM(12) 26_uM(12) 50_uM(11) 86_uM(12) 150_uM(11) 278_uM(12) 500_uM(12)
Lab−B@2411@34
total distance moved (mm*min)
time (min)
0_%(12) 15_uM(12) 26_uM(12) 50_uM(12) 86_uM(12) 150_uM(12) 278_uM(12) 500_uM(12)
Lab−C@2411@54
total distance moved (mm*min)
time (min)
0_%(12) 15_uM(12) 26_uM(11) 50_uM(11) 86_uM(12) 150_uM(9) 278_uM(12) 500_uM(11)
Lab−C@2411@55
total distance moved (mm*min)
time (min)
0_%(12) 15_uM(12) 26_uM(10) 50_uM(10) 86_uM(12) 150_uM(9) 278_uM(8) 500_uM(12)
Lab−C@2411@56
total distance moved (mm*min)
time (min)
0_%(11) 15_uM(12) 26_uM(12) 50_uM(12) 86_uM(11) 150_uM(11) 278_uM(11) 500_uM(11)

Glycerol

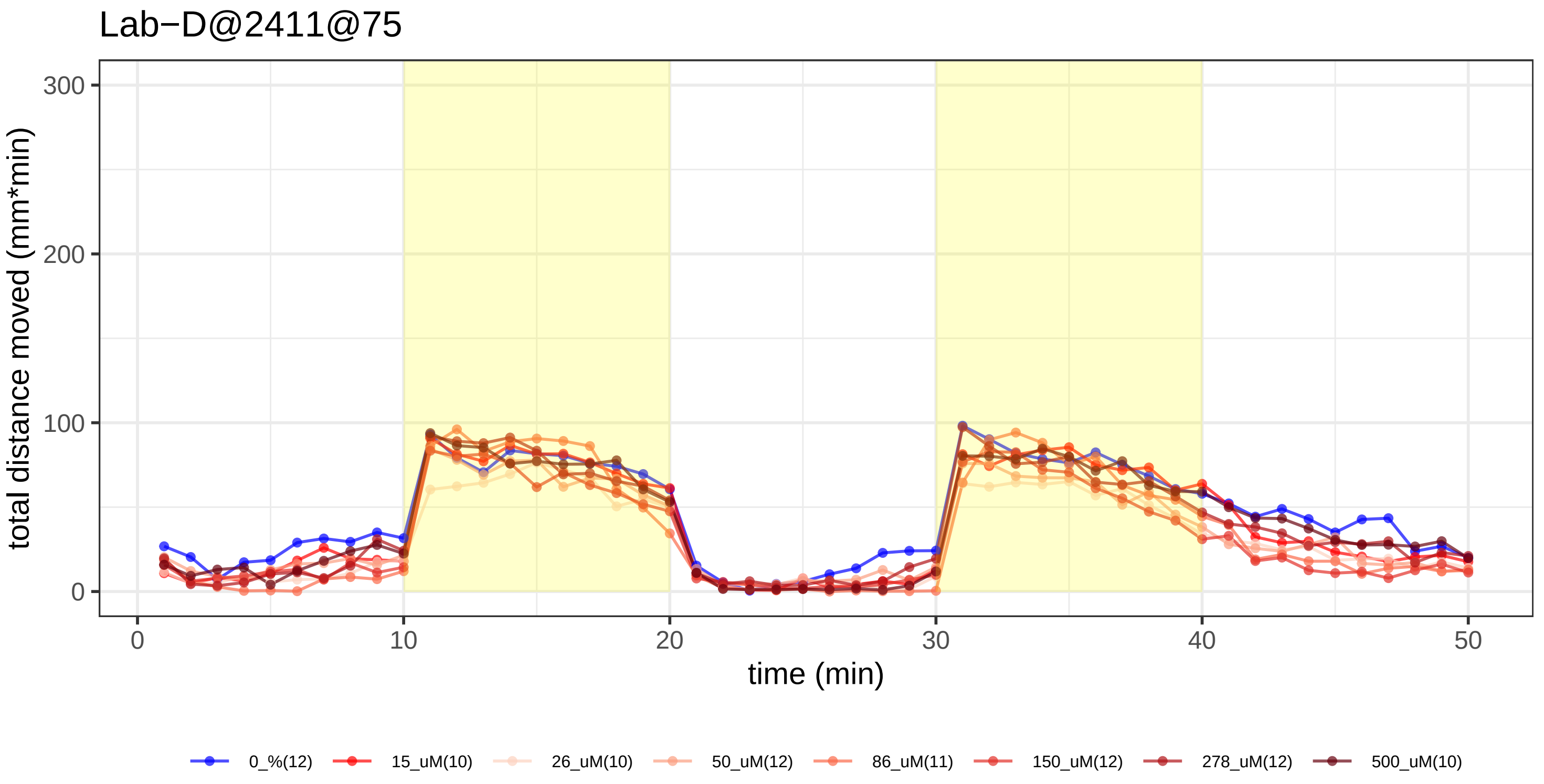

Lab−D@2411@75
300
200
100
0
total distance moved (mm*min)
0
10
20
30
40
50
time (min)
0_%(12)
15_uM(10)
26_uM(10)
50_uM(12)
86_uM(11)
150_uM(12)
278_uM(12)
500_uM(10)

Saccharin

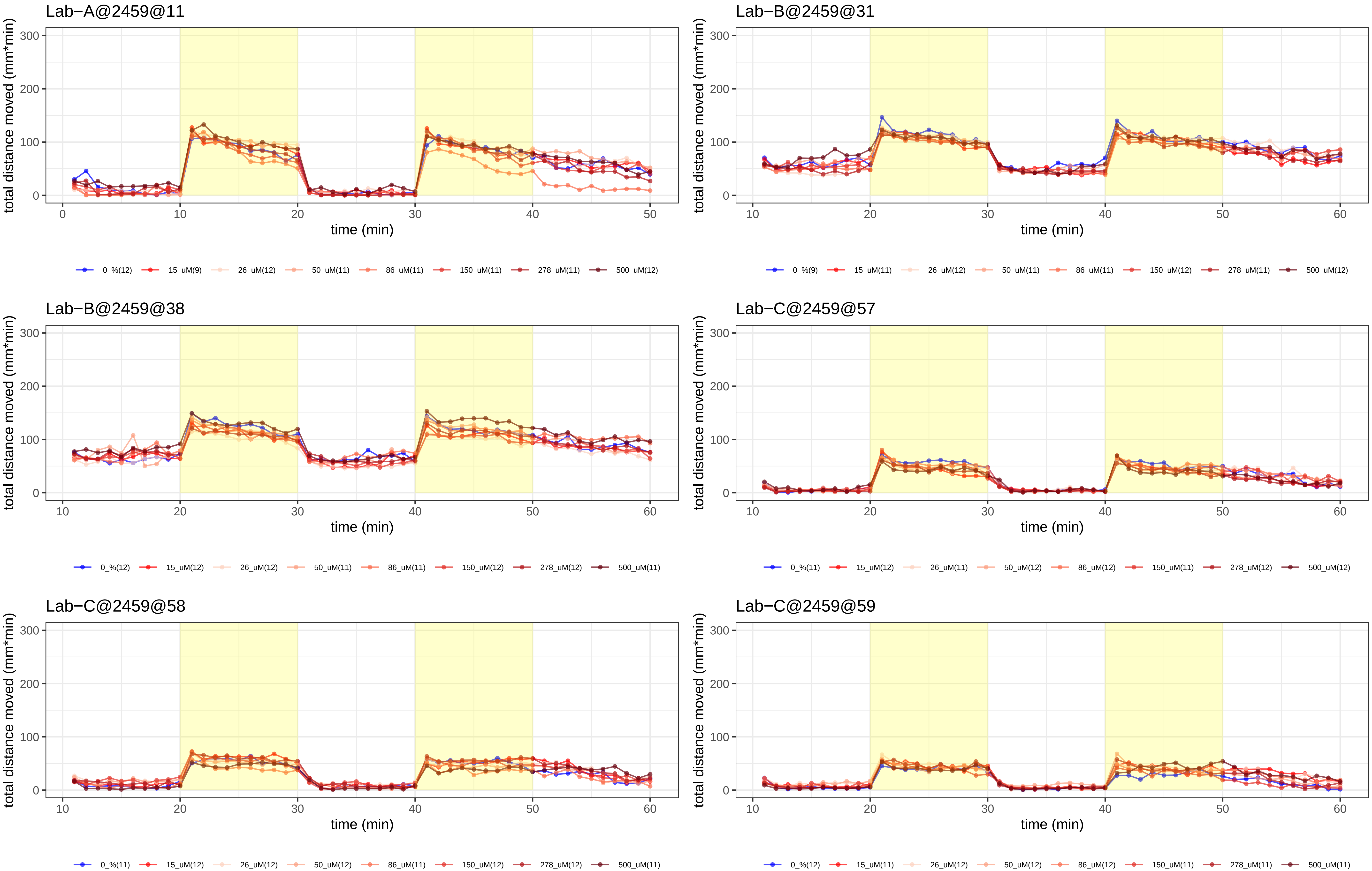
Lab−A@2459@11
Lab−B@2459@31
Lab−B@2459@38
Lab−C@2459@57
Lab−C@2459@58
Lab−C@2459@59
total distance moved (mm*min)
time (min)
0_%(12) 15_uM(9) 26_uM(12) 50_uM(11) 86_uM(11) 150_uM(11) 278_uM(11) 500_uM(12)
0_%(9) 15_uM(11) 26_uM(12) 50_uM(11) 86_uM(11) 150_uM(12) 278_uM(11) 500_uM(12)
0_%(12) 15_uM(12) 26_uM(12) 50_uM(11) 86_uM(11) 150_uM(12) 278_uM(12) 500_uM(11)
0_%(11) 15_uM(12) 26_uM(11) 50_uM(12) 86_uM(12) 150_uM(11) 278_uM(12) 500_uM(12)
0_%(11) 15_uM(12) 26_uM(12) 50_uM(12) 86_uM(11) 150_uM(12) 278_uM(12) 500_uM(11)
0_%(12) 15_uM(11) 26_uM(12) 50_uM(12) 86_uM(12) 150_uM(11) 278_uM(11) 500_uM(11)

Saccharin

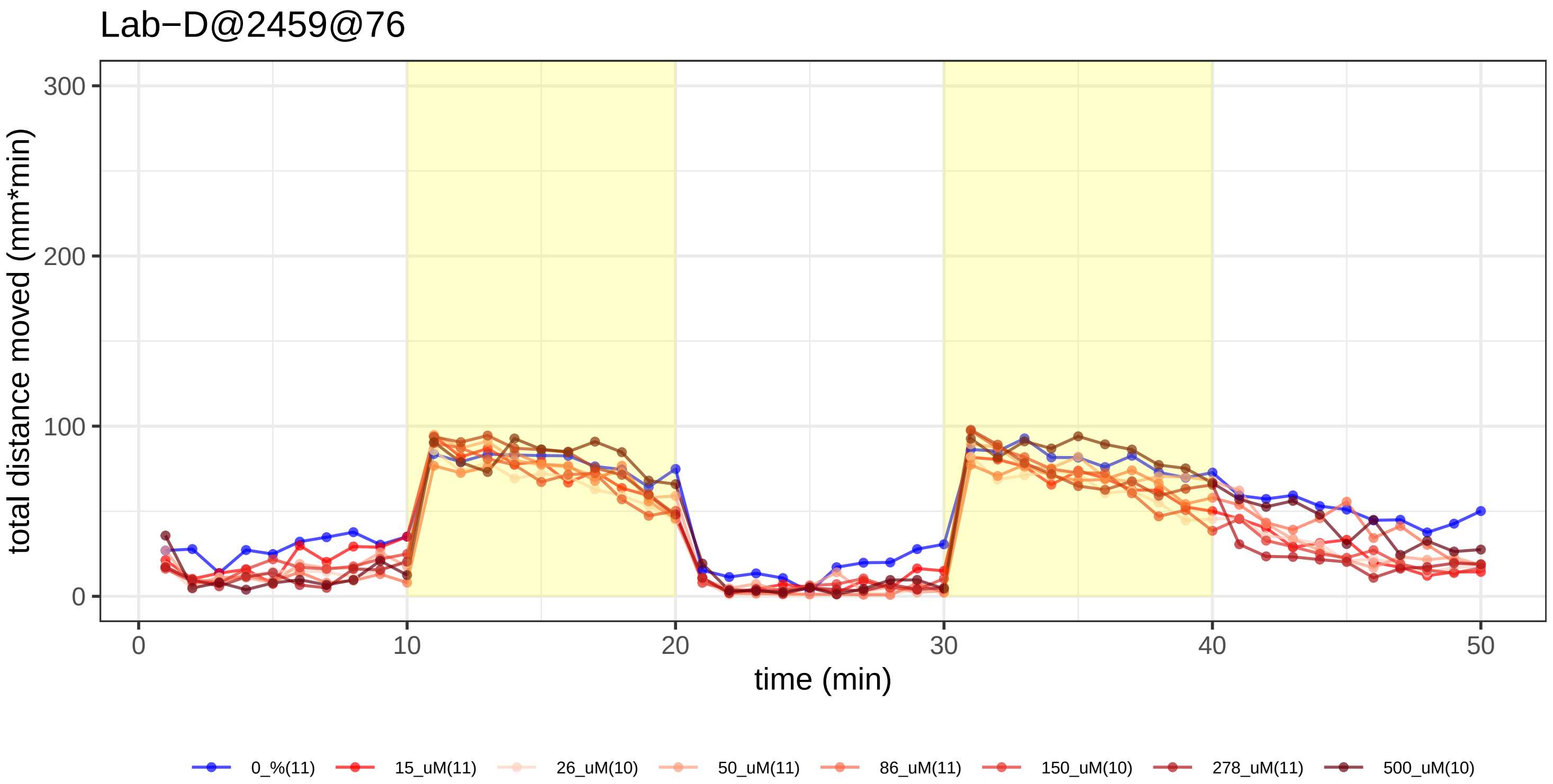

Lab−D@2459@76
total distance moved (mm*min)
300
200
100
0
0
10
20
30
40
50
time (min)
0_%(11)
15_uM(11)
26_uM(10)
50_uM(11)
86_uM(11)
150_uM(10)
278_uM(11)
500_uM(10)

Diethylene glycol

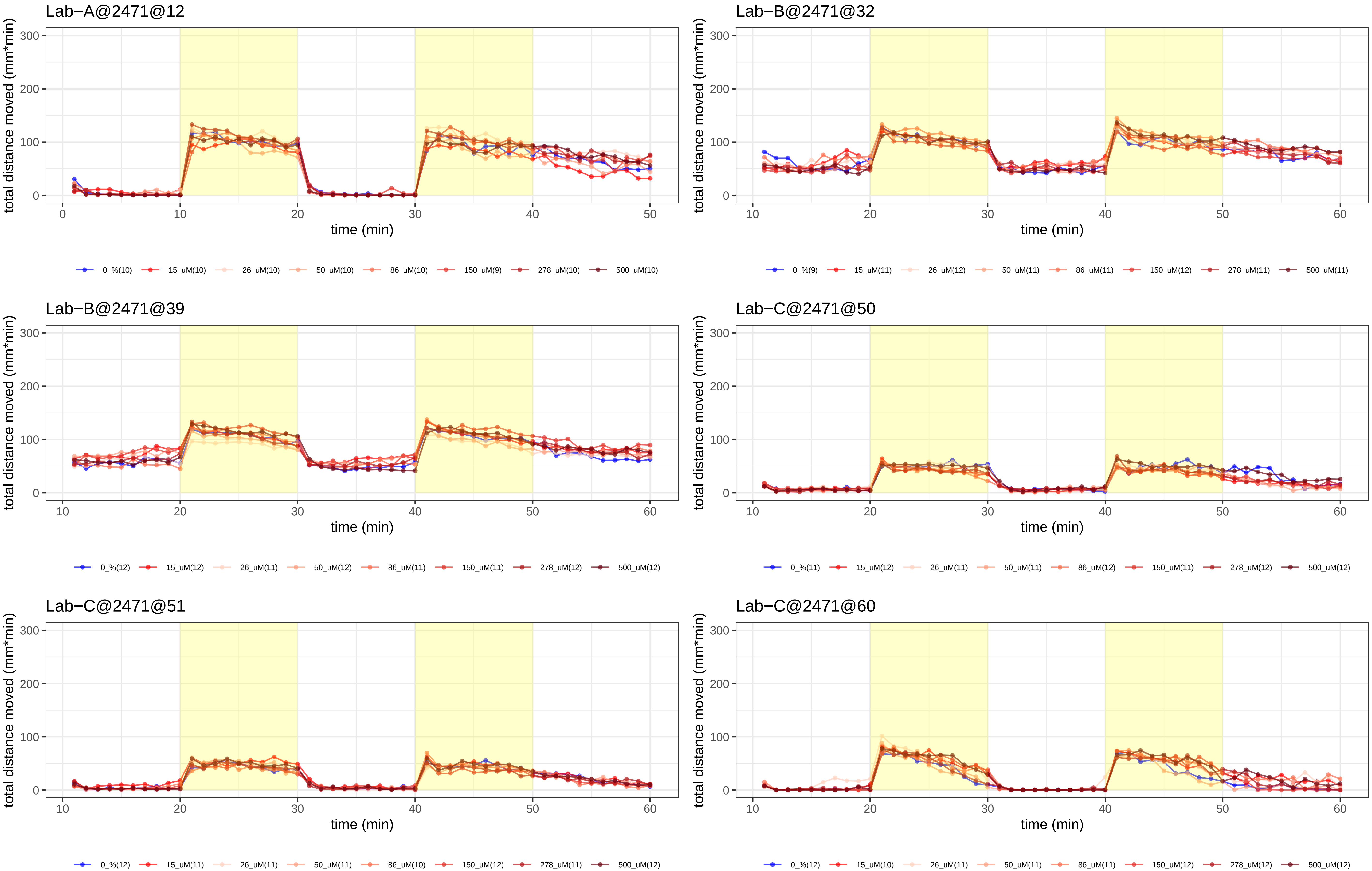

Diethylene glycol

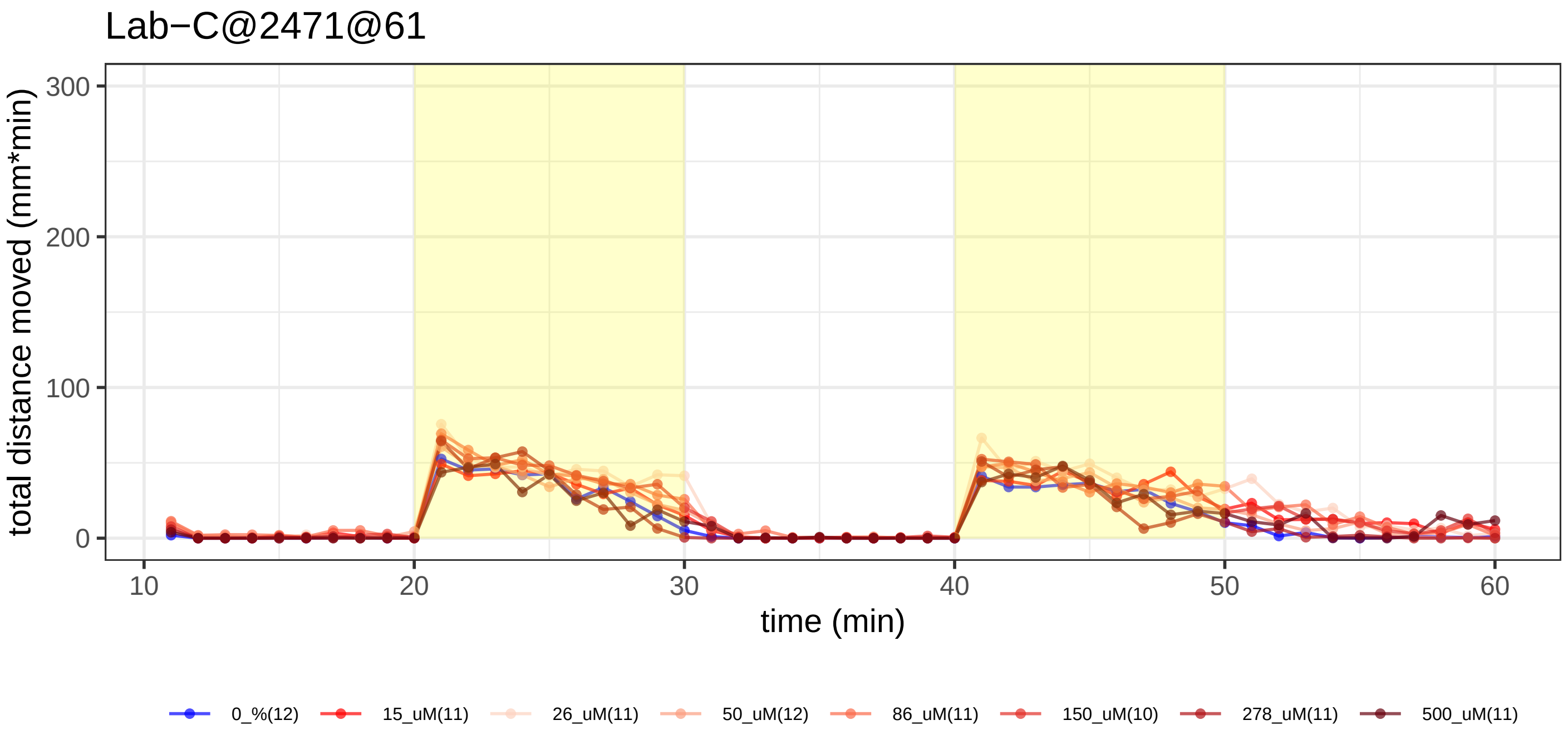

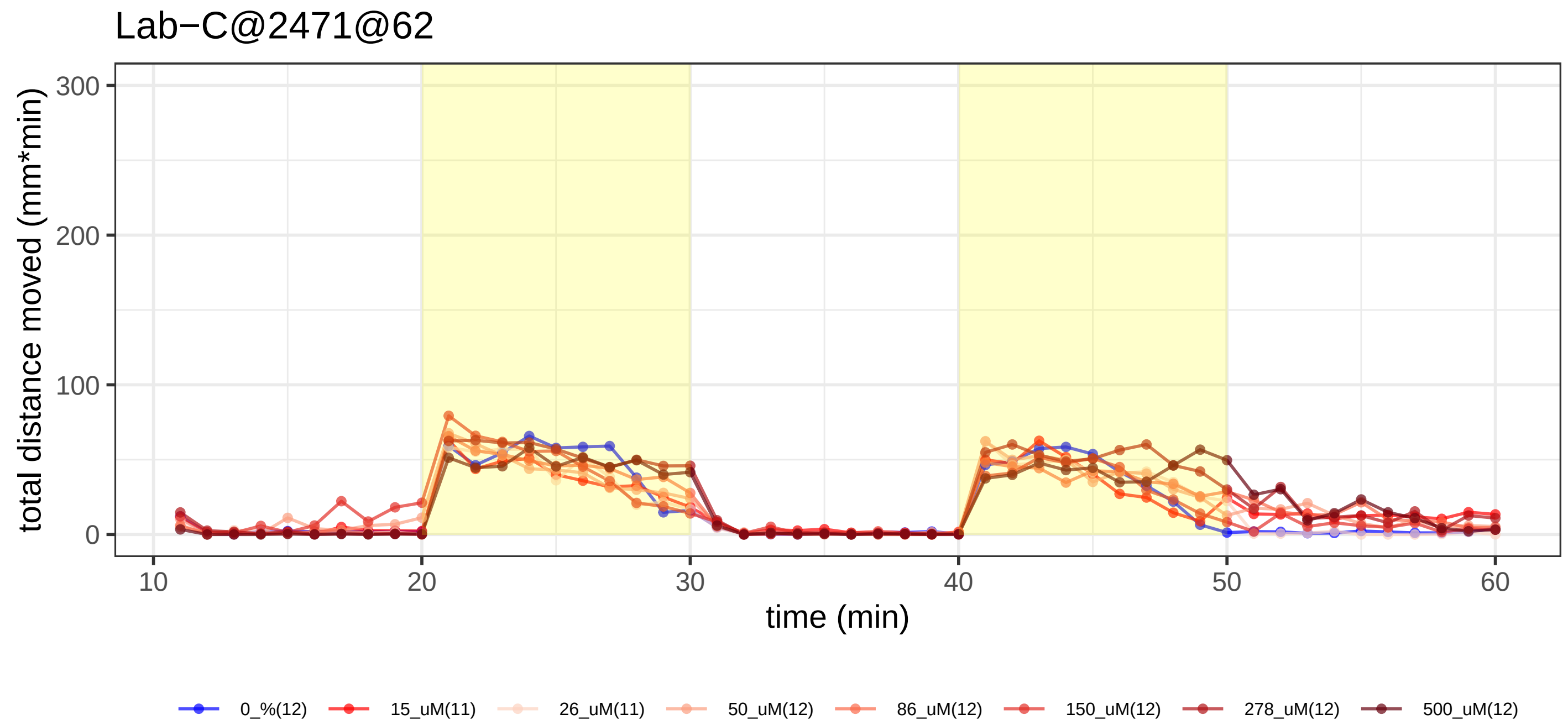

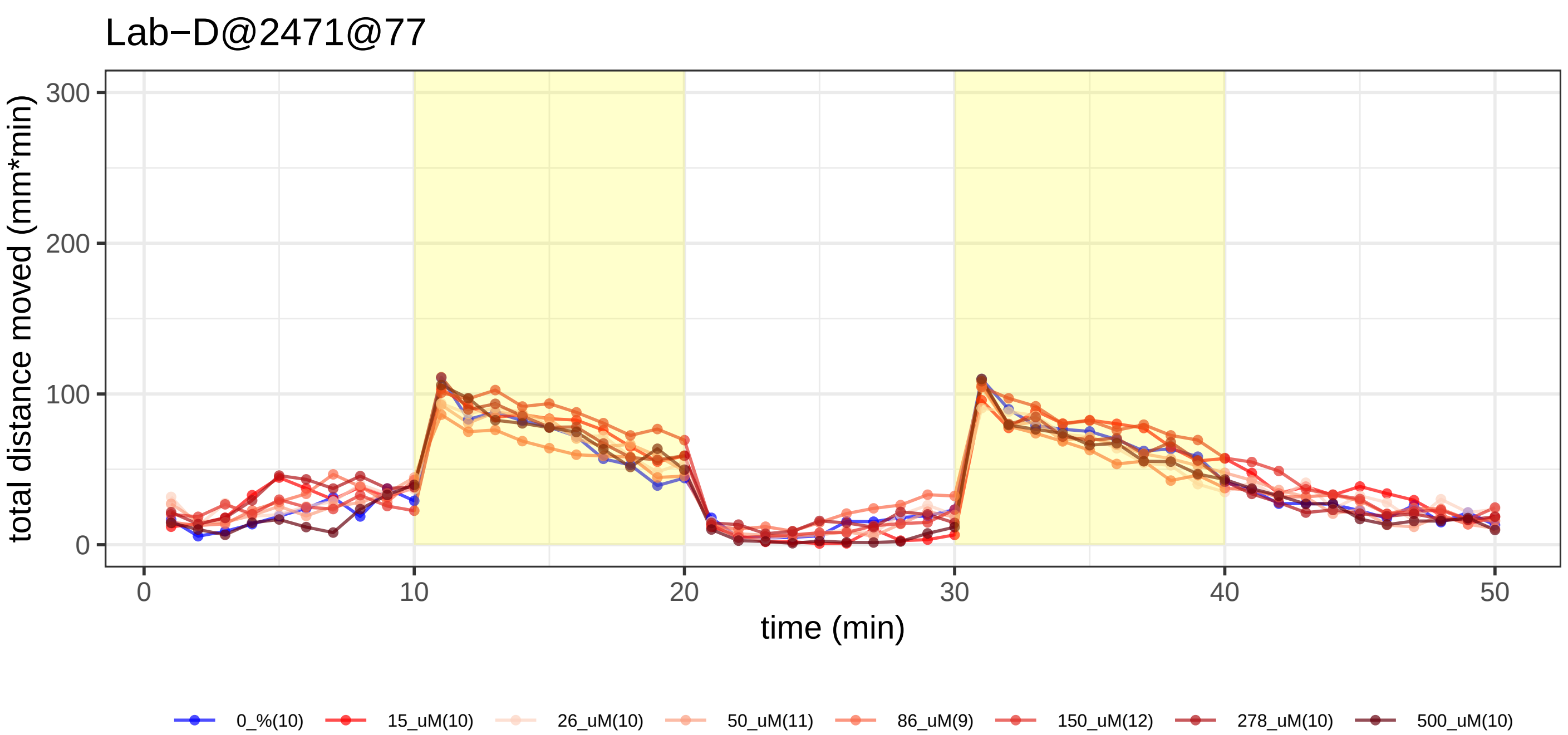

Ibuprofen

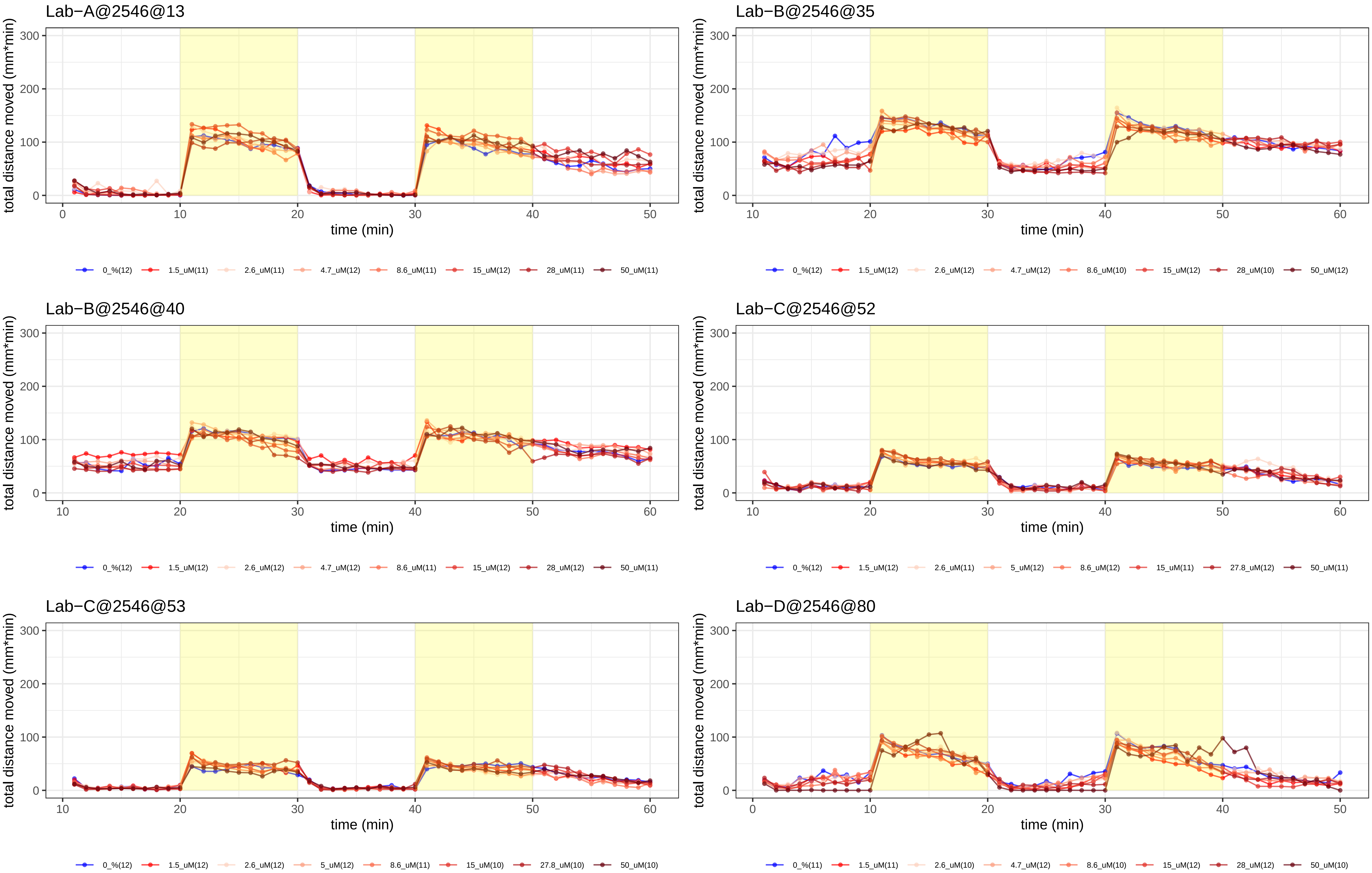

Lab−A@2546@13
Lab−B@2546@35
total distance moved (mm*min)
time (min)
0_%(12) 1.5_uM(11) 2.6_uM(11) 4.7_uM(12) 8.6_uM(11) 15_uM(12) 28_uM(11) 50_uM(11)
0_%(12) 1.5_uM(12) 2.6_uM(12) 4.7_uM(12) 8.6_uM(10) 15_uM(12) 28_uM(10) 50_uM(12)
Lab−B@2546@40
Lab−C@2546@52
0_%(12) 1.5_uM(12) 2.6_uM(12) 4.7_uM(12) 8.6_uM(11) 15_uM(12) 28_uM(12) 50_uM(11)
0_%(12) 1.5_uM(12) 2.6_uM(11) 5_uM(12) 8.6_uM(12) 15_uM(11) 27.8_uM(12) 50_uM(11)
Lab−C@2546@53
Lab−D@2546@80
0_%(12) 1.5_uM(12) 2.6_uM(12) 5_uM(12) 8.6_uM(11) 15_uM(10) 27.8_uM(10) 50_uM(10)
0_%(11) 1.5_uM(11) 2.6_uM(10) 4.7_uM(12) 8.6_uM(10) 15_uM(12) 28_uM(12) 50_uM(10)

Sodium benzoate

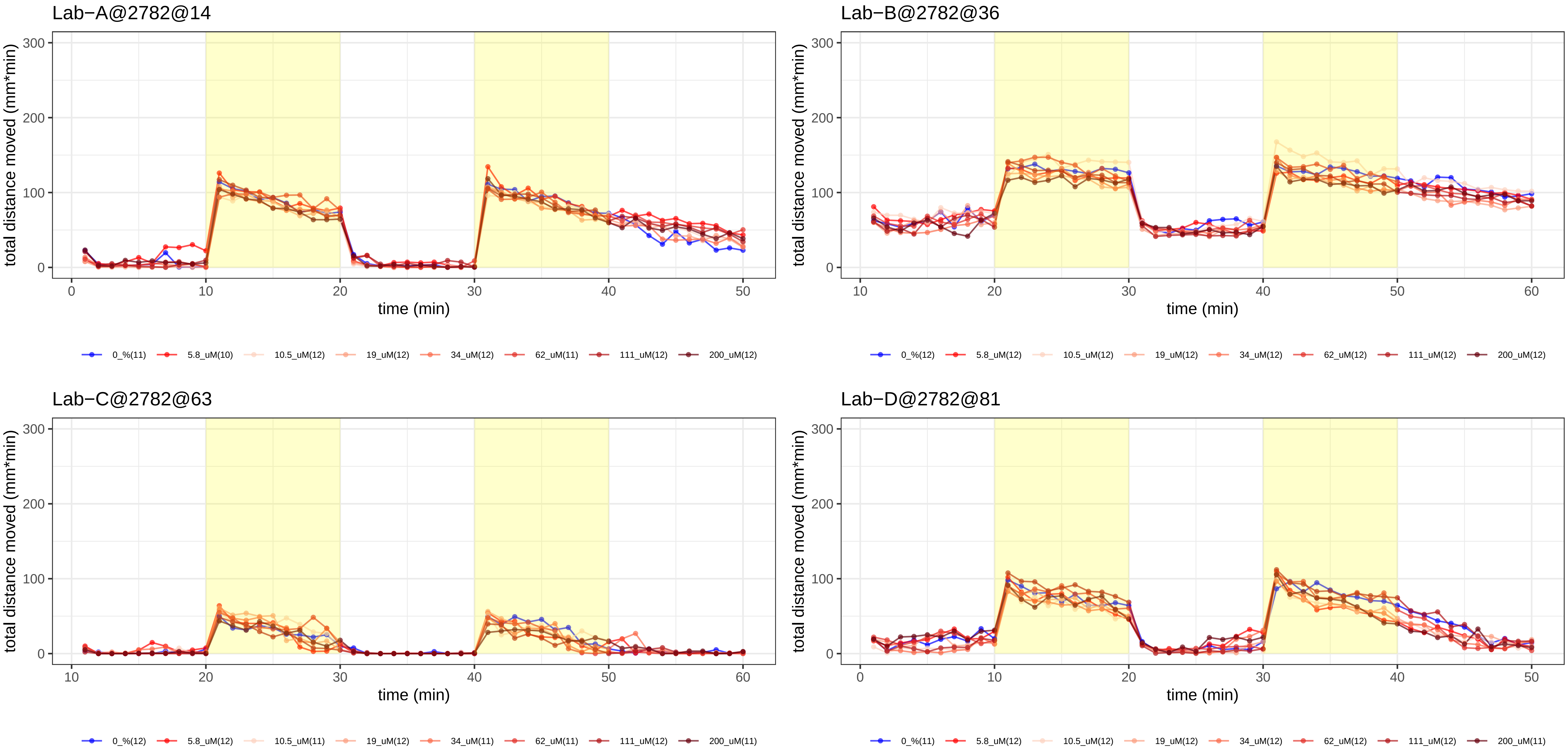

Chlorpyrifos

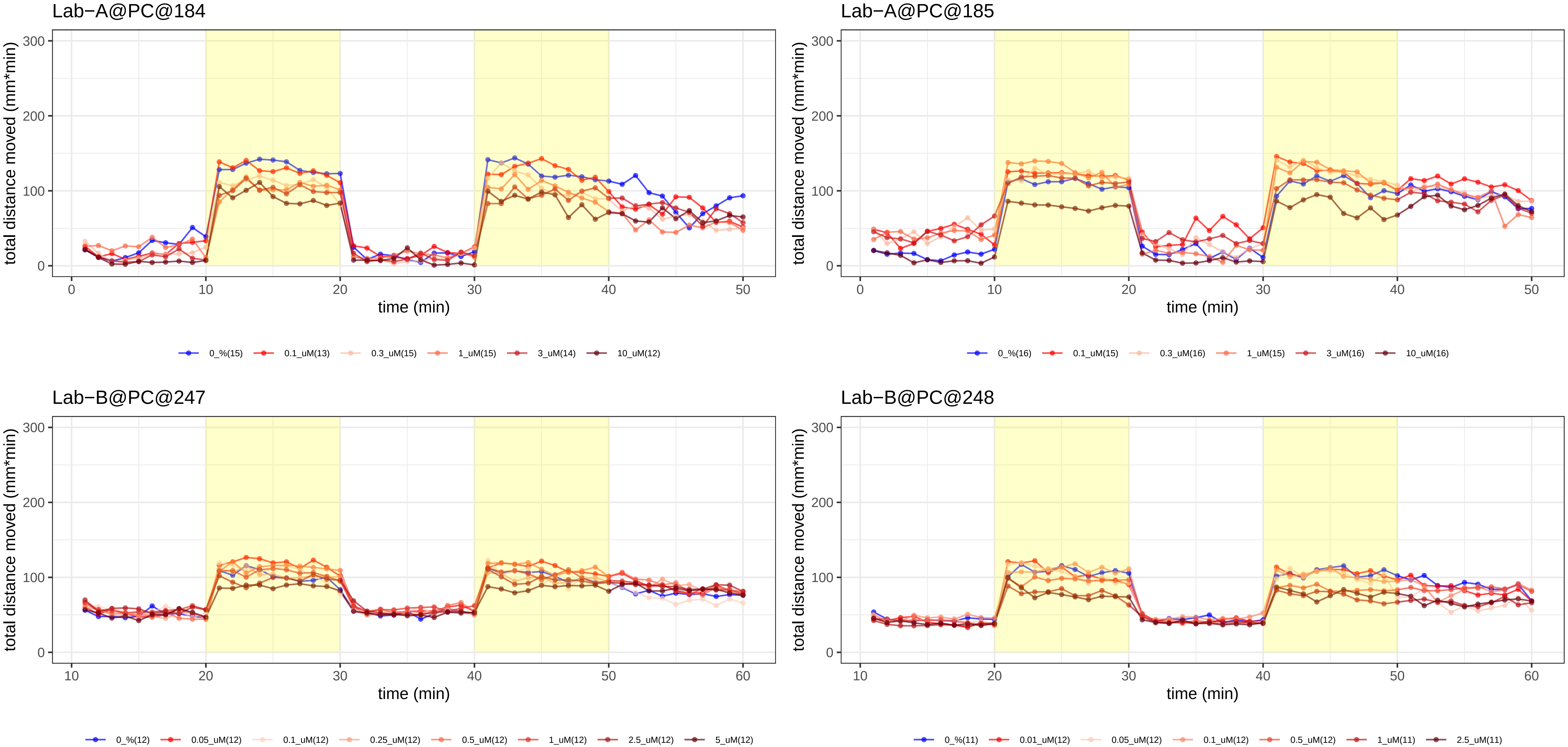

Supplement: Supplementary file 1 [file NIHMS2196518-supplement-MMC3.pdf]
