# Supplementary material for: Zebrafish behavioral assessment as a first-tier whole-organism NAM for developmental neurotoxicity: A multi-laboratory evaluation
Source: Neurotoxicology. Author manuscript; Available in PMC 2026 Aug 15. (PMC13475518; doi:10.1016/j.neuro.2026.103504)
Supplement: Supplementary file 2 [file NIHMS2196518-supplement-MMC6.docx]

# Supplemental Figures

Supplemental Figure 1: Comparison of DNT and DT potency across laboratories. Boxplots show the distribution of the log₁₀ differences between DT and DNT BMC values (log₁₀[DT BMC – DNT BMC]) for compounds that were active in both endpoints within each laboratory. Positive values indicate greater DNT potency (lower BMC), whereas negative values indicate greater DT potency. Individual points represent plate-level differences. The red dashed line at zero denotes equal potency between DT and DNT.

 Supplemental Figure 2: Heatmap representing the DNT and DT activities of 11 compounds (rows) across four laboratories (columns: Lab-A to Lab-D). One chemical was comparable across Labs A-B-C, four chemicals were comparable across Labs A-B-D, and six chemicals were comparable across Labs A-C-D. The color gradient within each cell illustrates benchmark concentration (BMC) values in µM, ranging from 0.01 µM (lighter shades) to 100 µM (darker shades). Gray cells indicate inactive effects. Blank cells indicate “no comparable data”. Compounds have been hierarchically clustered based on their DNT activities to highlight patterns and similarities. An additional column, labeled as 'top’ (duplicated on both sides for easy comparison), displays the highest concentration used in the assays for each compound. The use category of each compound and their associated logKow values are presented in separate columns. Within the DNT cells, the numbers denote the count of active endpoints identified.

Supplemental Figure 3: This plot presents the benchmark concentrations (BMC) for the effects of 11 compounds in zebrafish, illustrating DNT (red markers) and DT (black markers) effect, as depicted in the heatmap in Supplemental Figure 2. The order of the compounds follows the sequence used in Supplemental Figure 2. One chemical was comparable across Labs A-B-C, four chemicals were comparable across Labs A-B-D, and six chemicals were comparable across Labs A-C-D. Each red/black line can feature up to four dots, representing the DNT/DT activities evaluated in four different laboratories. An "X" on top of a dot indicates that the compound was inactive in that specific lab; when inactive, the highest tested concentration is reported as the BMC for plotting purpose.

# Supplemental Data

The supplemental data provide plate-level, endpoint-level, and potency-level datasets supporting all analyses presented in the manuscript. Only data from plates that passed all quality-control (QC) steps are included. The rds files can be opened directly in R (e.g., using readRDS()). The chemical code to chemical name mapping table is provided below. The data can be downloaded from the Chemical Effects in Biological Systems (CEBS) page ([https://cebs.niehs.nih.gov/cebs/](https://cebs-ext.niehs.nih.gov/cebs/paper/16239/private/MTlkYTM2ZDgyMmZjZDBhNWYwN2IyNzZiNTgwOWExYzcK)).

- **Chemical code to Chemical Name Mapping**

| **Chemical Code** | **Chemical Name** |
| --- | --- |
| 1000 | Tris(1,3-dichloro-2-propyl) phosphate (TDCIPP) |
| 1005 | Acetamiprid |
| 1029 | Cypermethrin |
| 1050 | Triphenyl phosphates isopropylated (IPP) |
| 1056 | Tris(2-chloroisopropyl)phosphate |
| 1085 | Diazinon |
| 1087 | Trichlorfon |
| 1132 | tert-Butylphenyl diphenyl phosphate (BPDP) |
| 1143 | Thiacloprid |
| 1175 | 3,3',5,5'-Tetrabromobisphenol A (TBBPA) |
| 1191 | Tri-o-cresyl phosphate |
| 1213 | Chloramben |
| 1233 | Dimethoate |
| 1239 | Heptachlor |
| 1246 | 2-Ethylhexyl diphenyl phosphate |
| 1303 | Tris(methylphenyl) phosphate (TMPP) |
| 1336 | Aldicarb |
| 1359 | Permethrin |
| 1414 | Allethrin |
| 1584 | Tris(2-chloroethyl) phosphate (TCEP) |
| 1588 | Dieldrin |
| 1601 | Deltamethrin |
| 1636 | Nicotine |
| 1717 | Parathion |
| 1758 | Benomyl |
| 1978 | Kepone |
| 1996 | Methyl parathion |
| 2411 | Glycerol |
| 2459 | Saccharin |
| 2471 | Diethylene glycol |
| 2546 | Ibuprofen |
| 2782 | Sodium benzoate |
| PC | Chlorpyrifos |

**1. Movement-Over-Time Plots**

This download contains per-plate movement-over-time visualizations.

- Each panel displays the median distance moved per minute for viable larvae treated with vehicle control or with each test concentration.
- Files are organized by laboratory (e.g., Lab-A_movement_plate.pdf) as well as are organized by chemical for all laboratories (e.g., Lab-ABCD_movement_plate.pdf).

**2. Plate Data (Raw Data)**

This download contains the raw plate-level zebrafish LDTT data that passed all QC criteria.

- Includes distance moved per minute data and developmental toxicity observations for each embryo.
- Files are stored in RDS (or TSV) format and organized by laboratory.
- One XLSX file includes all four-laboratory data.

**3. Endpoint Data**

This download contains calculated analytical endpoints (DNT and DT) for each plate.

- Files are provided in RDS format (or TSV) format and organized by laboratory.
- Two XLSX file includes all four-laboratory DNT and DT data.

**4. Potency Data**

This download contains benchmark concentration (BMC) and activity concentration at cutoff (ACC) results for both the zebrafish LDTT and the DNT-IVB assays.

- Data include full potency outputs and intermediate summary datasets.
- The complete potency dataset is provided in an Excel workbook containing the following sheets:

DNT-IVB Data (33 compounds)

- endpoint (DNT-IVB, 33)
- assay-summary (DNT-IVB, 33)
- compound-summary (DNT-IVB, 33)

Zebrafish LDTT Data (All 33 compounds)

- endpoint (Zebrafish, 33)
- plate-summary (Zebrafish, 33)
- lab-summary (Zebrafish, 33)
- compound-summary (Zebrafish, 33)

Zebrafish LDTT Data (Subset of 19 compounds with matching concentration ranges across laboratories)

- endpoint (Zebrafish, 19)
- plate-summary (Zebrafish, 19)
- lab-summary (Zebrafish, 19)

Datasets used for Figures 4–6

- compound-summary (DNT-IVB, 33)
- compound-summary (Zebrafish, 33)
- lab-summary (Zebrafish, 19)
